# Synthesis and Thermotropic Phase Behavior of Four Glycoglycerolipids

**DOI:** 10.3390/molecules181113546

**Published:** 2013-11-01

**Authors:** Wouter F. J. Hogendorf, Vivien Jagalski, Thomas G. Pomorski, Mikael Bols, Marité Cárdenas, Christian M. Pedersen

**Affiliations:** 1 Department of Chemistry, University of Copenhagen, Universitetsparken 5, DK-2100 Copenhagen Ø, Denmark; 2 Department of Plant and Environmental Sciences, University of Copenhagen, Thorvaldsensvej 40, Frederiksberg C 1871, Denmark

**Keywords:** glycolipid synthesis, calorimetrics, biomimetic membranes, physiological conditions

## Abstract

Four glycoglycerolipids with different head groups have been synthesized and their physicochemical properties studied. The lengths of the head groups from a mono-saccharide to a trisaccharide, in addition to the anomeric stereochemistry for the smaller glycoglycerolipids, have been modified. The synthesis has been optimized to avoid glycerol epimerization and to allow up-scaling. The physicochemical properties of the glycoglycerolipids were studied and a strong de-mixing of the gel-phase, depending on the head-group, was observed.

## 1. Introduction

Glycolipids play a central role in plants, microorganism and animals. Glycolipids consist of a carbohydrate head group linked to a lipid, typically a diacylglycerol (glycoglycerolipid), ceramide (glycosphingolipid) or prenol. Any given organism can vary both the lipid part and the head group of the glycolipid in order to meet changes in the environmental and physiological conditions [1,2] For example, *Acholeplasma laidlawii* strain A can incorporate supplied fatty acids of a fixed composition when grown under conditions at which the cell’s endogenous fatty acid synthesis were impaired. This organism responds by changing the composition of the polar part (the carbohydrate) of its glycoglycerolipids in order to balance the cell membrane composition of lamellar- and non-lamellar forming glycolipids. The balance between lamellar and non-lamellar forming glycoglycerolipids is critical for the growth of bacterial cells [3,4]. It has been found that, in the case for *A. laidlawii*, glycolipids with one carbohydrate moiety or one acyl group (*i.e.* monoglucosyl-diacylglycerol, monoacylmonoglucosyldiacylglycerol and monoacyl-diglucosylglycerol) form non-lamellar structures of the inverse type, whereas diglucosyldiacylglycerol was found to be lamellar forming [5]. Non-lamellarity is favored upon increased lipid acyl chain length or saturation. The non-lamellar forming glycoglycerolipids are thought to be important for the formation of non-bilayer structures and bilayers with small radius of curvature. Moreover, glycolipids have typically very high melting temperatures (the temperature at where the main phase transition from the gel to the fluid liquid crystalline phase occurs) as compared to other lipids due to intra hydrogen bonding among the sugar groups [6,7,8].

In eukaryotes, glycolipids comprise a small but vital fraction (10%–20%) of the membrane lipids [9] that serve important biological functions in signal transduction, as anchors for cell surface proteins in cascade pathways in immune response and cell communication, among others [10]. Most of the research in the glycolipid field focuses on the direct impact on signaling pathways or immunological reactions [11]. However, the underlying molecular mechanisms of the effects caused by glycolipids on the physicochemical properties of cellular membranes has been poorly studied so far. In part, this is due to the difficulty of obtaining chemically well-defined purified glycolipid fractions in large quantities, as natural isolates are heterogeneous and difficult to purify.

As an alternative approach to lipid extraction from cellular membranes, chemical synthesis of glycolipids is an active field of research. Special focus has been given to the glycolipids found in pathogens as these are often found to be immunogenic. Currently, lipopolysaccharides (LPSs) are under investigation due to their great potential as vaccine components (they are also known as the endotoxin of Gram-negative bacteria) [12]. The Gram positive counterpart—lipoteichoic acids (LTAs) have so far received less attention and their biological roles remain mostly unclear and have been heavily discussed for decades. One of the major problems regarding the determination of LTAs’ biological activity was found to be inappropriate purification methods leading to partly decomposed glycolipids, or samples containing immunogenic lipoproteins. The biological role of LTA was recently confirmed by using synthetic LTA [13,14] from *Streptococcus pneumonia* [15] and *Staphylococcus aureus* [16,17]. Because well-defined synthetic glycolipids are required to achieve reliable biological results, a demand for more structures and derivatives has arisen. We are currently working on the total synthesis of LTA from *Clostridium difficile*. As a part of the synthesis of the glycoglycerollipid anchor part, the general procedure for the synthesis of gentiobiosyl diacylglycerol has been optimized for purity and up-scaling. These improvements will be discussed in the following paragraphs.

The similarity between the lipid anchor and some of the simple glycoglycerolipids found in plants and microorganisms prompted us to investigate the effect of these smaller glycoglycerolipids, formed by the lysis of LTA, on the physicochemical properties of model cell membranes. As a model membranes system we choose a well-described combination of two glycerophospholipids: 1,2-di-myristoyl-phosphatidylcholine (DMPC) and 1,2-di-myristoyl-phosphatidylserine (DMPS) at a biologically relevant proportion (cellular membranes usually carry a net negative charge that ranges from 5 to 25 mol%) [9]. In order to directly study the influence of the head group on the thermotropic properties of the lipid membrane, model compounds were synthesized with 1,2-di-myristoyl-*sn*-glycerol as the lipophilic part. Thus, the contribution of the acyl chain is kept constant among the various lipid species studied in such a way that only the carbohydrate head contribution is systematically varied. Four head groups were chosen: α- and β-glucopyranoside (found in various organisms [18], β-gentiobiose (found in lipid anchor from *S. aureus*) and 6-*O*-β-d-glucopyranosyl gentiobiose (gentiotriose; corresponding to the lipid anchor from *C. difficile*). Vesicles were prepared with a fixed molar composition of DMPC, DMPS and each of the four glycoglycerolipids (79, 8 and 13 mol%, respectively) and studied by a combination of differential scanning calorimetry (DSC) and confocal scanning fluorescence microscopy (CSFM).

## 2. Results and Discussion

### 2.1. Synthesis of the Glycoglycerolipids

**Scheme 1:**
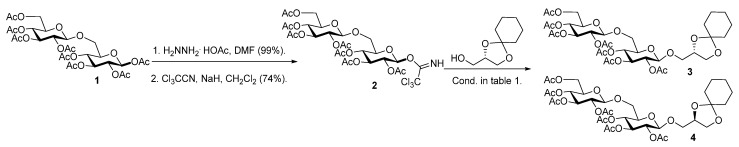
Synthesis of the gentiobiosyl donor **2** and its glycosylation with 1,2-*O*-cyclohexylidene glycerol.

Despite a large number of diacylglycerol-containing glycolipids that have been synthesized through the years, the chemistry is still not straight forward. Glycosylation of a ketal (or acetal) protected glycerol remains an obstacle for a fast and high yielding synthesis of the common glycolipids. Methods [19,20] have been developed, where more persistent protective groups on the glycerol acceptor or other modifications are used, but due to their easy access and orthogonality to other common protective groups, the ketals are still preferred. The main problem by using ketals (or acetals) is the acid catalyzed (Lewis or Brønsted) epimerization of the glycerol part that takes place under practically all glycosylation methods and causes difficulties in the purification [21,22,23,24]. The problem with epimerization seems less pronounced when having neighboring group participation [25] or ortho-esters. However, to our surprise it turned out to be a major problem when using the gentiobiose donor **2** (Scheme 1) which is highly attractive since it can be prepared from commercially available gentiobiose peracetate **1** in 2 simple steps [26]. The initial glycosylation results gave up to 25% of the glycerol epimer **4**, which is seen as additional signals in the NMR spectra as a doubling of the signals in the ^1^H-NMR at 4.49–4.54 ppm and in the ^13^C-NMR two additional peaks at 34.8 and 36.4 ppm. (see Supplementary materials). The epimer **4** could not be chromatographically separated from the desired epimer **3** (Scheme 1). To improve the glycosylation the conditions were optimized.

Since both the donor **2** and the enantiomerically pure cyclohexylidene glycerol are readily available building blocks, it was decided to focus the optimization on the promoter system and the purification methods. Initial studies, using TMSOTf as the promoter in CH_2_Cl_2_, revealed that the yields were improved by lowering the temperature from 0 to −40 °C and the epimer formation was limited as well (from 25% to 10%). Lowering the temperature further (−60 °C) did neither improve the overall yield and the anomeric selectivity further (Table 1). Besides the anomeric selectivity, which to some extent is controlled by neighboring group participation and the undesired glycerol epimerization, ortho-ester formation was observed as a major side reaction. With up to at least eight different glycosylation products formed—all with similar retention times, TMSOTf was abandoned as the promoter system. Changing the promoter to BF_3_×OEt_2_, which should decrease the amount of acid catalyzed epimerization [27], resulted in similar yields and amounts of the undesired α-anomer. The epimerization was however less dominant and the formation of orthoesters likewise. The optimal condition was found to be −35 °C with a slow heating to 0 °C, where the reaction was quenched with triethylamine. With the lower amount of side products formed it was possible to selectively crystallize the product out from the crude reaction mixture, using a mixture of ethyl acetate and petroleum ether, in a very satisfying 70% yield (Table 1).

**Table 1:** Conditions for the gentiobiosylation of 1,2-*O*-isopropylidene glycerol.

T (°C)	Promotor	Yield (%) ^a^	α-x (%) ^b^	Epimer (%) ^c^
0	TMSOTf	63	5	25
−40→0	TMSOTf	75	5	10
−60→0	TMSOTf	84	5–10	10–15
−60→0	BF_3_.OEt_2_	84	5–10	5
−20→−10	BF_3_.OEt_2_	76	5	10
−35→0	BF_3_.OEt_2_	70 ^d^	0	0

^a^ Mixture of glycosylation products isolated by column chromatography; ^b^ Estimated (from ^1^H/^13^C-NMR) amount of the α-anomer; ^c^ Estimated amount (^1^H/^13^C-NMR) of glycerol epimerization; ^d^ Pure product **3** crystallized from the crude reaction mixture.

**Scheme 2:**
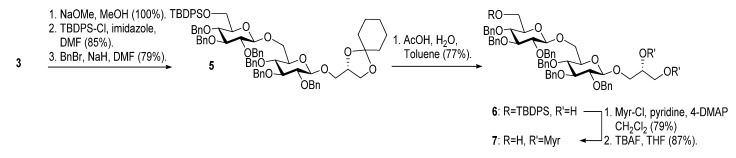
Synthesis of gentiobiosyl building block **7**.

The optimized procedure paved the way for unlimited up-scaling (so far **3** can be obtained in multiple grams). With the access to **3** secured, the acetyl groups were removed under Zemplén conditions, and a 6'OTBDPS group selectively installed in 85% yield. As persistent protective groups benzyls were preferred in order to achieve a one-step global deprotection at the end of the synthesis. Removal of the cyclohexylidene protective group on **5** gave diol **6** (Scheme 2).

**Scheme 3:**
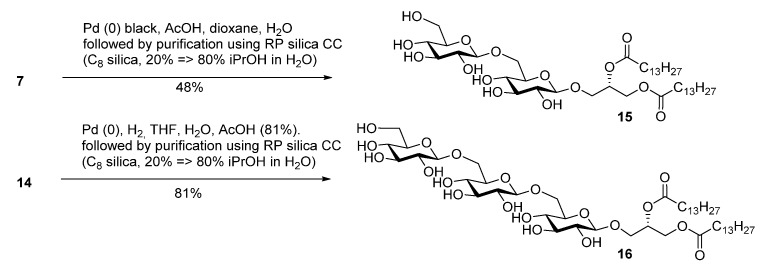
Global deprotection to give the glycolipids **15 **(β-diglycolipid) and **16** (β-triglycolipid).

**Scheme 4:**
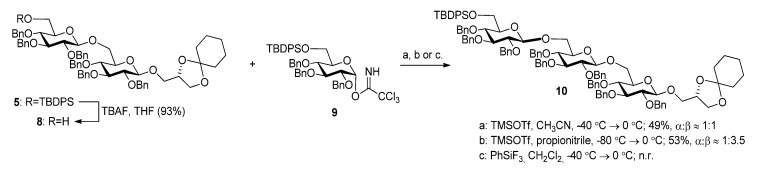
Synthesis of pseudotetrasaccharide **10**.

Myristoylation followed by TBAF-mediated TBDPS removal gave **7** (Scheme 2). The hydrogenolytic debenzylation of **7** was performed in a dioxane/water/AcOH mixture using palladium black as the catalyst. The final glycolipid **15** could be purified by reverse phase silica gel column chromatography (Scheme 3). Removal of the TBDPS group in **5** afforded the pseudotrisaccharide acceptor **8** ready for the chain prolongation by glycosylation or, for example, introduction of a glycerol phosphate oligomer (*S. aureus* LTA) (Scheme 4).

**Scheme 5:**
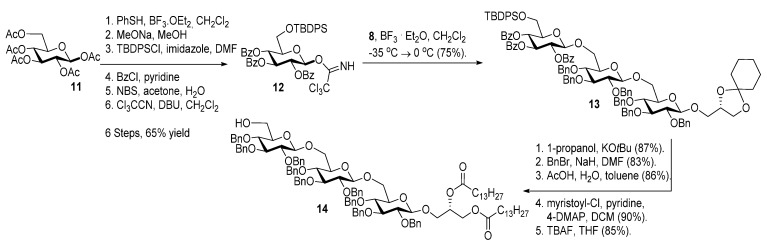
Synthesis of donor **12** [32] and its glycosylation with pseudotrisaccharide **8**.

As with the pseudotrisaccharide acceptor **8** benzyl groups were preferred as persistent protective groups in the donor **9** [28] (Scheme 4). A 6-*O*-TBDPS was again installed in order to allow future ligation to the repeating unit via a phosphate linkage for the *C. difficile* LTA synthesis. Since the benzyl groups in **9** do not participate under glycosylation conditions, the anomeric selectivity relied on the nitrile effect [29]. Therefore, the glycosylation of acceptor **8** with donor **9** was initially explored using MeCN as the solvent, but no selectivity was observed. Changing to propionitrile, which has a lower melting point, improved the selectivity to 1:3.5 (α:β). An attempted PhSiF_3_-catalyzed glycosylation [30] proved unsuccessful as no reaction occurred (Scheme 4). Due to the difficulties in separating the anomers, a more selective glycosylation was needed. To ensure high selectivity the benzyl protective groups were exchanged with benzoyl moieties, which are known to be more robust than acetyl groups under (Lewis) acidic conditions [31] Donor **12** was synthesized from per-*O*-acetyl glucose **11** in 6 steps with an overall yield of 65% (see Scheme 5). The glycosylation of gentiobiosyl acceptor **8** could then be performed uneventfully in CH_2_Cl_2_ giving 75% of the anomerically pure pseudotetrasaccharide **13** (Scheme 5).

Debenzoylation was performed using 1-propanol in combination with potassium *tert*-butoxide in order to better dissolve the reactant and to simplify the purification by chromatography. Benzylation and deprotection of the cyclohexylidene followed by introduction of the myristoyl moieties to give the fully protected glycolipid in an overall good yield and excellent purity. Desilylation liberated the 6″O—the linkage point for the repeating unit, to give **14**. To obtain the fully deprotected lipid anchor, the benzyl groups were hydrogenolyzed using palladium black in a THF/water/AcOH mixture (Scheme 3). Lipid anchor **16** was purified by reverse phase column chromatography to give the pure product in 81% yield.

**Figure 1:**
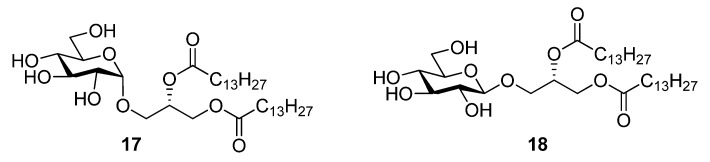
Glycolipids **17** (α-monoglycolipid) and **18** (β-monoglycolipid).

The 1,2-di-*O*-myristoyl-3-*O*-α-d-glucopyranosyl-*sn*-glycerol **17** (Figure 1) was synthesized following the procedure developed for the synthesis of the lipid anchor from *S. pneumonia* [33] and 1,2-di-*O*-myristoyl-3-*O*-β-d-glucopyranosyl-*sn*-glycerol **18** (Figure 1) was synthesized from the peracetylated glucopyranosyl trichloroacetimidate donor following the procedure describe above for **3** [18]. Both glycoglycerolipids are also found in bacteria [34,35].

### 2.2. Thermotropic Behavior of Glycoglycerolipid and Glycerophospholipids

**Figure 2:**
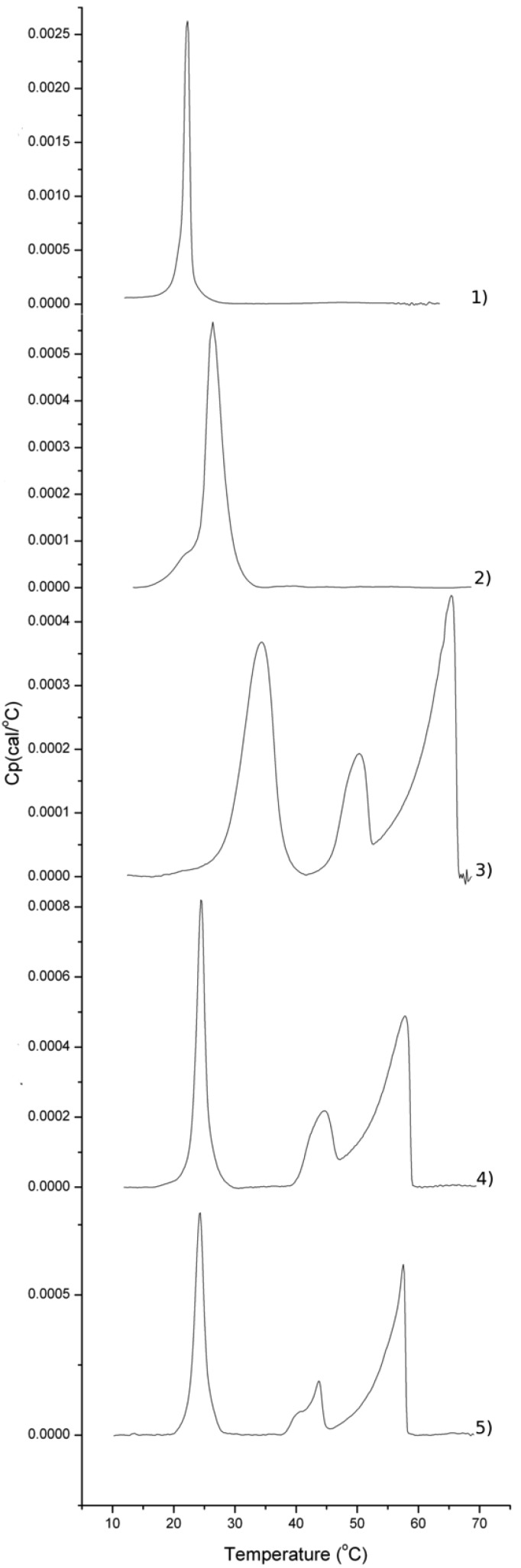
DSC thermograms for (1) DMPS/DMPC, (2) DMPS/DMPC/β-monoglycolipid **18**, (3) DMPS/DMPC/α-monoglycolipid **17**, (4) DMPS/DMPC/β-diglycolipid **15**, (5) DMPS/DMPC/β-triglycolipid **16**. Vesicles at the compositions given in Table 1.

Small unilamellar vesicles were prepared at various compositions (Table 2) and their thermotropic behavior was studied by differential scanning calorimetry (DSC), see Figure 2. A brief description of the method of preparation can be found in the supporting information. DSC measurements were performed in duplicates and ran through two cycles for up scans (10→70 °C) and down scans (70→10 °C) to evaluate whether there was any effect on the mixing behavior of the lipids triggered by the preparation method. The two up/down cycle scans showed no hysteresis effects, suggesting that the method of preparation did not have a significant effect on their thermotropic behavior.

**Table 2:** Composition of the lipid vesicle samples used for DSC studies in this work.

Sample/mol%	DMPC	DMPS	Glycoglycerolipid
Glycerophospholipids	92	8	0
18	0	0	100
18	79	8	13
17	79	8	13
15	79	8	13
16	79	8	13

For glycerophospholipids and for the pure β-monoglycolipid vesicles, a single endothermic peak occurs that corresponds to the first order gel-to-liquid disordered phase transition. The DSC thermograms for pure DMPC, DMPS and β-monoglycolipid vesicles (Supporting Information Figure 1) are in excellent agreement with previous literature [7,36,37]. Interestingly, the melting temperature for 1,2-di-*O*-tetradecyl-3-*O*-methyl-β-d-glucopyranosyl-*sn*-glycerol, a close analogue to β-mono-glycolipid **18**, is dramatically higher (T_m_ = 60 °C) than for our β-monoglycolipid (T_m_ = 45 °C) [38] suggesting that the distance from the sugar unit to the lipid bilayer is a key parameter for the induction of high T_m_.

Lipid mixtures can behave ideally or can have preferential interactions. For ideal mixtures, the ideal melting temperature, T_m,ideal_, can be calculated from weighted average of each lipid T_m_ in the mixture. The mixing of DMPC and DMPS is ideal since T_m,experimental_ = 24.5 ± 0.2 °C is comparable to T_m,ideal_ = 24.3 °C. Ideal mixing for DMPC and DMPS has been previously proposed in the literature [39]. A different scenario is found for the glycerophospholipids-glycoglycerolipid mixtures, for which ideal mixing was only observed when the β-monoglycolipid **18** was used (T_m,ideal_ = 27 °C while T_m,experimental_ = 26.5 ± 0.2 °C). The shape of the peak, though, became more asymmetric suggesting that the phase transition was less cooperative than for DMPC-DMPS mixtures.

For the α-monoglycolipid **17**, on the other hand, a dramatic non-ideal behavior was observed. In this case, three endothermic peaks are observed suggesting three major phase transitions. The same qualitative thermotropic behavior occurs for mixtures with β-di- or β-tri-glycolipids (**15** and **16**). DSC thermograms of structurally similar glycolipids gave a single endothermic transition at around 55–60 °C [6]. Therefore, the phase transition occurring about 57 °C should correspond to a glycolipid rich domain. The high T_m_ for glycolipids is typically ascribed to strong intermolecular interactions between their headgroups [6,7,8]

As mentioned above, the appearance of three endothermic peaks strongly indicates demixing in the lipid bilayers for all glycolipid used except for β-monoglycerolipid **18**. For α-monoglycolipid **17**, the first, second and third phase transition have a corresponding T_m,1st_ = 28, T_m,2nd_ = 44 and T_m,3rd _= 57 °C respectively. The main difference in their DSC thermograms for β-di- or β-tri-glycolipid containing mixtures is a shift of the first peak of lower T_m,1st_ (24.5 ± 0.2 °C). For ideal glycolipid containing mixtures at the composition shown in Table 1, the T_m,ideal_ should be around 28.4 °C assuming the pure glycolipids to have a T_m,glycolipid _ = 58 °C.

These are up-scans representative for duplicates of the same sample. Each sample was run on two up/down scan cycles and no major differences were observed on the thermograms.

There are several explanations to this strong demixing behavior. On one hand, there could be a split population of vesicles with very distinct lipid compositions. This is supported by the fact that the T_m,1st peak_ is so close to the T_m,ideal_ (28.4 °C) implying that there might exist a population of vesicles with an ideal behavior at least for α-monoglycolipid. The T_m_ for the second peak (T_m,2nd _ = 44 °C) and third peak (T_m,3rd_ = 57 °C) could potentially correspond to vesicles enriched with DMPS (T_m,DMPS_ = 39.5 °C) and glycolipids. Vesicles with distinct composition at the single molecule level have been detected earlier [40,41,42].

In an alternative scenario, each vesicle could contain several domains of distinct composition. In this case, the first peak should correspond to the main phase transition for a DMPC-enriched region. This is supported by the fact that for mixtures containing β-di- or β-tri-glycolipids (**15** and **16**), the T_m,1st_ shifts to lower values that are closer to the T_m,ideal_ (24.3 °C) assuming a domain composed only by DMPC and DMPS. Furthermore, domain formation is strongly supported by the highly asymmetric shape of the two peaks with highest T_m_ that typically imply poor cooperativity during the phase transition.

In our glycolipid containing mixtures, the strong hydrogen network formed by the glycolipids could induce domain formation that, upon increasing temperature, could undergo several transformations from a glycolipid rich gel phase into a smectic type phase (liquid ordered phase) and then finally into a liquid-disordered phase. Thus, the three phase transition observed for our mixtures with glycolipids (except for β-monoglycolipid **18**) may correspond to the gel to liquid disordered melting of the DMPC rich phase (T_m,1s_), the gel to liquid ordered melting of the glycolipid rich region (T_m,2nd_), and finally the melting of this last domain into the liquid disordered phase (T_m,3rd_). In this respect, glucose based surfactants can lead to more than one melting point in a bilayer caused by the hydrogen bonding ability of glucose head groups [43,44], and sub gel phases have also been observed for pure glycolipid vesicles in the past [7].

Glycolipids could potentially form complexes with lipids of smaller headgroup for which the large sugar head shadows smaller headgroups of these lipids leading to hydrophobic shielding in a mechanism similar to so-called “umbrella effect” [45]. The shift of T_m,1st_ to values closer to the pure T_m_ DMPC for the β-di and β-triglycolipid containing mixtures suggest there is a preferential complexation with DMPG for these cases. Indeed, phosphatidylserine (PS) lipids promote the incorporation of glycolipids in lipid bilayers while phosphatidylcholine (PC) inhibited their incorporation in fibroblasts [46].

**Figure 3:**
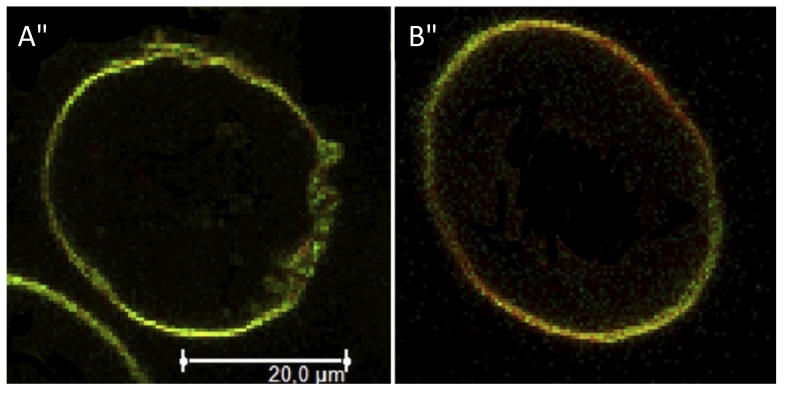
Confocal scanning fluorescence microscopy images for giant unilamellar vesicles containing β-diglycolipid **15** at 22 °C (**A**) and 37 °C (**B**). Vesicles composed of DMPC/DMPS/**5** labeled with 0.1% DiI C18 (green) and 0.1% DiIC18 (red) were prepared as described in the supporting information. Color merged images were shown.

Confocal scanning fluorescence microscopy was used to image our mixed vesicles containing β-diglycolipid **15**, see Figure 3. In this case, giant unilamellar vesicles were prepared since they allow for visualization of micron sized long-lived domains using two different lipid dyes that partition preferentially in fluid (DiIC12) or gel (DiDC18) domains [47].

Details about the vesicle preparation are found in the experimental section and the chemical structures of these dyes are given in the Supporting Information. The vesicles are tethered to a surface to allow for imaging during prolonged times. However, the strength of binding to the surface was weakened upon increasing the temperature above 37 °C and thus we could only image below and above the peak for the first phase transition (that should correspond to the DMPC rich domain). The images show an uneven distribution of the dyes with regions enriched with the red dye (DiDC18), which grow in size with temperature: the uneven dye distribution indicates that domains of different fluidity exist at both temperatures. A vesicle composed mainly of DMPC-DMPS and displaying a single-phase transition should contain an uneven dye distribution at 22 °C and even dye distribution 37 °C, since at the onset of first phase transition occurs around 21 °C and is completed at 37 °C. Thus, these images clearly show that, at 37 °C, domains of distinct lipid composition and fluidity exist in a single vesicle. These domains should be either enriched in (i) glycerophospholipids (green areas)—and be probably in the liquid disordered phase—and (ii) enriched with glycoglycerolipids (red areas)—and probably in the gel phase.

Finally, the thermotropic behavior of vesicles containing β-monoglycolipid differed significantly from the other glycolipids studied. In this case, almost ideal mixing was observed. The main structural difference is the configuration of the sugar group, which appears in a linear configuration or pointing straight away from the lipid bilayer occupying a smaller surface area. This conformation might impair hydrogen bonding with other glycolipids or lipids of smaller head-group (for instance DMPS) giving an ideal mixture in this case. This is supported by the significantly higher T_m_ found for the close analogue 1,2-Di-*O*-tetradecyl-3-*O*-methyl-β-d-glucopyranosul-sn-glycerol, for which only an extra methyl group allows for higher mobility of the headgroup. Earlier, it was suggested that differences in T_m_ of glycolipids could arise from the specific conformation of the sugar head-group and their capability to form hydrogen bonding with other lipids [48].

## 3. Experimental

### 3.1. Synthesis

All chemicals (Merck, Carbosynth, Sigma-Aldrich) were used as received and reactions were carried out dry, under an argon atmosphere, at ambient temperature, unless stated otherwise. Dry solvents were taken from a solvent purification system. Glassware used for water-free reactions were dried for 12 h at 120 °C before use. Column chromatography was performed on ROCC silica gel 60 (0.040–0.063 mm). TLC analysis was conducted on HPTLC aluminium sheets (Merck, silica gel 60, F245). Compounds were visualized by UV absorption (245 nm), by spraying with 20% H_2_SO_4_ in ethanol or with a solution of (NH_4_)_6_Mo_7_O_24_•4H_2_O 25 g/L and (NH_4_)_4_Ce(SO_4_)_4_•2H_2_O 10 g/L, in 10% aqueous H_2_SO_4_ followed by charring at +/− 150 °C. Optical rotation measurements (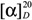) were performed on a Perkin Elmer 341 polarimeter (Sodium D-line, λ = 589 nm) with a concentration of 10 mg/ml (c = 1), unless stated otherwise. ^1^H- and ^13^C-NMR spectra were recorded with a Bruker 500 MHz spectrometer (500 and 125 MHz respectively). Chemical shifts (δ) are reported in ppm relative to the residual solvent signal of either CDCl_3_ (δ = 7.26 for ^1^H-NMR and 77.0 for ^13^C-NMR) or CD_3_OD (δ = 3.31 for ^1^H-NMR and 49.0 for ^13^C-NMR). NMR assignments were based on COSY and HSQC NMR experiments. High-resolution mass spectral (HRMS) data were obtained on an electrospray (ESI) mass spectrometer analyzing time-of-flight (Q-TOF instrument from Micromass).

*Gentiobiosyl Derivative* **3**


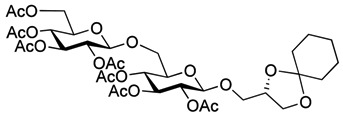


To a solution of gentiobiosyl trichloroacetimidate **2** (7.04 g, 9.01 mmol) and 1,2-*O*-cyclohexylidene-sn-glycerol (3.11 g, 18.0 mmol) in CH_2_Cl_2_ (75 mL) was added a batch of freshly activated powdered MS3Å and the resulting suspension stirred for 15 min. The mixture was cooled to −35 °C and a catalytic amount of boron trifluoride diethyl etherate (55.6 µL, 64 mg, 0.45 mmol) was added. The reaction was allowed to stir for 90 min while slowly warming up to 0 °C. Triethylamine (0.5 mL) was added and the slurry filtered over Celite. The filtrate was concentrated under reduced pressure and the residue crystallized from a mixture of EtOAc (150) and PE (500 mL), giving pseudotrisaccharide **3** (4.97 g, 6.29 mmol, 70%) as a white solid. Mp.: 190 °C; 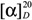 (CHCl_3_): −15.8; ^1^H-NMR: δ = 1.33–1.43 (m, 2H, CH_2_ cyclohexylidene), 1.53–1.63 (m, 8H, 4 × CH_2_ cyclohexylidene), 1.98–2.10 (7 × s, 21H, 7 × CH_3_ acetyl), 3.59–3.71 (m, 4H, H-5, H-5', 2 × H-6), 3.78 (dd, 1H, *J* = 5.9 Hz, 8.3 Hz, CH*H* glycerol), 3.83–3.88 (m, 2H, CH_2_ glycerol), 3.99 (dd, 1H, *J* = 6.4 Hz, 8.2 Hz, C*H*H glycerol), 4.12 (dd, 1H, *J* = 2.3 Hz, 12.3 Hz, H-6'), 4.21–4.24 (m, 1H, CH glycerol), 4.27 (dd, 1H, *J* = 4.8 Hz, 12.4 Hz, H-6'), 4.57–4.61 (2 × d, 2H, *J* = 8.0 Hz, *J* = 8.0 Hz, H-1, H-1'), 4.87–5.01 (m, 3H, H-2, H-2', H-4), 5.07 (t, 1H, *J* = 9.7 Hz, H-4'), 5.15–5.21 (m, 2H, H-3, H-3'); ^13^C-NMR: δ = 20.6–20.7 (7 × CH_3_ acetyl), 23.8, 24.0, 25.1, 34.6, 36.2 (5 × CH_2_ cyclohexylidene), 61.8 (C-6'), 65.8 (CH_2_ glycerol), 68.1 (CH_2_ glycerol), 68.3 (C-4'), 68.9 (C-6), 69.1 (C-4), 71.1, 71.2 (C-2, C-2'), 72.0 (C-5'), 72.8 (C-3, C-3'), 73.3 (C-5), 73.9 (CH glycerol), 100.7, 100.8 (C-1, C-1'), 109.9 (C_q_ cyclohexylidene), 169.2, 169.3, 169.4, 169.6, 170.2, 170.2, 170.6 (7 × C_q_ acetyl); HRMS: C_35_H_50_O_20_ + Na^+^ requires 813.2788, found 813.2785.

*Conversion of* **3** *to* **5**

Intermediate A


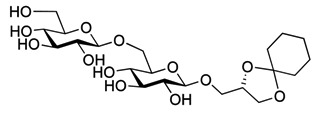


To a solution of compound **3** (5.31 g, 6.72 mmol) in MeOH (67 mL) was added sodium methoxide (25% solution in MeOH, 0.50 mL) after which the mixture was stirred for 1 h. The reaction was quenched with amberlite H^+^ and filtered. The filtrate was concentrated *in vacuo* giving intermediate A (3.33 g, 6.71 mmol, 100%) as an amorphous off-white solid. 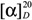 (MeOH): −24.1; ^1^H-NMR (CD_3_OD): δ = 1.37–1.46 (m, 2H, CH_2_ cyclohexylidene), 1.56–1.68 (m, 8H, 4 × CH_2_ cyclohexylidene), 3.18–3.25 (m, 2H, H-2, H-2'), 3.28–3.39 (m, 5H, H-3, H-3', H-4, H-4', H-5'), 3.45–3.49 (m, 1H, H-5), 3.62 (dd, 1H, *J* = 6.0 Hz, 10.6 Hz, CH*H* glycerol), 3.68 (dd, 1H, *J* = 5.3 Hz, 11.9 Hz, H-6'), 3.77–3.84 (m, 2H, H-6, CH*H* glycerol), 3.86–3.94 (m, 2H, H-6', C*H*H glycerol), 4.08 (dd, 1H, *J* = 6.4 Hz, 8.4 Hz, C*H*H glycerol), 4.16 (dd, 1H, *J* = 2.0 Hz, 11.5 Hz, H-6), 4.32 (d, 1H, *J* = 7.8 Hz, H-1), 4.33–4.37 (m, 1H, CH glycerol), 4.38 (d, 1H, *J* = 7.8 Hz, H-1'); ^13^C-NMR (CD_3_OD): δ = 24.8, 25.0, 26.2, 35.9, 37.5 (5 × CH_2_ cyclohexylidene), 62.7 (C-6'), 67.4 (CH_2_ glycerol), 69.9 (C-6), 71.4, 71.6 (C-4, C-4'), 71.6 (CH_2_ glycerol), 75.0, 75.1 (C-2, C-2'), 75.5 (CH glycerol), 77.0 (C-5'), 77.8 (C-5), 78.0 (C-3. C-3'), 104.7, 104.9 (C-1, C-1'), 111.1 (C_q_ cyclohexylidene); HRMS: C_21_H_36_O_13_ + Na^+^ requires 519.2048, found 519.2053.

Intermediate B


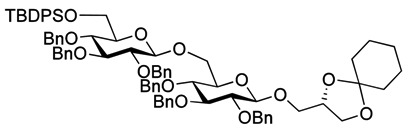


To a solution of intermediate A (3.15 g, 6.34 mmol) and imidazole (1.73 g, 25.4 mmol) in DMF (49 mL) was added TBDPS-Cl (2.44 mL, 2.62 g, 9.52 mmol) after which the mixture was allowed to stir for 1 h. At this point a second batch of TBDPS-Cl (0.33 mL, 0.35g, 1.2 mmol) was added and the reaction stirred for 1h before it was quenched with MeOH (5 mL). The mixture was diluted with EtOAc (300 mL) and washed with water (150 mL). The aqueous layer was extracted once with EtOAc (50 mL) and the combined organic layers washed with water (250 mL) and brine (250 mL). The organic layer was dried with Na_2_SO_4_, filtered, and concentrated under reduced pressure. Purification of the residue by column chromatography (acetone/DCM/MeOH, 1/1/0 → 2/2/1) afforded mono-silylated intermediate B (3.95 g, 5.37 mmol, 85%) as a colourless oil. 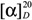 (MeOH): −21.9; ^1^H-NMR (CD_3_OD): δ = 1.06 (s, 9H, *t*Bu), 1.37–1.43 (m, 2H, CH_2_ cyclohexylidene), 1.55–1.62 (m, 8H, 4 × CH_2_ cyclohexylidene), 3.22–3.28 (m, 2H, H-2, H-2'), 3.35–3.45 (m, 5H, H-3, H-3', H-4, H-4', H-5'), 3.47–3.51 (m, 1H, H-5), 3.61 (dd, 1H, *J* = 6.0 Hz, 10.5 Hz, CH*H* glycerol), 3.76–3.82 (m, 2H, H-6, CH*H* glycerol), 3.89 (dd, 1H, *J* = 5.1 Hz, 11.1 Hz, H-6'), 3.93 (dd, 1H, *J* = 5.5 Hz, 10.5 Hz, C*H*H glycerol), 4.01–4.07 (m, 2H, H-6', C*H*H glycerol), 4.21 (dd, 1H, *J* = 2.0 Hz, 11.4 Hz, H-6), 4.30–4.35 (m, 2H, H-1, CH glycerol), 4.41 (d, 1H, *J* = 7.8 Hz, H-1'), 7.39–7.44 (m, 6H, H_arom_), 7.74–7.77 (m, 4H, H_arom_); ^13^C-NMR (CD_3_OD): δ = 20.2 (C_q_ *t*Bu), 24.8, 25.0, 26.2 (3 × CH_2_ cyclohexylidene), 27.4 (3 × CH_3_ *t*Bu), 35.9, 37.5 (2 × CH_2_ cyclohexylidene), 64.7 (C-6'), 67.4 (CH_2_ glycerol), 69.4 (C-6), 71.2, 71.4 (C-4, C-4'), 71.7 (CH_2_ glycerol), 75.0, 75.1 (C-2, C-2'), 75.5 (CH glycerol), 77.0 (C-5), 77.8 (C-5'), 78.2, 78.2 (C-3. C-3'), 104.7, 104.7 (C-1, C-1'), 111.0 (C_q_ cyclohexylidene), 128.8, 130.7, 130.8 (CH_arom_), 134.7, 134.9 (2 × C_q_ Ph), 136.7, 136.8, 136.9 (CH_arom_); HRMS: C_37_H_54_O_13_Si + Na^+^ requires 757.3226, found 757.3224.

*Gentiobiosyl Derivative* **5**


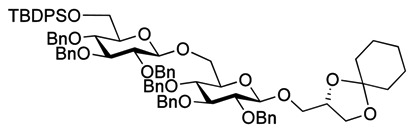


To a cooled (0 °C) solution of intermediate B (3.88 g, 5.28 mmol) in DMF (35 mL) were added benzyl bromide (5.65 mL, 8.13 g, 47.5 mmol) and sodium hydride (40% dispersion in mineral oil, 1.90 g, 47.5 mmol). After stirring for 1 h at rt the mixture was heated to 40 °C and allowed to stir at this temperature for 75 min before the reaction was quenched with MeOH (5 mL). The reaction was diluted with Et_2_O (150 mL) and washed with water (2 × 75 mL) and brine (75 mL). The organic layer was dried (Na_2_SO_4_), filtered and the volatiles removed under reduced pressure. Purification of the residue by column chromatography (Et_2_O/PE, 0/1 → 1/3) gave hexabenzylated derivative **5** (5.33 g, 4.18 mmol, 79%) as a pale yellow oil. 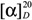 (CHCl_3_): +10.4; ^1^H-NMR: δ = 1.05 (s, 9H, *t*-Bu), 1.33–1.40 (m, 2H, CH_2_ cyclohexylidene), 1.51–1.58 (m, 8H, 4 × CH_2_ cyclohexylidene), 3.31 (dt, *J* = 2.8 Hz, 9.7 Hz, H-5'), 3.41–3.52 (m, 4H, H-2, H-2', H-4, CH*H* glycerol), 3.56–3.60 (m, 1H, H-5), 3.61-3.71 (m, 4H, H-3, H-3', H-6, CH*H* glycerol), 3.76 (t, 1H, *J* = 9.4 Hz, H-4'), 3.89–3.95 (m, 4H, 2 × H-6', 2 × C*H*H glycerol), 4.15–4.19 (m, 1H, CH glycerol), 4.23 (dd, 1H, *J* = 1.8 Hz, 11.4 Hz, H-6), 4.39 (d, 1H, *J* = 7.8 Hz, H-1), 4.47 (d, 1H, *J* = 7.8 Hz, H-1'), 4.54 (d, 1H, *J* = 11.2 Hz, CH*H* Bn), 4.68–4.83 (m, 6H, 3 × CH_2_ Bn), 4.88–4.95 (m, 4H, 2 × CH_2_ Bn), 5.01 (d, 1H, *J* = 11.2 Hz, CH*H* Bn), 7.16–7.42 (m, 36H, H_arom_), 7.69–7.72 (m, 2H, H_arom_), 7.76–7.78 (m, 2H, H_arom_); ^13^C-NMR: δ = 19.3 (C_q_ *t*Bu), 23.8, 24.0, 25.1 (3 × CH_2_ cyclohexylidene), 26.8 (3 × CH_3_ *t*Bu), 34.8, 36.4 (2 × CH_2_ cyclohexylidene), 62.7 (C-6'), 66.3 (CH_2_ glycerol), 68.2 (C-6), 70.3 (CH_2_ glycerol), 73.9 (CH glycerol), 74.8, 74.8, 74.9, 75.1 (4 × CH_2_ Bn), 75.2 (C-5), 75.6 (CH_2_ Bn), 75.7 (C-5’), 75.9 (CH_2_ Bn), 77.6 (C-4'), 78.1 (C-4), 82.1 (C-2), 82.4 (C-2'), 84.6, 84.9 (C-3, C-3'), 103.7 (C-1), 104.0 (C-1'), 109.9 (C_q_ cyclohexylidene), 127.4–128.4 (CH_arom_), 129.6, 129.6 (CH_arom_), 133.1, 133.6 (2 × C_q_ Ph), 135.6, 135.9 (CH_arom_), 138.1, 138.3, 138.4, 138.5, 138.6, 138.6 (6 × C_q_ Bn); HRMS: C_79_H_90_O_13_Si + Na^+^ requires 1297.6043, found 1297.6042.

*Gentiobiosyl Derivative* **6**


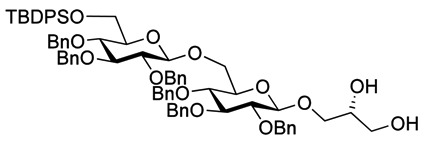


A solution of gentiobiosyl derivative **5** (223 mg, 0.175 mmol) in a mixture of acetic acid (5.5 mL), toluene (1.0 mL) and water (1.5 mL) was stirred for 3 h at 90 °C. After cooling down to rt the mixture was diluted with EtOAc (50 mL) and washed with water (2 × 25 mL), sat. aq. NaHCO_3_ (2 × 25 mL) and brine (25 mL). The organic layer was dried with Na_2_SO_4_, filtered and concentrated *in vacuo*, after which the residue was purified by column chromatography (Et_2_O/PE, 1/3 → 1/0) giving diol **6** (161 mg, 0.135 mmol, 77%) as a colourless oil. 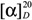 (CHCl_3_): +10.3; ^1^H-NMR: δ = 1.05 (s, 9H, *t*-Bu), 3.33 (dt, *J* = 2.7 Hz, 9.6 Hz, H-5’), 3.39–3.44 (m, 3H, H-2, H-4, CH*H* glycerol), 3.48–3.52 (m, 1H, H-2'), 3.55 (dd, 1H, *J* = 4.0 Hz, 11.4 Hz, CH*H* glycerol), 3.59–3.77 (m, 8H, H-3, H-3', H-4', H-5, H-6, CH glycerol, 2 × C*H*H glycerol), 3.91–3.94 (m, 2H, 2 × H-6'), 4.19–4.22 (m, 1H, H-6), 4.38 (d, 1H, *J* = 7.8 Hz, H-1), 4.47 (d, 1H, *J* = 7.8 Hz, H-1'), 4.53 (d, 1H, *J* = 11.2 Hz, CH*H* Bn), 4.67 (d, 1H, *J* = 10.8 Hz, CH*H* Bn), 4.71–4.93 (m, 9H, 4 × CH_2_ Bn, CH*H* Bn), 4.97 (d, 1H, *J* = 11.0 Hz, CH*H* Bn) 7.16–7.40 (m, 36H, H_arom_), 7.68–7.70 (m, 2H, H_arom_), 7.73–7.76 (m, 2H, H_arom_); ^13^C-NMR): δ = 19.3 (C_q_ *t*Bu), 26.8 (3 × CH_3_ *t*Bu), 62.7 (C-6'), 63.5 (CH_2_ glycerol), 68.3 (C-6), 70.6 (CH glycerol), 73.2 (CH_2_ glycerol), 74.9, 74.9, 75.1, 75.1 (4 × CH_2_ Bn), 75.2 (C-5), 75.7 (CH_2_ Bn), 75.7 (C-5'), 75.8 (CH_2_ Bn), 77.7 (C-4'), 78.2 (C-4), 82.1 (C-2), 82.5 (C-2'), 84.6, 84.7 (C-3, C-3'), 103.9, 103.9 (C-1, C-1'), 127.6–128.1 (CH_arom_), 128.3–128.4 (CH_arom_), 129.6, 129.6 (CH_arom_), 133.1, 133.6 (2 × C_q_ Ph), 135.5, 135.9 (CH_arom_), 137.9, 138.2, 138.2, 138.4, 138.4, 138.5 (6 × C_q_ Bn); HRMS: C_73_H_82_O_13_Si + Na^+^ requires 1217.5417, found 1217.5424.

*Conversion of* **6** *to* 7

Intermediate C


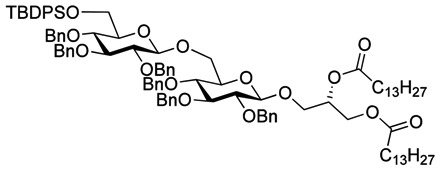


To a solution of diol **6** (149 mg, 0.125 mmol) and pyridine (0.101 mL, 99.2 mg, 1.25 mmol) in DCM (2.5 mL) were added myristoyl chloride (0.101 mL, 91.7 mg, 0.372 mmol) and a catalytic amount of 4-DMAP, respectively. After stirring for 5 h, methanol (1.0 mL) was added and after 10 min of stirring the volatiles were removed under reduced pressure. The residue was taken up in EtOAc (25 mL) and washed with aqueous 1M HCl (10 mL), sat. aq. NaHCO_3_ (10 mL) and brine (10 mL). The organic layer was dried (Na_2_SO_4_), filtered, and concentrated *in vacuo*. The residue was purified by column chromatography (Et_2_O/PE/Et_3_N, 0/1/0.01 → 1/4/0.05), giving intermediate C (160 mg, 99.0 µmol, 79%) as a colourless oil. 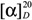 (CHCl_3_): +12.3; ^1^H-NMR): δ = 0.91 (t, 6H, *J* = 6.8 Hz, 2 × CH_3_ myristoyl), 1.08 (s, 9H, *t*-Bu), 1.21–1.37 (m, 40H, 20 × CH_2_ myristoyl), 1.53–1.61 (m, 4H, 2 × CH_2_ myristoyl), 2.20–2.29 (m, 4H, 2 × CH_2_ myristoyl), 3.32–3.36 (m, 1H, H-5'), 3.43–3.46 (m, 1H, H-2), 3.47–3.62 (m, 4H, H-2', H-4, H-5, CH*H* glycerol), 3.63–3.73 (m, 3H, H-3, H-3', H-6), 3.80 (t, 1H, *J* = 9.4 Hz, H-4'), 3.93–3.97 (m, 2H, 2 × H-6'), 4.00 (dd, 1H, *J* = 4.6 Hz, 10.8 Hz, C*H*H glycerol), 4.16 (dd, 1H, *J* = 7.1 Hz, 11.9 Hz, CH*H* glycerol), 4.23–4.28 (m, 2H, H-6, C*H*H glycerol), 4.37 (d, 1H, *J* = 7.8 Hz, H-1), 4.46 (d, 1H, *J* = 7.8 Hz, H-1'), 4.57 (d, 1H, *J* = 11.2 Hz, CH*H* Bn), 4.69–4.87 (m, 6H, 3 × CH_2_ Bn), 4.91–4.98 (m, 4H, 2 × CH_2_ Bn), 5.04 (d, 1H, *J* = 11.2 Hz, CH*H* Bn), 5.15–5.19 (m, 1H, CH glycerol), 7.17–7.44 (m, 36H, H_arom_), 7.71–7.74 (m, 2H, H_arom_), 7.77–7.81 (m, 2H, H_arom_); ^13^C-NMR): δ = 14.1 (2 × CH_3_ myristoyl), 19.3 (C_q_ *t*Bu), 22.7 (2 × CH_2_ myristoyl), 24.8, 24.9 (2 × CH_2_ myristoyl), 26.8 (3 × CH_3_ *t*Bu), 29.1–29.7 (16 × CH_2_ myristoyl), 31.9 (2 × CH_2_ myristoyl, 34.0, 34.2 (2 × CH_2_ myristoyl), 62.7, 62.7 (C-6', CH_2_ glycerol), 68.1 (CH_2_ glycerol), 68.3 (C-6), 69.8 (CH glycerol), 74.7, 74.8, 74.8 (3 × CH_2_ Bn), 75.0 (C-5), 75.1, 75.6 (2 × CH_2_ Bn), 75.7 (C-5'), 75.9 (CH_2_ Bn), 77.6 (C-4'), 78.0 (C-4), 81.9 (C-2), 82.3 (C-2'), 84.5, 84.8 (C-3, C-3'), 103.8, 104.0 (C-1, C-1'), 127.5–128.4 (CH_arom_), 129.5, 129.6 (CH_arom_), 133.1, 133.6 (2 × C_q_ Ph), 135.5, 135.9 (CH_arom_), 138.1, 138.3, 138.3, 138.5, 138.5, 138.6 (6 × C_q_ Bn), 172.9, 173.2 (2 × C_q_ myristoyl).

Protected Glycolipid **7**


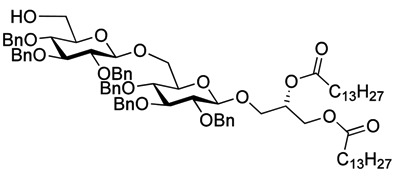


To a solution of intermediate C (142 mg, 88.0 µmol) in THF (1.8 mL) was added TBAF (1.0 M solution in THF, 0.26 mL, 0.26 mmol). After stirring overnight at rt, the volatiles were removed under reduced pressure after which the residue was purified by column chromatography (Et_2_O/PE, 1/4 → 3/2). Semiprotected glycolipid **7** (105 mg, 76.4 µmol, 87%) was obtained as a colourless oil. 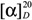 (CHCl_3_): +14.0; ^1^H-NMR: δ = 0.89 (t, 6H, *J* = 7.0 Hz, 2 × CH_3_ myristoyl), 1.23–1.33 (m, 40H, 20 × CH_2_ myristoyl), 1.53–1.61 (m, 4H, 2 × CH_2_ myristoyl), 2.10 (t, 1H, *J* = 6.4 Hz, 6'-OH), 2.20–2.29 (m, 4H, 2 × CH_2_ myristoyl), 3.35 (ddd, 1H, *J* = 2.7 Hz, 4.8 Hz, 9.6 Hz, H-5'), 3.39–3.58(m, 6H, H-2, H-2', H-4, H-4', H-5, CH*H* glycerol), 3.63–3.73 (m, 4H, H-3, H-3', H-6, H-6'), 3.83–3.88 (m, 1H, H-6'), 3.94 (dd, 1H, *J* = 4.6 Hz, 10.9 Hz, C*H*H glycerol), 4.10 (dd, 1H, *J* = 1.4 Hz, 11.3 Hz, H-6), 4.16 (dd, 1H, *J* = 7.0 Hz, 12.0 Hz, CH*H* glycerol), 4.27 (dd, 1H, *J* = 3.5 Hz, 12.0 Hz, C*H*H glycerol), 4.33 (d, 1H, *J* = 7.8 Hz, H-1), 4.47 (d, 1H, *J* = 7.8 Hz, H-1'), 4.55 (d, 1H, *J* = 11.1 Hz, CH*H* Bn), 4.65 (d, 1H, *J* = 11.0 Hz, CH*H* Bn), 4.69 (d, 1H, *J* = 11.1 Hz, CH*H* Bn) , 4.75–4.80 (m, 3H, CH_2_ Bn, CH*H* Bn), 4.82 (d, 1H, *J* = 10.9 Hz, CH*H* Bn), 4.87 (d, 1H, *J* = 11.0 Hz, CH*H* Bn), 4.91–4.96 (m, 4H, 2 × CH_2_ Bn), 5.15–5.19 (m, 1H, CH glycerol), 7.21–7.37 (m, 30H, H_arom_); ^13^C-NMR: δ = 14.1 (2 × CH_3_ myristoyl), 22.7 (2 × CH_2_ myristoyl), 24.9, 24.9 (2 × CH_2_ myristoyl), 29.1–29.7 (16 × CH_2_ myristoyl), 31.9 (2 × CH_2_ myristoyl, 34.1, 34.2 (2 × CH_2_ myristoyl), 62.0 (C-6'), 62.7 (CH_2_ glycerol), 68.0 (CH_2_ glycerol), 68.8 (C-6), 69.9 (CH glycerol), 74.7, 74.8, 74.9 (3 × CH_2_ Bn), 74.9 (C-5), 75.0 (CH_2_ Bn), 75.1 (C-5'), 75.7 (2 × CH_2_ Bn), 77.6 (C-4'), 77.8 (C-4), 81.9 (C-2), 82.1 (C-2'), 84.5, 84.6 (C-3, C-3'), 103.8 (C-1), 103.9 (C-1'), 127.6–128.4 (CH_arom_), 137.9, 138.0, 138.3, 138.3, 138.4, 138.5 (6 × C_q_ Bn), 173.0, 173.3 (2 × C_q_ myristoyl); HRMS: C_85_H_116_O_15_ + Na^+^ requires 1399.8206, found 1399.8209.

*Gentiobiosyl Derivative* **8**


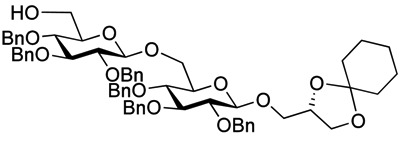


To a solution of silyl ether **5** (2.84 g, 2.23 mmol) in THF (23 mL) was added TBAF (1.0 M solution in THF, 5.6 mL, 5.6 mmol) and the mixture stirred for 2 h before a second batch of TBAF (1.0 M solution in THF, 3.3 mL, 3.3 mmol) was added. After stirring overnight at rt, the volatiles were removed under reduced pressure and the residue was taken up in Et_2_O (100 mL) and washed with water (2 × 50 mL) and brine (50 mL). The organic layer was dried with Na_2_SO_4_, filtered, and concentrated *in vacuo*, after which the crude product was crystallized from MeOH, giving alcohol **8** (2.14 g, 2.06 mmol, 93%) as an amorphous white solid. M.p.: 104–105 °C; 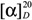 (CHCl_3_): +13.9; ^1^H-NMR: δ = 1.28–1.43 (m, 2H, CH_2_ cyclohexylidene), 1.50–1.60 (m, 8H, 4 × CH_2_ cyclohexylidene), 2.13 (bs, 1H, 6'-OH), 3.33 (ddd, *J* = 2.6 Hz, 4.7 Hz, 9.5 Hz, H-5'), 3.40–3.48 (m, 4H, H-2, H-2', H-4, CH*H* glycerol), 3.50–3.56 (m, 2H, H-4', H-5), 3.60–3.75 (m, 5H, H-3, H-3', H-6, H-6', CH*H* glycerol), 3.81–3.86 (m, 1H, H-6'), 3.89 (dd, 1H, *J* = 4.9 Hz, 10.4 Hz, C*H*H glycerol), 3.93 (dd, 1H, *J* = 6.3 Hz, 8.2 Hz, C*H*H glycerol), 4.04–4.07 (m, 1H, H-6), 4.17–4.22 (m, 1H, CH glycerol), 4.35 (d, 1H, *J* = 7.8 Hz, H-1), 4.48 (d, 1H, *J* = 7.8 Hz, H-1'), 4.53 (d, 1H, *J* = 11.1 Hz, CH*H* Bn), 4.63 (d, 1H, *J* = 11.0 Hz, CH*H* Bn), 4.68 (d, 1H, *J* = 11.1 Hz, CH*H* Bn), 4.72–4.85 (m, 6H, 2 × CH_2_ Bn, CH*H* Bn), 4.89–4.94 (m, 4H, 2 × CH_2_ Bn), 7.18–7.39 (m, 30H, H_arom_); ^13^C-NMR: δ = 23.7, 23.9, 25.1 (3 × CH_2_ cyclo-hexylidene), 34.8, 36.4 (2 × CH_2_ cyclohexylidene), 62.0 (C-6’), 66.4 (CH_2_ glycerol), 68.9 (C-6), 70.3 (CH_2_ glycerol), 73.8 (CH glycerol), 74.7, 74.8, 74.9, 75.0 (4 × CH_2_ Bn), 75.0 (C-5), 75.1 (C-5'), 75.6, 75.6 (2 × CH_2_ Bn), 77.5 (C-4'), 77.9 (C-4), 82.0 (C-2), 82.1 (C-2'), 84.6, 84.6 (C-3, C-3'), 103.6 (C-1), 104.0 (C-1'), 109.9 (C_q_ cyclohexylidene), 127.6–128.4 (CH_arom_), 137.9, 138.0, 138.3, 138.3, 138.4, 138.5 (6 × C_q_ Bn); HRMS: C_63_H_72_O_13_ + Na^+^ requires 1059.4865, found 1059.4878.

*Gentiotriosyl Derivative* **13**


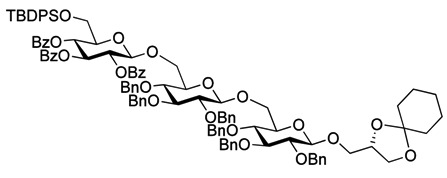


A solution of alcohol **8** (104 mg, 100 µmol) and donor **12** (96.3 mg, 110 µmol) in DCM (2.0 mL), containing freshly activated powdered MS3Å, was cooled to −35 °C. Boron trifluoride diethyl etherate (1.2 µL, 10 µmol) was added and the mixture was allowed to stir 2 h while slowly warming to 0 °C. Et_3_N (0.10 mL) was added, the mixture filtered over Celite and the volatiles removed under reduced pressure. The residue was purified by column chromatography (Et_2_O/PE, 1/9 → 1/1), giving pseudotetrasaccharide **13** (132 mg, 75 µmol, 75%) as a colourless oil. 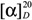 (CHCl_3_): +3.9; ^1^H-NMR: δ = 1.03 (s, 9H, *t*-Bu), 1.23–1.40 (m, 2H, CH_2_ cyclohexylidene), 1.50–1.61 (m, 8H, 4 × CH_2_ cyclohexylidene), 3.35 (dd, 1H, *J* = 7.8 Hz, 9.1 Hz, H-2'), 3.38–3.44 (m, 6H, H-2, H-4, H-4', H-5, H-5', CH*H* glycerol), 3.52–3.56 (m, 1H, H-3'), 3.59–3.63 (m, 2H, H-3, H-6'), 3.70 (dd, 1H, *J* = 6.2 Hz, 8.2 Hz, CH*H* glycerol), 3.72–3.80 (m, 2H, H-5″, H-6), 3.85–3.87 (m, 2H, 2 × H-6″), 3.91–3.95 (m, 2H, 2 × C*H*H glycerol), 4.06–4.10 (m, 1H, H-6'), 4.17–4.25 (m, 2H, H-6, CH glycerol), 4.31 (d, 1H, *J* = 7.8 Hz, H-1), 4.39–4.43 (m, 2H, H-1', CH*H* Bn), 4.48 (d, 1H, *J* = 11.1 Hz, CH*H* Bn), 4.57 (d, 1H, *J* = 11.2 Hz, CH*H* Bn), 4.66–4.73 (m, 4H, 2 × CH_2_ Bn), 4.76 (d, 1H, *J* = 11.0 Hz, CH*H* Bn), 4.84 (d, 1H, *J* = 7.9 Hz, H-1″), 4.87 (d, 1H, *J* = 11.0 Hz, CH*H* Bn), 4.90–4.94 (m, 3H, CH_2_ Bn, CH*H* Bn), 5.57 (dd, 1H, *J* = 7.9 Hz, 9.7 Hz, H-2″), 5.62 (t, 1H, *J* = 9.7 Hz, H-4″), 5.81 (t, 1H, *J* = 9.7 Hz, H-3″), 7.10–7.13 (m, 2H, H_arom_), 7.16–7.41 (m, 42H, H_arom_), 7.51–7.54 (m, 1H, H_arom_), 7.57–7.59 (m, 2H, H_arom_), 7.70–7.72 (m, 2H, H_arom_), 7.81–7.87 (m, 4H, H_arom_), 7.89–7.92 (m, 2H, H_arom_); ^13^C-NMR: δ = 19.1 (C_q_ *t*Bu), 23.8, 24.0, 25.1 (3 × CH_2_ cyclohexylidene), 26.6 (3 × CH_3_ *t*Bu), 34.8, 36.4 (2 × CH_2_ cyclo-hexylidene), 62.8 (C-6″), 66.3 (CH_2_ glycerol), 68.0, 68.0 (C-6, C-6'), 69.2 (C-4″), 70.2 (CH_2_ glycerol), 72.1 (C-2″), 73.3 (C-3″), 73.9 (CH glycerol), 74.3 (C-5'), 74.5, 74.7, 74.8, 74.9 (4 × CH_2_ Bn), 75.2, 75.3 (C-5, C-5″), 75.5, 75.5 (2 × CH_2_ Bn), 77.4 (C-4'), 78.0 (C-4), 82.0 (C-2'), 82.0 (C-2), 84.6 (C-3), 84.7 (C-3'), 101.3 (C-1″), 103.5 (C-1), 103.9 (C-1'), 109.9 (C_q_ cyclohexylidene), 127.5–128.4 (CH_arom_), 128.9, 129.2, 129.4 (3 × C_q_ Bz), 129.6–129.8 (CH_arom_), 133.0 (C_q_ Ph), 133.0 (CH_arom_), 133.0 (C_q_ Ph), 133.1, 133.2 (CH_arom_), 135.5, 135.6 (CH_arom_), 138.1, 138.1, 138.4, 138.5, 138.5, 138.6 (6 × C_q_ Bn), 164.9, 164.9, 165.9 (3 × C_q_ Bz); HRMS: C_106_H_112_O_21_Si + Na^+^ requires 1771.7358, found 1771.7367.

*Conversion of* **13** *to* **14**

Intermediate D 


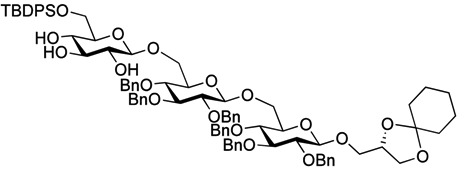


To a solution of gentiotriosyl derivative **13** (840 mg, 0.480 mmol) in 1-propanol (10 mL) was added a catalytic amount of KO*t*Bu after which the reaction was allowed to stir at rt for 2 h. Amberlite H^+^ resin was then added to the reaction until the pH was neutral and the resulting mixture filtered and concentrated under reduced pressure. Purification of the residue by column chromatography (EtOAc/PE, 1/9 → 1/2) gave intermediate D (600 mg, 0.417 mmol, 87%) as a colourless oil. 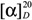 (CHCl_3_): +3.2; ^1^H-NMR: δ = 1.06 (s, 9H, *t*-Bu), 1.28–1.42 (m, 2H, CH_2_ cyclohexylidene), 1.50–1.61 (m, 8H, 4 × CH_2_ cyclohexylidene), 3.30 (dd, 1H, *J* = 8.0 Hz, 9.2 Hz, H-2″), 3.37–3.51 (m, 8H, H-2, H-2', H-3″, H-4, H-4', H-5', H-5″, CH*H* glycerol), 3.54–3.66 (m, 5H, H-3, H-3', H-4″, H-5, H-6'), 3.72–3.78 (m, 2H, H-6, CH*H* glycerol), 3.87–3.93 (m, 2H, 2 × H-6″), 3.93–4.00 (m, 2H, 2 × C*H*H glycerol), 4.04–4.09 (m, 2H, H-6, H-6'), 4.26–4.30 (m, 2H, H-1″, CH glycerol), 4.39 (d, 1H, *J* = 7.8 Hz, H-1), 4.47 (d, 1H, *J* = 7.8 Hz, H-1'), 4.52 (d, 1H, *J* = 11.1 Hz, CH*H* Bn), 4.59 (d, 1H, *J* = 11.1 Hz, CH*H* Bn), 4.68 (d, 1H, *J* = 11.1 Hz, CH*H* Bn), 4.73–4.79 (m, 4H, 2 × CH_2_ Bn), 4.84 (d, 1H, *J* = 11.0 Hz, CH*H* Bn), 4.89–4.94 (m, 4H, 2 × CH_2_ Bn), 7.18–7.43 (m, 36H, H_arom_), 7.69–7.72 (m, 4H, H_arom_); ^13^C-NMR: δ = 19.2 (C_q_ *t*Bu), 23.8, 24.0, 25.1 (3 × CH_2_ cyclohexylidene), 26.8 (3 × CH_3_ *t*Bu), 34.8, 36.5 (2 × CH_2_ cyclohexylidene), 64.7 (C-6″), 66.5 (CH_2_ glycerol), 66.9 (C-6'), 68.9 (C-6), 70.4 (CH_2_ glycerol), 71.9 (C-4″), 72.4 (C-2″), 73.8 (CH glycerol), 74.2 (C-5’), 74.8 (CH_2_ Bn), 74.8 (C-5″), 74.8 (CH_2_ Bn), 74.9 (C-5), 75.0, 75.0, 75.7, 75.7 (4 × CH_2_ Bn), 76.1 (C-3″), 77.9 (C-4'), 78.0 (C-4), 82.0 (C-2'), 82.1 (C-2), 84.6, 84.6 (C-3, C-3'), 102.9 (C-1″), 103.6 (C-1), 103.9 (C-1'), 110.0 (C_q_ cyclohexylidene), 127.6–128.4 (CH_arom_), 129.8, 129.8 (CH_arom_), 132.8 (C_q_ Ph), 132.9 (C_q_ Ph), 135.6, 135.6 (CH_arom_), 137.9, 137.9, 138.2, 138.3, 138.4, 138.5 (6 × C_q_ Bn); HRMS: C_85_H_100_O_18_Si + Na^+^ requires 1459.6571, found 1459.6587.

Intermediate E 


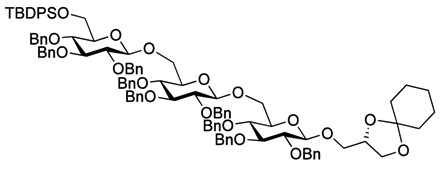


To a cooled (0 °C) solution of intermediate D (1.64 g, 1.14 mmol) in DMF (23 mL) were added benzyl bromide (0.814 mL, 1.17 g, 6.85 mmol) and sodium hydride (40% dispersion in mineral oil, 274 mg, 6.84 mmol), respectively. The mixture was stirred for 2 h, while slowly warming to room temperature, before methanol (2.0 mL) was added. After stirring for 15 min, water (50 mL) was added and the mixture extracted with Et_2_O (100 mL). The organic layer was washed with water (50 mL) and brine (50 mL) after which it was dried (Na_2_SO_4_), filtered, and concentrated *in vacuo*. Purification of the residue by column chromatography (Et_2_O/PE, 0/1 → 1/1) gave intermediate E (1.61 g, 0.943 mmol, 83%) as a colourless oil. 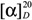 (CHCl_3_): +9.6; ^1^H-NMR: δ = 1.06 (s, 9H, *t*-Bu), 1.28–1.41 (m, 2H, CH_2_ cyclohexylidene), 1.50–1.61 (m, 8H, 4 × CH_2_ cyclohexylidene), 3.27–3.31 (m, 2H, H-4, H-5″), 3.36–3.41 (m, 2H, H-2', CH*H* glycerol), 3.45–3.49 (m, 2H, H-2″, H-5), 3.52–3.73 (m, 9H, H-2, H-3, H-3', H-3″, H-4, H-5', H-6, H-6', CH*H* glycerol), 3.78 (t, 1H, *J* = 9.3 Hz, H-4″), 3.89–3.94 (m, 4H, 2 × H-6″, 2 × C*H*H glycerol), 4.14–4.20 (m, 2H, H-6, CH glycerol), 4.25–4.29 (m, 1H, H-6'), 4.28 (d, 1H, *J* = 7.8 Hz, H-1'), 4.36 (d, 1H, *J* = 10.9 Hz, CH*H* Bn), 4.41 (d, 1H, *J* = 7.7 Hz, H-1), 4.56 (d, 1H, *J* = 11.4 Hz, CH*H* Bn), 4.59 (d, 1H, *J* = 7.9 Hz, H-1″), 4.63–4.81 (m, 9H, 4 × CH_2_ Bn, CH*H* Bn), 4.87–4.97 (m, 6H, 3 × CH_2_ Bn), 5.06 (d, 1H, *J* = 11.2 Hz, CH*H* Bn), 7.12-7.42 (m, 51H, H_arom_), 7.69–7.73 (m, 2H, H_arom_), 7.75–7.80 (m, 2H, H_arom_); ^13^C-NMR: δ = 19.3 (C_q_ *t*Bu), 23.8, 24.0, 25.1 (3 × CH_2_ cyclohexylidene), 26.8 (3 × CH_3_ *t*Bu), 34.9, 36.4 (2 × CH_2_ cyclohexylidene), 62.6 (C-6″), 66.3 (CH_2_ glycerol), 68.4, 68.4 (C-6, C-6'), 70.2 (CH_2_ glycerol), 73.9 (CH glycerol), 74.6 (C-5'), 74.6, 74.7, 74.8, 74.8, 74.9, 75.1 (6 × CH_2_ Bn), 75.4 (C-5″), 75.5, 75.7 (2 × CH_2_ Bn), 75.7 (C-5), 75.8 (CH_2_ Bn), 77.6 (C-4″), 78.0 (C-4), 78.2 (C-4'), 82.0 (C-2'), 82.2 (C-2″), 82.3 (C-2), 84.5, 84.8, 84.9 (C-3, C-3', C-3″), 103.5 (C-1'), 104.1 (C-1, C-1″), 109.9 (C_q_ cyclohexylidene), 127.5–128.4 (CH_arom_), 129.6, 129.6 (CH_arom_), 133.1 (C_q_ Ph), 133.7 (C_q_ Ph), 135.5, 135.9 (CH_arom_), 137.9–138.6 (9 × C_q_ Bn); HRMS: C_106_H_118_O_18_Si + Na^+^ requires 1729.7980, found 1729.7984.

Intermediate F 


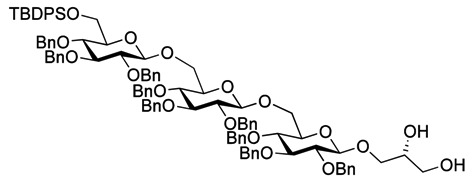


A solution of intermediate E (1.46 g, 0.855 mmol) in a mixture of acetic acid (34 mL), toluene (8.6 mL) and water (8.6 mL) was stirred for 3 h at 90 °C. After cooling down to rt the mixture was diluted with EtOAc (250 mL) and washed with water (2 × 100 mL), sat. aq. NaHCO_3_ (2 × 100 mL) and brine (100 mL). The organic layer was dried with Na_2_SO_4_, filtered and concentrated *in vacuo*, after which the residue was purified by column chromatography (Et_2_O/PE, 1/9 → 2/1) giving semiprotected intermediate F (1.20 g, 0.737 mmol, 86%) as a colourless oil. 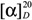 (CHCl_3_): +8.1; ^1^H-NMR: δ = 1.06 (s, 9H, *t*-Bu), 3.31–3.79 (m, 19H, H-2, H-2', H-2″, H-3, H-3', H-3″, H-4, H-4', H-4″, H-5, H-5', H-5″, H-6, H-6', 2 × CH_2_ glycerol, CH glycerol), 3.92–3.94 (m, 2H, 2 × H-6″), 4.16 (dd, 1H, *J* = 1.6 Hz, 11.6 Hz, H-6), 4.24–4.27 (m, 1H, H-6'), 4.29 (d, 1H, *J* = 7.8 Hz, H-1'), 4.36 (d, 1H, *J* = 10.9 Hz, CH*H* Bn), 4.47–4.51 (m, 2H, H-1, H-1″), 4.55 (d, 1H, *J* = 11.3 Hz, CH*H* Bn), 4.61 (d, 1H, *J* = 10.9 Hz, CH*H* Bn), 4.68–4.95 (m, 14H, 7 × CH_2_ Bn), 5.04 (d, 1H, *J* = 11.2 Hz, CH*H* Bn), 7.09–7.41 (m, 51H, H_arom_), 7.69–7.72 (m, 2H, H_arom_), 7.75–7.79 (m, 2H, H_arom_); ^13^C-NMR: δ = 19.3 (C_q_ *t*Bu), 26.8 (3 × CH_3_ *t*Bu), 62.7 (C-6″), 63.4 (CH_2_ glycerol), 68.2, 68.3 (C-6, C-6'), 70.7 (CH glycerol), 72.8 (CH_2_ glycerol), 74.8, 74.9, 74.9 (3 × CH_2_ Bn), 74.9, 75.0 (C-5, C-5'), 75.0, 75.0, 75.2 (3 × CH_2_ Bn), 75.5 (C-5″), 75.6, 75.6, 75.8 (3 × CH_2_ Bn), 77.7, 78.0, 78.3 (C-4, C-4', C-4″), 81.9, 82.1, 82.5 (C-2, C-2', C-2″), 84.5, 84.6, 84.7 (C-3, C-3', C-3″), 103.7, 104.0, 104.0 (C-1, C-1', C-1″), 127.5–128.4 (CH_arom_), 129.6, 129.6 (CH_arom_), 133.1 (C_q_ Ph), 133.6 (C_q_ Ph), 135.5, 135.9 (CH_arom_), 137.9–138.6 (9 × C_q_ Bn); HRMS: C_100_H_110_O_18_Si + Na^+^ requires 1649.7354, found 1649.7360.

Intermediate G


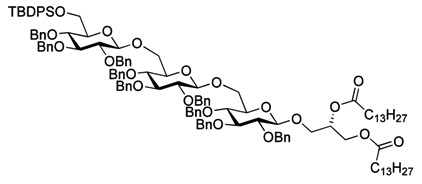


To a solution of intermediate F (260 mg, 0.160 mmol) and pyridine (0.129 mL, 127 mg, 1.60 mmol) in DCM (3.2 mL) were added myristoyl chloride (0.130 mL, 118 mg, 0.478 mmol) and a catalytic amount of 4-DMAP, respectively. After stirring for 3 h, methanol (1.0 mL) was added and after 10 min of stirring the volatiles were removed under reduced pressure. The residue was taken up in EtOAc (25 mL) and washed with aqueous 1 M HCl (10 mL), sat. aq. NaHCO_3_ (10 mL) and brine (10 mL). The organic layer was dried (Na_2_SO_4_), filtered, and concentrated *in vacuo*. The residue was purified by column chromatography (Et_2_O/PE/Et_3_N, 0/1/0.01 → 1/1/0.02), giving intermediate G (293 mg, 0.143 mmol, 90%) as a colourless oil. 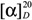 (CHCl_3_): +10.7; ^1^H-NMR: δ = 0.88 (t, 6H, *J* = 6.9 Hz, 2 × CH_3_ myristoyl), 1.04 (s, 9H, *t*-Bu), 1.19–1.29 (m, 40H, 20 × CH_2_ myristoyl), 1.51–1.58 (m, 4H, 2 × CH_2_ myristoyl), 2.18–2.26 (m, 4H, 2 × CH_2_ myristoyl), 3.25–3.68 (m, 11H, H-2, H-2', H-2″, H-3, H-3', H-4, H-4', H-5, H-5', H-5″, CH*H* glycerol), 3.63–3.68 (m, 3H, H-3″, H-6, H-6'), 3.76 (t, 1H, *J* = 9.3 Hz, H-4″), 3.90–3.93 (m, 3H, C*H*H glycerol, 2 × H-6″), 4.11 (dd, 1H, *J* = 7.3 Hz, 11.9 Hz, CH*H* glycerol), 4.16 (m, 1H, H-6), 4.20–4.27 (m, 3H, H-1', H-6', C*H*H glycerol), 4.36 (d, 1H, *J* = 11.0 Hz, CH*H* Bn), 4.39 (d, 1H, *J* = 7.7 Hz, H-1), 4.51 (d, 1H, *J* = 7.8 Hz, H-1″), 4.54 (d, 1H, *J* = 11.4 Hz, CH*H* Bn), 4.60–4.79 (m, 9H, 4 × CH_2_ Bn, CH*H* Bn), 4.85–4.96 (m, 6H, 3 × CH_2_ Bn), 5.04 (d, 1H, *J* = 11.2 Hz, CH*H* Bn), 5.12-5.15 (m, 1H, CH glycerol), 7.11–7.41 (m, 51H, H_arom_), 7.69–7.71 (m, 2H, H_arom_), 7.74–7.77 (m, 2H, H_arom_); ^13^C-NMR: δ = 14.1 (2 × CH_3_ myristoyl), 19.3 (C_q_ *t*Bu), 22.7 (2 × CH_2_ myristoyl), 24.9, 24.9 (2 × CH_2_ myristoyl), 26.8 (3 × CH_3_ *t*Bu), 29.1–29.7 (16 × CH_2_ myristoyl), 31.9 (2 × CH_2_ myristoyl, 34.1, 34.3 (2 × CH_2_ myristoyl), 62.6 (C-6″), 62.8 (CH_2_ glycerol), 68.0 (CH_2_ glycerol) 68.4, 68.5 (C-6, C-6'), 69.8 (CH glycerol), 74.6 (CH_2_ Bn), 74.6 (C-5'), 74.7, 74.8, 74.9, 74.9, 75.1 (5 × CH_2_ Bn), 75.2 (C-5), 75.5, 75.6 (2 × CH_2_ Bn), 75.7 (C-5″), 75.8 (CH_2_ Bn), 77.6 (C-4″), 78.0, 78.1 (C-4, C-4'), 81.8, 82.1, 82.3 (C-2, C-2', C-2″), 84.4, 84.8, 84.8 (C-3, C-3', C-3″), 103.6, 104.1, 104.2 (C-1, C-1', C-1″), 127.5–128.4 (CH_arom_), 129.6, 129.6 (CH_arom_), 133.1 (C_q_ Ph), 133.6 (C_q_ Ph), 135.5, 135.9 (CH_arom_), 138.0–138.6 (9 × C_q_ Bn), 172.9, 173.3 (2 × C_q_ myristoyl); HRMS: C_128_H_162_O_20_Si + Na^+^ requires 2071.1354, found 2071.1370.

*Gentiotriosyl Derivative* **14**


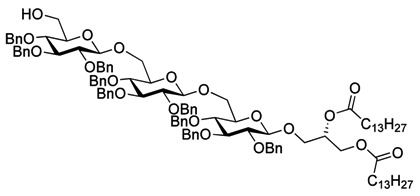


To a solution of intermediate G (274 mg, 0.134 mmol) in THF (2.7 mL) was added TBAF (1.0 M solution in THF, 0.40 mL, 0.40 mmol). After stirring overnight at rt, the volatiles were removed under reduced pressure after which the residue was purified by column chromatography (EtOAc/PE, 1/9 → 1/2). Semiprotected gentiotriosyl glycolipid **14** (205 mg, 0.113 mmol, 85%) was obtained as a colourless oil. 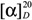 (CHCl_3_): +12.5; ^1^H-NMR: δ = 0.89 (t, 6H, *J* = 7.0 Hz, 2 × CH_3_ myristoyl), 1.19–1.34 (m, 40H, 20 × CH_2_ myristoyl), 1.51–1.59 (m, 4H, 2 × CH_2_ myristoyl), 2.17–2.25 (m, 4H, 2 × CH_2_ myristoyl), 3.26–3.69 (m, 15H, H-2, H-2', H-2″, H-3, H-3', H-3″, H-4, H-4', H-4″, H-5, H-5', H-5″, H-6, H-6″, CH*H* glycerol), 3.74 (dd, 1H *J* = 5.2 Hz, 11.1 Hz, H-6'), 3.83 (dd, 1H, *J* = 2.6 Hz, 11.9 Hz, H-6″), 3.92 (dd, 1H, *J* = 4.6 Hz, 10.9 Hz, CH*H* glycerol), 4.06–4.15 (m, 3H, H-6, H-6', CH*H* glycerol), 4.23–4.27 (m, 2H, H-1, C*H*H glycerol), 4.39–4.44 (m, 2H, H-1', CH*H* Bn), 4.46 (d, 1H, *J* = 7.8 Hz, H-1″), 4.55 (d, 1H, *J* = 11.2 Hz, CH*H* Bn), 4.62–4.68 (m, 3H, CH_2_ Bn, CH*H* Bn), 4.71–4.79 (m, 6H, 3 × CH_2_ Bn), 4.84 (d, 1H, *J* = 11.0 Hz, CH*H* Bn), 4.87–4.96 (m, 6H, 3 × CH_2_ Bn), 5.13–5.17 (m, 1H, CH glycerol), 7.16–7.36 (m, 45H, H_arom_); ^13^C-NMR: δ = 14.1 (2 × CH_3_ myristoyl), 22.7 (2 × CH_2_ myristoyl), 24.9, 24.9 (2 × CH_2_ myristoyl), 29.1–29.7 (16 × CH_2_ myristoyl), 31.9 (2 × CH_2_ myristoyl, 34.1, 34.3 (2 × CH_2_ myristoyl), 62.0 (C-6″), 62.8 CH_2_ glycerol), 68.0 (CH_2_ glycerol), 68.7 (C-6), 69.3 (C-6'), 69.8 (CH glycerol), 74.6 (C-5'), 74.6, 74.8, 74.8, 74.9, 74.9 (5 × CH_2_ Bn), 75.0 (C-5), 75.0 (CH_2_ Bn), 75.2 (C-5″), 75.6, 75.6, 75.7 (3 × CH_2_ Bn), 77.6, 78.0, 78.0 (C-4, C-4', C-4″), 81.8, 82.1, 82.1 (C-2, C-2', C-2″), 84.5, 84.5, 84.8 (C-3, C-3', C-3″), 103.5, 104.0, 104.2 (C-1, C-1', C-1″), 127.5–128.4 (CH_arom_), 137.9–138.5 (9 × C_q_ Bn), 172.9, 173.3 (2 × C_q_ myristoyl); HRMS: C_112_H_144_O_20_ + Na^+^ requires 1832.0143, found 1832.0149.

*Glycolipid* **15**


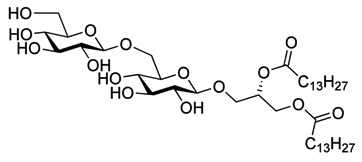


A solution of compound **7** (16.9 mg, 12.3 µmol) in a mixture of 1,4-dioxane/water/acetic acid (5/1/0.05, 2.0 mL) was purged with argon. Palladium black (~5 mg, ~50 µmol) was added and the reaction was stirred under hydrogen atmosphere for 2 days. The mixture was filtered over Celite and concentrated under reduced pressure. Purification of the residue by reversed phase column chromatography (C_8_ silica, H_2_O/iPrOH, 4/1 → 1/9) gave glycolipid **15** (4.9 mg, 5.9 µmol, 48%) as an amorphous white solid. ^1^H-NMR (CD_3_OD): δ = 0.90 (t, 6H, *J* = 7.0 Hz, 2 × CH_3_ myristoyl), 1.26–1.38 (m, 40H, 20 × CH_2_ myristoyl), 1.57–1.64 (m, 4H, 2 × CH_2_ myristoyl), 2.31–2.35 (m, 4H, 2 × CH_2_ myristoyl), 3.16–3.20 (m, 1H, H-2), 3.22 (dd, 1H, *J* = 7.9 Hz, 9.2 Hz, H-2'), 3.26–3.30 (m, 2H, H-4', H-5'), 3.32–3.37 (m, 3H, H-3, H-3', H-4), 3.44–3.48 (m, 1H, H-5), 3.67 (dd, 1H, *J* = 5.4 Hz, 11.8 Hz, H-6'), 3.74–3.78 (m, 2H, H-6, CH*H* glycerol), 3.87 (dd, 1H, *J* = 1.8 Hz, 11.9 Hz, H-6'), 3.99 (dd, 1H, *J* = 5.5 Hz, 11.0 Hz, C*H*H glycerol), 4.16 (dd, 1H, *J* = 2.0 Hz, 11.5 Hz, H-6), 4.22 (dd, 1H, *J* = 6.9 Hz, 12.1 Hz, CH*H* glycerol), 4.28 (d, 1H, *J* = 7.8 Hz, H-1), 4.37 (d, 1H, *J* = 7.8 Hz, H-1'), 4.43 (dd, 1H, *J* = 3.0 Hz, 12.1 Hz, C*H*H glycerol), 5.26–5.30 (m, 1H, CH glycerol); ^13^C-NMR (CD_3_OD): δ = 14.5 (2 × CH_3_ myristoyl), 23.8 (2 × CH_2_ myristoyl), 26.1, 26.1 (2 × CH_2_ myristoyl), 30.2–30.8 (16 × CH_2_ myristoyl), 33.1 (2 × CH_2_ myristoyl, 35.0, 35.2 (2 × CH_2_ myristoyl), 62.8 (C-6'), 64.0 CH_2_ glycerol), 68.9 (CH_2_ glycerol), 70.0 (C-6), 71.5 (C-4), 71.6 (C-4'), 71.8 (CH glycerol), 74.9 (C-2), 75.1 (C-2'), 77.1 (C-5), 77.8 (C-5'), 78.0, 78.0 (C-3, C-3'), 104.7 (C-1), 104.9 (C-1'), 174.9, 175.1 (2 × C_q_ myristoyl); HRMS: C_43_H_80_O_15_ + Na^+^ requires 859.5389, found 859.5378.

*Glycolipid* **16**


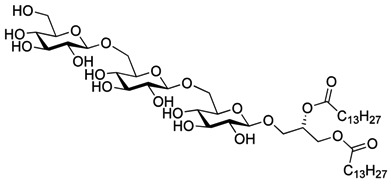


A solution of gentiotriosyl derivative **14** (46.8 mg, 25.9 µmol) in a mixture of THF/water/AcOH (5/1/0.05, 5.6 mL) was purged with argon. Palladium black (~5 mg, ~50 µmol) was added and the reaction was stirred under hydrogen atmosphere (1 bar) for 2 days. The mixture was filtered over Celite and concentrated under reduced pressure. Purification of the residue by reversed phase column chromatography (C_8_ silica, H_2_O/iPrOH, 4/1 → 1/9) gave glycolipid **16** (21.0 mg, 21.0 µmol, 81%) as an amorphous white solid. ^1^H-NMR (CD_3_OD): δ = 0.90 (t, 6H, *J* = 7.0 Hz, 2 × CH_3_ myristoyl), 1.26–1.37 (m, 40H, 20 × CH_2_ myristoyl), 1.56–1.65 (m, 4H, 2 × CH_2_ myristoyl), 2.30–2.36 (m, 4H, 2 × CH_2_ myristoyl), 3.17–3.38 (m, 10H, H-2, H-2', H-2″, H-3, H-3', H-3″, H-4, H-4', H-4″, H-5″), 3.45-3.52 (m, 2H, H-5, H-5'), 3.68 (dd, 1H, *J* = 5.3 Hz, 11.9 Hz, H-6″), 3.74–3.80 (m, 3H, H-6, H-6', CH*H* glycerol), 3.87 (dd, 1H, *J* = 1.9 Hz, 11.9 Hz, H-6″), 4.00 (dd, 1H, *J* = 5.5 Hz, 11.0 Hz, C*H*H glycerol), 4.13–4.18 (m, 2H, H-6, H-6'), 4.23 (dd, 1H, *J* = 7.0 Hz, 12.1 Hz, CH*H* glycerol), 4.30 (d, 1H, *J* = 7.8 Hz, H-1), 4.36–4.38 (m, 2H, H-1', H-1″), 4.44 (dd, 1H, *J* = 2.9 Hz, 12.1 Hz, C*H*H glycerol), 5.26–5.31 (m, 1H, CH glycerol); ^13^C-NMR (CD_3_OD): δ = 14.5 (2 × CH_3_ myristoyl), 23.8 (2 × CH_2_ myristoyl), 26.1, 26.1 (2 × CH_2_ myristoyl), 30.2–30.8 (16 × CH_2_ myristoyl), 33.1 (2 × CH_2_ myristoyl, 35.0, 35.2 (2 × CH_2_ myristoyl), 62.7 (C-6″), 64.1 CH_2_ glycerol), 68.9 (CH_2_ glycerol), 70.0, 70.6 (C-6, C-6'), 71.5, 71.6, 71.7, 71.8 (C-4, C-4', C-4″, CH glycerol), 74.9, 75.1, 75.1 (C-2, C-2', C-2″), 77.0, 77.0 (C-5, C-5'), 77.8, 77.9, 78.0, 78.0 (C-3, C-3', C-3″, C-5’″), 104.5, 104.9, 105.1 (C-1, C-1', C-1″), 174.9, 175.1 (2 × C_q_ myristoyl); HRMS: C_49_H_90_O_20_ + Na^+^ requires 1021.5918, found 1021.5899.

### 3.2. Physical Chemical Characterization

#### General

DMPC, DMPS and DOPE-Biotin (1,2-dioleoyl-*sn-*glycero-3-phosphoethanolamine-*N*-cap biotinyl) were purchased from Avanti Polar Lipids, Inc. (Alabaster, AL, USA) and used without further purification. The fluorescent dyes DiDC18 (1,1'-dioctadecyl-3,3,3',3'-tetramethylindodicarbocyanine-4-chlorobenzenesulfonate) and DiIC12 (1,1'-didodecyl-3,3,3',3'-tetramethylindodicarbocyanineperchlorate) were likewise used as received from Invitrogen (Paisley, UK). Unless stated otherwise, all other chemicals and reagents were purchased from Sigma-Aldrich A/S (Copenhagen, Denmark). Milli-Q quality water was used in all preparations.

### 3.3. Methods

Small Unilamellar Vesicles were prepared from hydrating lipid films using PBS buffer (10 mM phosphate buffer supplemented with 100 mM NaCl, pH 7.4) to a lipid concentration of 1 mg mL^−1^. The vesicles were extruded through 100 nm polycarbonate filters using the Mini-Extruder system (Avanti Polar Lipids, Inc.). This method provides a high degree of unilamellar structures and long-term stability what makes them suitable for calorimetric measurements [49]. To achieve an even distribution of the lipids the extruder was heated to 52 °C ensuring DMPC and DMPS in their molten state. Each sample ran through two cycles of up scans (10–70 °C) and down scans (70–10 °C) to evaluate whether there was any effect on the mixing behavior of the lipids triggered by the preparation method. These scans showed no hysteresis effects suggesting that the method of preparation did not have a significant effect on their thermotropic behavior.

Giant Unilamellar Vesicles were prepared by gentle hydration of lipid films according to previous protocols [42]. Briefly, lipids in chloroform were mixed in glass tubes to achieve the indicated lipid composition. The lipid dyes DiD C(18), DiI C(12) (Invitrogen) were added to a concentration of 0.5 mol% for fluorescence imaging. Additionally, the vesicles contained 0.5 mol% DOPE-Biotin (Avanti Polar Lipids, Inc.) to tether the vesicles on the surface of the microscope slides. Then, a thin lipid film was prepared by drop-by-drop addition of the mixture to small Teflon cups. The remaining chloroform was removed by storing the Teflon cups in a vacuum-chamber for 1 h. Lipids were rehydrated in D-sorbitol solution (46.1 g L^−1^ in PBS) to a lipid concentration of 0.2 mg mL^−1^. After one night of incubation at 50 °C all vesicles were stored at 4 °C and used within the same day.

Microscope chambers were functionalized by carefully cleaning glass coverslips in successive washes in Helmanex (2%), MilliQ water and ethanol. For surface functionalization, 1 g L^−1^ BSA–biotin:BSA (1:10, weight ratio) was added to the surface and incubated at ambient temperature for 10 min. After five times gentle washing with PBS, streptavidin (0.025 g L^−1^ in PBS) was added and likewise incubated for 10 min followed by five times washing with PBS. After surface functionalization, giant unilamellar vesicles were added to the microscopy chamber to a final constant lipid concentration of 0.01 g L^−1^ (~14 µM) and allowed to stabilize for at least 30 min before the measurement. The total volume of the microscope chamber was kept to ~200 µL.

Differential Scanning Calorimetry (DSC) was performed in a MicroCal^TM^ VP-DSC system (GE Healthcare Bio-Sciences AB, Uppsala, Sweden) and data were processed using Origin7 (Origin Lab, Northampton, MA, USA). Briefly, two up/down scan cycles were performed between 10 and 70 °C at a rate of 1 °C per 1.5 min, and duplicates were obtained for all samples studied. Prior to vesicle addition, the chambers were cleaned with plenty of 5% SDS solution followed by water, 1% pepsin solution, water and pure ethanol dried under vacuum. Then, 700 µL of the sample was filled in one chamber and buffer (PBS, 7.4 pH) as reference was filled in the other. The final lipid concentration was 1 mg/mL. Each sample ran through two scan cycles consisting of up scans (10–70 °C) and down scans (70–10 °C). The scan rate was set on 1 °C per 1.5 min. The experiments were taken out with the MicroCalTMVP-DSC system and the data processed with VPViewerTM2000 (GE Healthcare, Massachusetts, USA) as well as with Origin7 (OriginLab).

Microscopy imaging was performed on an inverted confocal microscope TCS SP5 (Leica, Wetzlar, Germany). In order to avoid crosstalk, DiD-C18 was excited with a laser and recorded using the filter 650/50 nm while DiI-C12 was excited using a laser and recorded using the filter at 480/50 nm. The signal was maximized setting the argon laser intensity to 19%, the acousto-Optic Tunable Filter for Attota to 21% and the PhotoMultiplier Voltage to 537.4 V. These settings were kept fixed during the various experiments in order to be able to perform fluorescence intensity comparison.

### 3.4. Fluorescence Microscopy Imaging

All glass slides were cleaned thoroughly due to sonication in Hellmannex for 1h. After sonication they were rinsed several times with water and ethanol. Additionally they were cleaned by etching and stored in 99% ethanol after. The glass slides were functionalyzed with 1 g/L BSA-Biotin: BSA (1:10) in PBS for 10 minutes. Five steps of washing with PBS followed before the slides were incubated with 0.25 g/L Streptavidin in PBS for another 10 min. After another five washing steps with PBS the GUVs were added to the surface with a final lipid concentration of 0.01 mg/L. The incubation time for the GUVs to settle to the glass amounted 30 min before they were analyzed. The samples were analyzed with the inverted Confocal Leica TCS SP5 and the images processed with the software Leica LAS AF (Leica Microsystems CMS, Mannheim, Germany).

## 4. Conclusions

The synthesis of glycoglycerolipids from ready available starting materials has been investigated and an improved procedure for the synthesis of gentiobiosyl glycolipids on larger scales has been investigated. The amount of side products formed in the glycosylation of cyclohexylidene protected glycerol have been limited by optimizing the reaction conditions. Conditions for the crystallization of the glycosylation product from the crude reaction mixture were developed and tedious chromatography can therefore be avoided allowing for up scaling. Prolonging the “head group” was performed by a selective deprotection of the 6'O-TBDPS group followed by glycosylation with benzoylated donor **12** giving the pseudotetrasaccharide in high yield and purity, with a 6″O-TBDPS protective group installed for further prolongation or attachment to a repeating unit via a phosphate linkage as it is found in LTA from *C. difficile*.

Our DSC/microscopy results suggest that glycolipids induce strong de-mixing in lipid mixtures and that the demixing ability depends on the conformation of the head-group as determined by the type of glycosidic bond and the number of sugar residues in the glycolipid headgroup. Indeed, glycolipids are believed to form lipid clusters for maximum efficiency of their biological roles that include participation in signaling pathways and in receptor recognition [50,51]. Our results suggest that the larger the number of sugars in the glycolipid headgroup, the stronger the demixing induced and that some preferential association occurs in which DMPC is excluded from the glycolipid rich regions domains.

## Supplementary Materials

Supplementary materials can be accessed at: http://www.mdpi.com/1420-3049/18/11/13546/s1.
