# Supplementary material for: Synthesis and Thermotropic Phase Behavior of Four Glycoglycerolipids
Source: Molecules. 2013 Nov 1;18(11):13546–73. doi: 10.3390/molecules181113546 (PMC6270285; doi:10.3390/molecules181113546)
Supplement: Supplementary file 1 [file molecules-18-13546-s001.pdf]

Supplementary Materials

Figure S1. Thermograms for pure 1) DMPS, 2) DMPC, 3) β-monoglycolipid (**18**) vesicles.

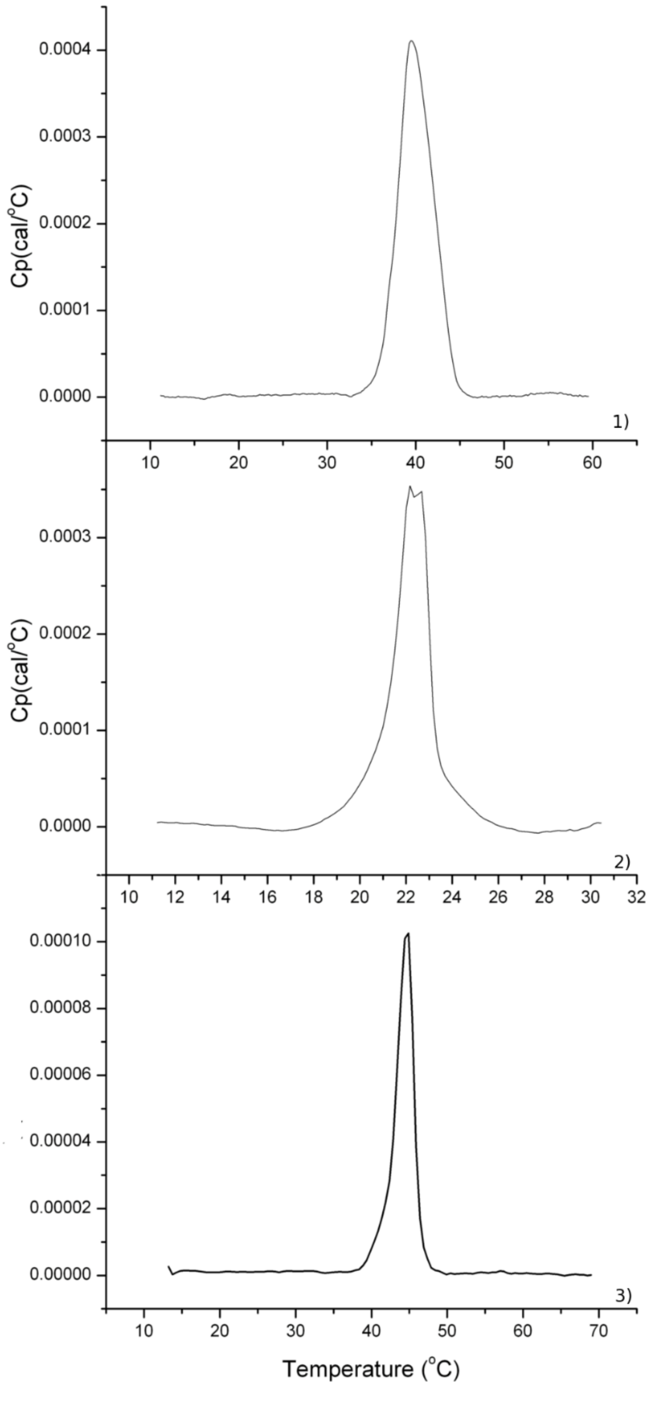

Figure S2. Differential Scanning Calorimetry (top) shows ideal mixing for DMPC and DMPS. Confocal scanning fluorescence microscopy (bottom) shows homogeneous distribution of two dyes occurs below (left) and above (right) the melting temperature. Vesicles composed of DMPC/DMPS for composition given in Table 1 labeled with 0.1% DiI C18 (green) and 0.1% DiIC18 (red) were prepared as described in the text. Color merged images were shown. Bar, 20 µm applies for both images.

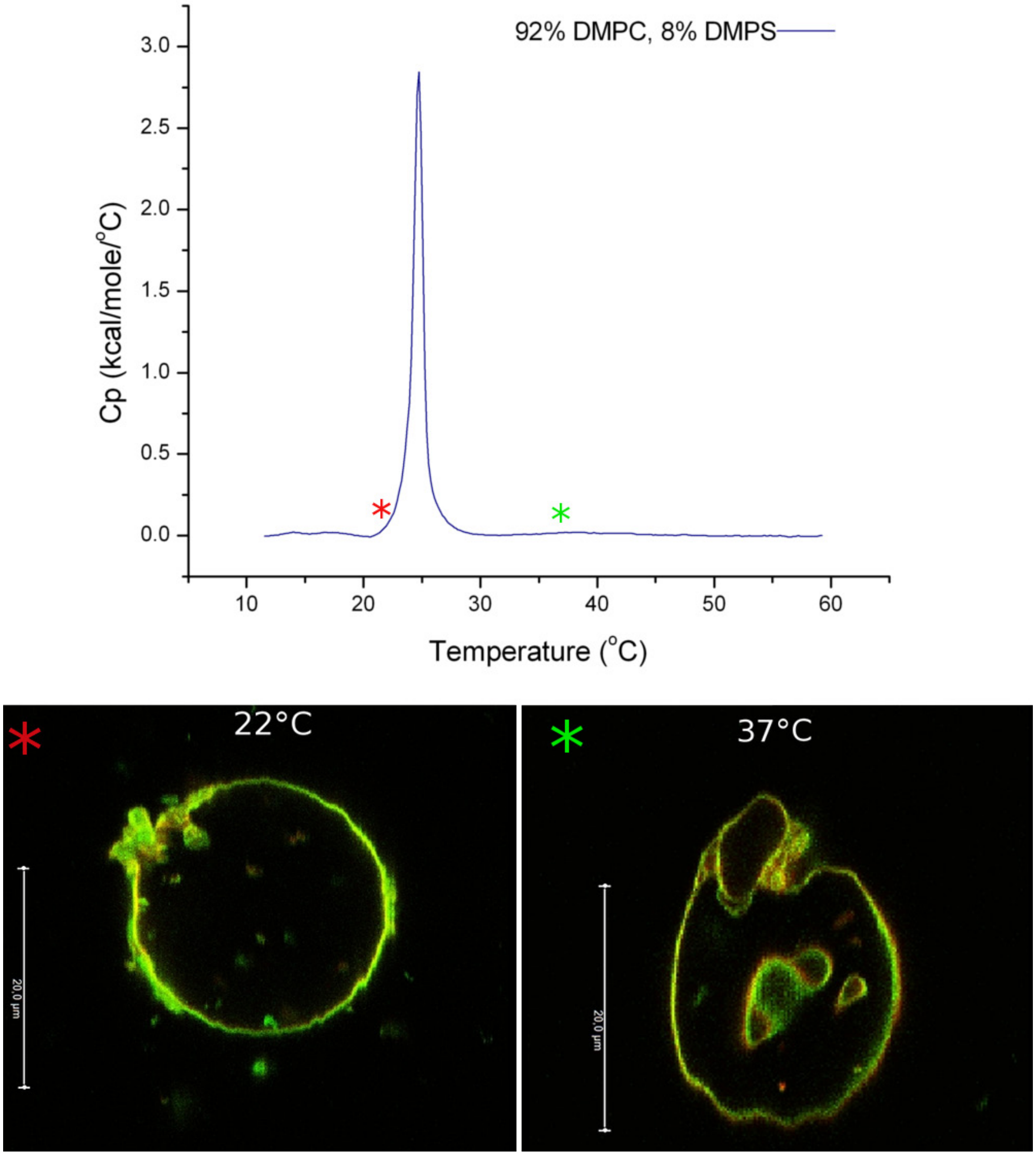

Figure S3. Chemical structures of the dyes used in the fluorescence imaging. The dye DiD C(18) was excited at 650/50 nm and the emission was collected with a filter to cut wavelength below 600 and above 800 nm. The dye DiI C(12) was excited at 480/50 nm and the emission was collected with a filter to cut wavelength below 600 nm and above 700 nm. These settings were chosen to avoid cross talk between the dyes.

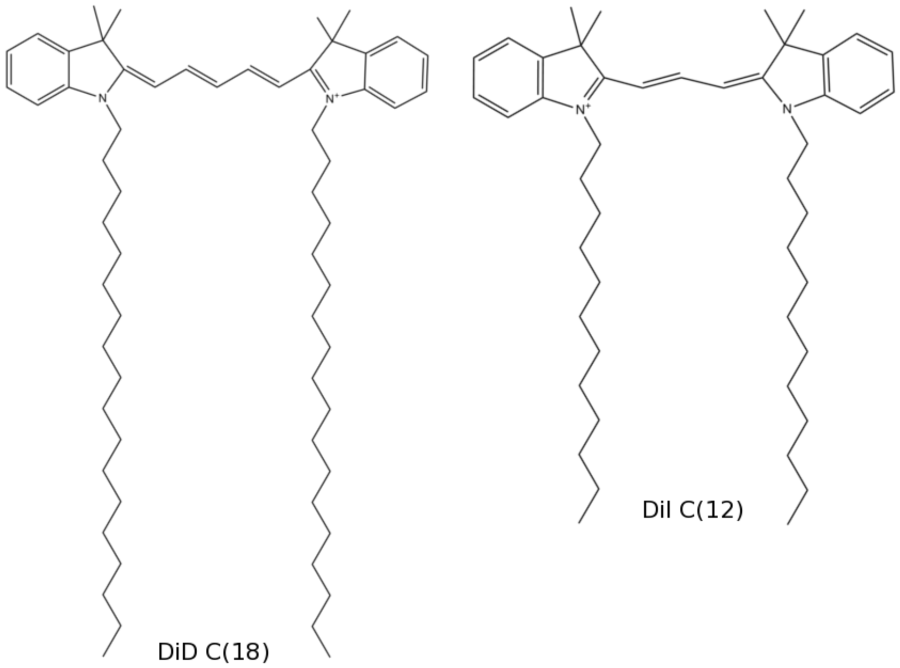

Compound **3**, ^{1}H NMR (500 MHz, $CDCl_3$)

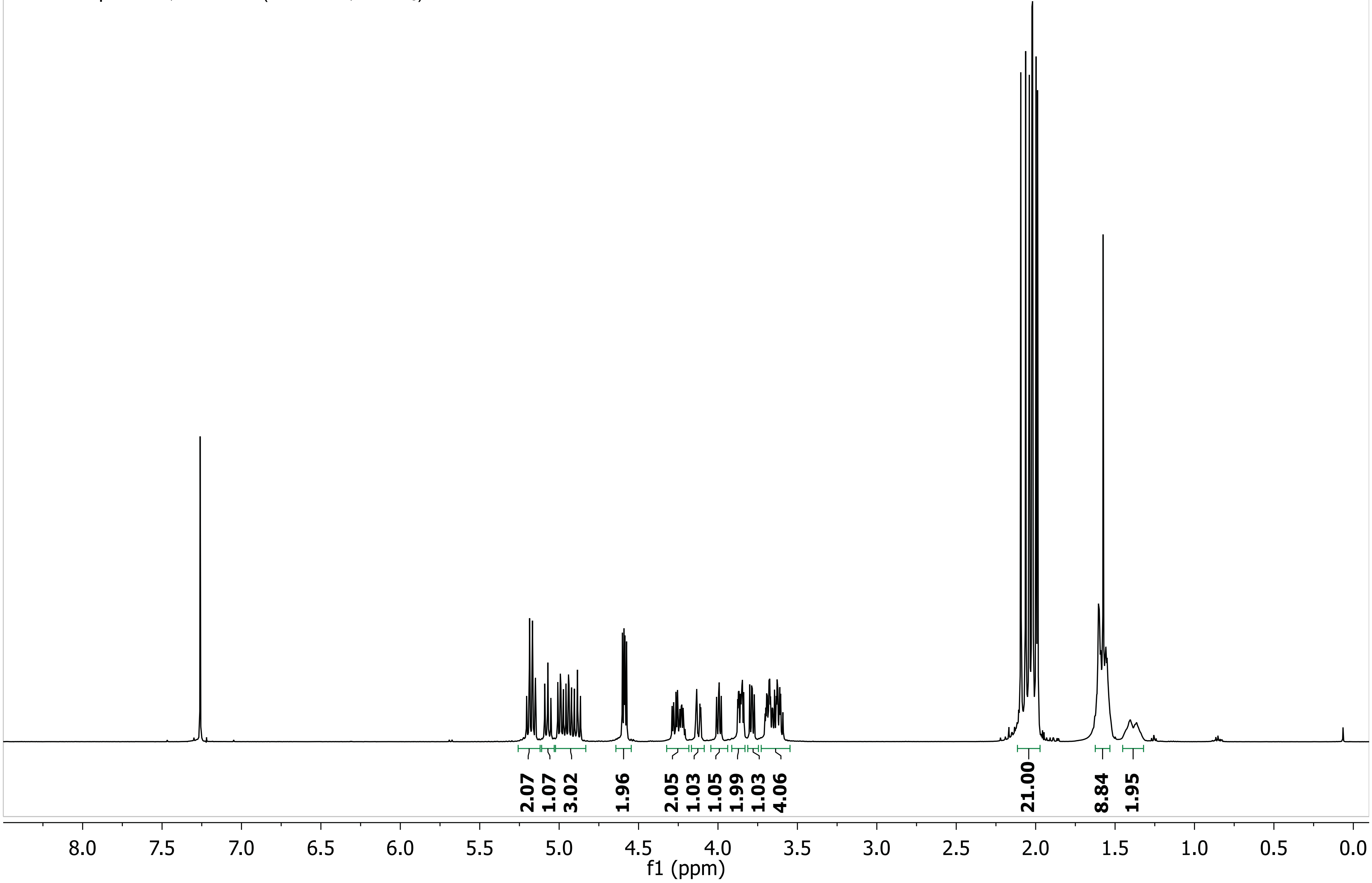

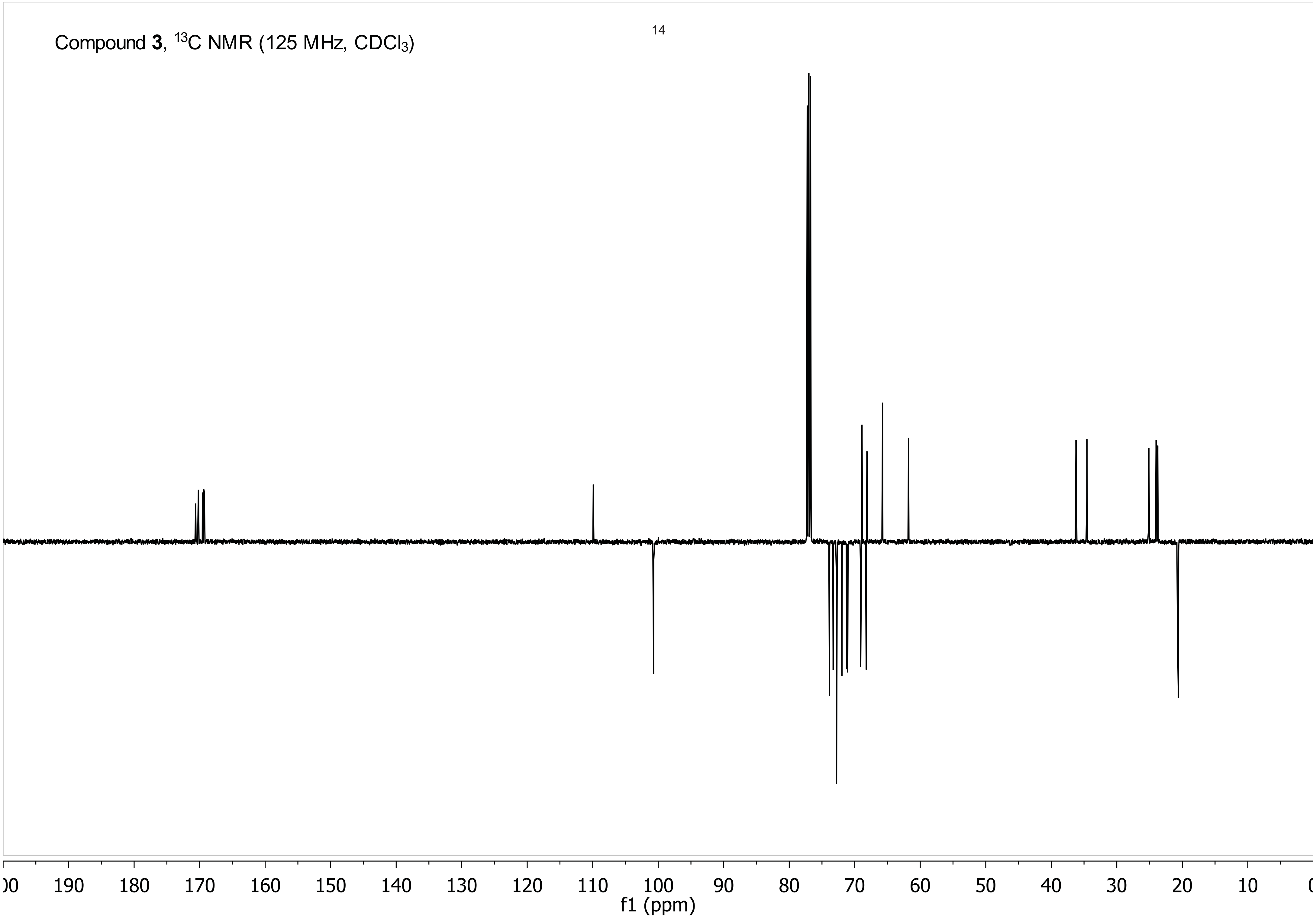
Compound 3, 13C NMR (125 MHz, CDCl3)
00 190 180 170 160 150 140 130 120 110 100 90 80 70 60 50 40 30 20 10 0
f1 (ppm)

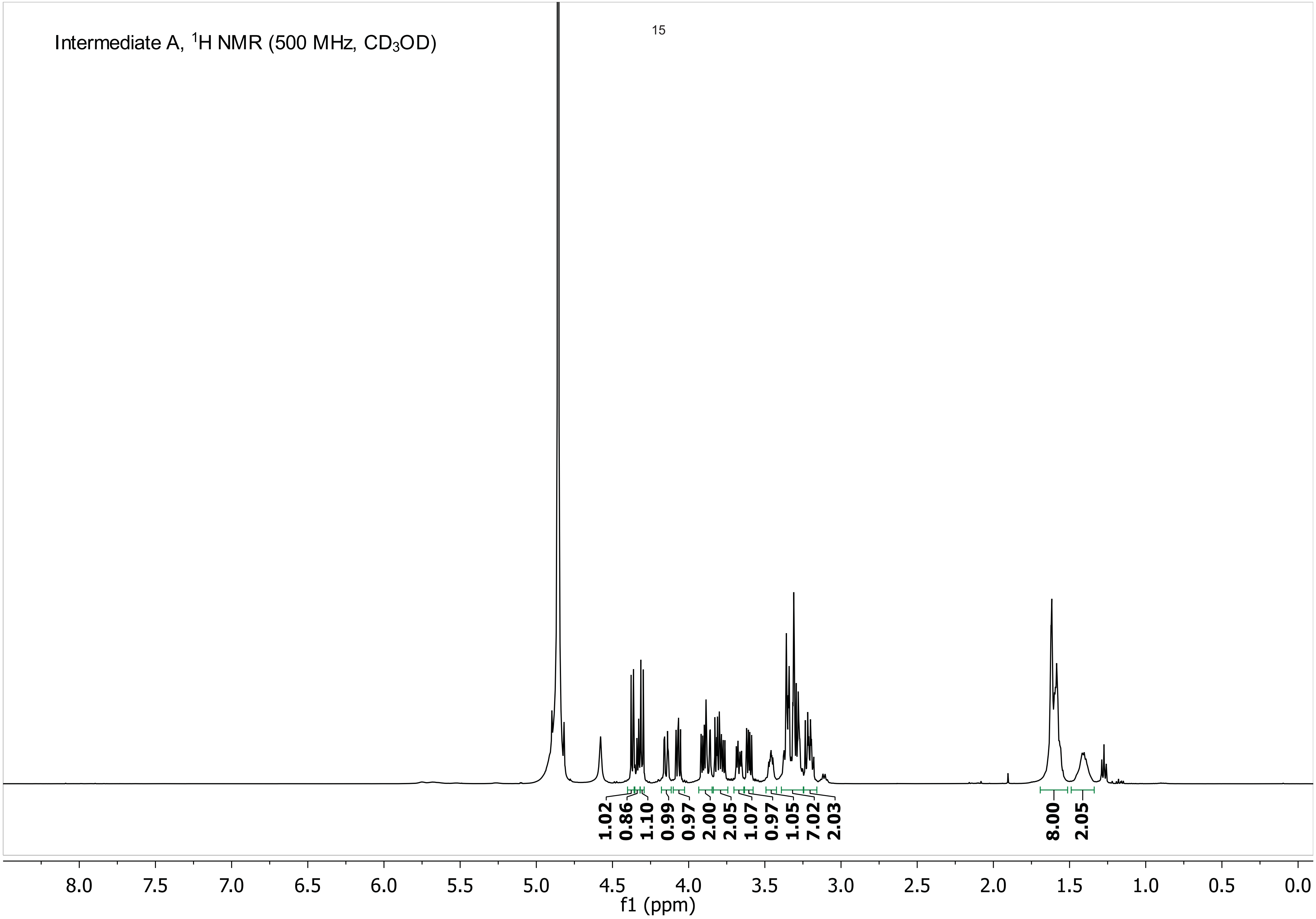
Intermediate A, ¹H NMR (500 MHz, CD3OD)
1.02
0.86
1.10
0.99
0.97
2.00
2.05
1.07
0.97
1.05
7.02
2.03
8.00
2.05
8.0 7.5 7.0 6.5 6.0 5.5 5.0 4.5 4.0 3.5 3.0 2.5 2.0 1.5 1.0 0.5 0.0
f1 (ppm)

Intermediate A, ^{13}C NMR (125 MHz, CD_3OD)

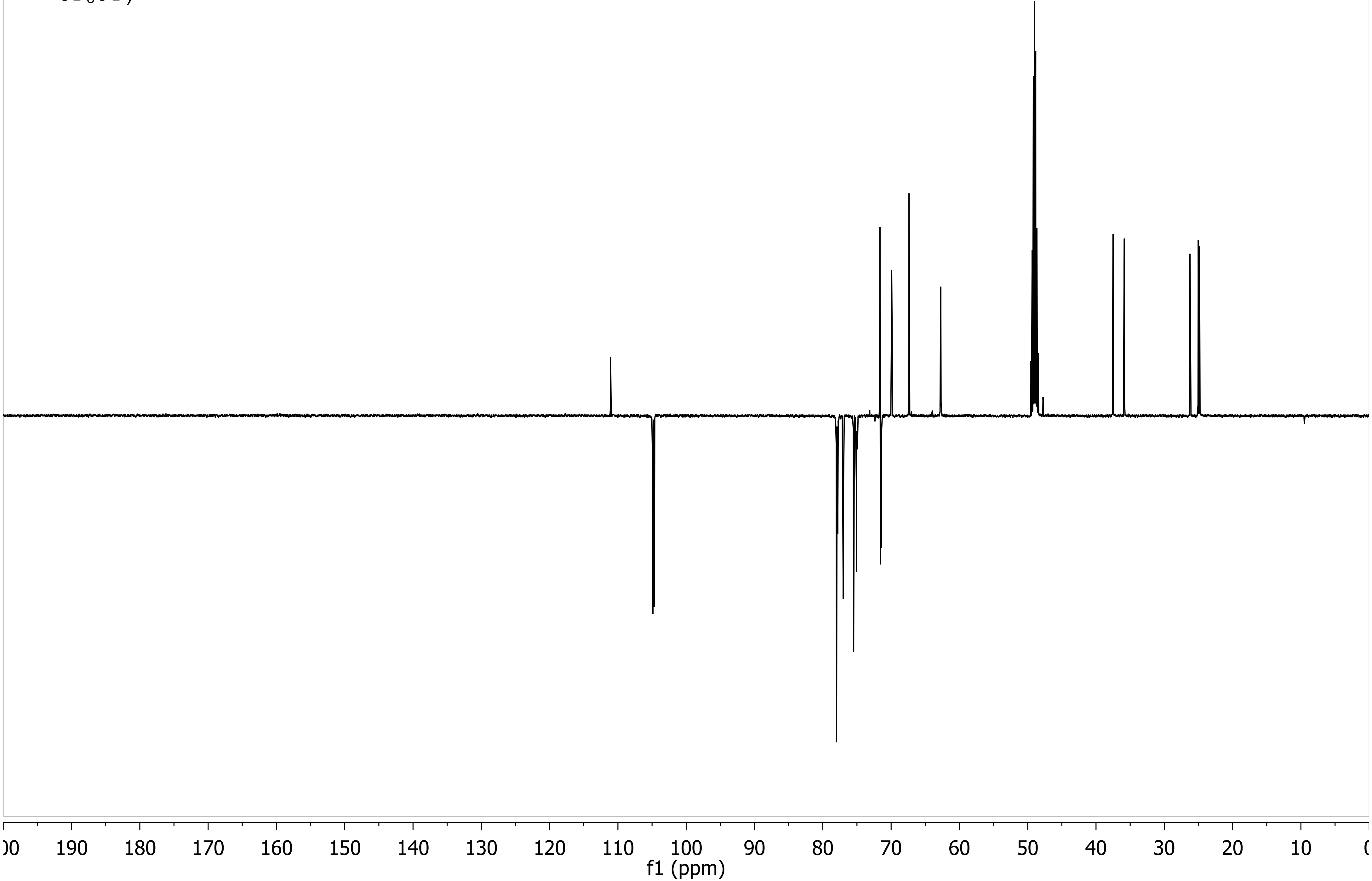

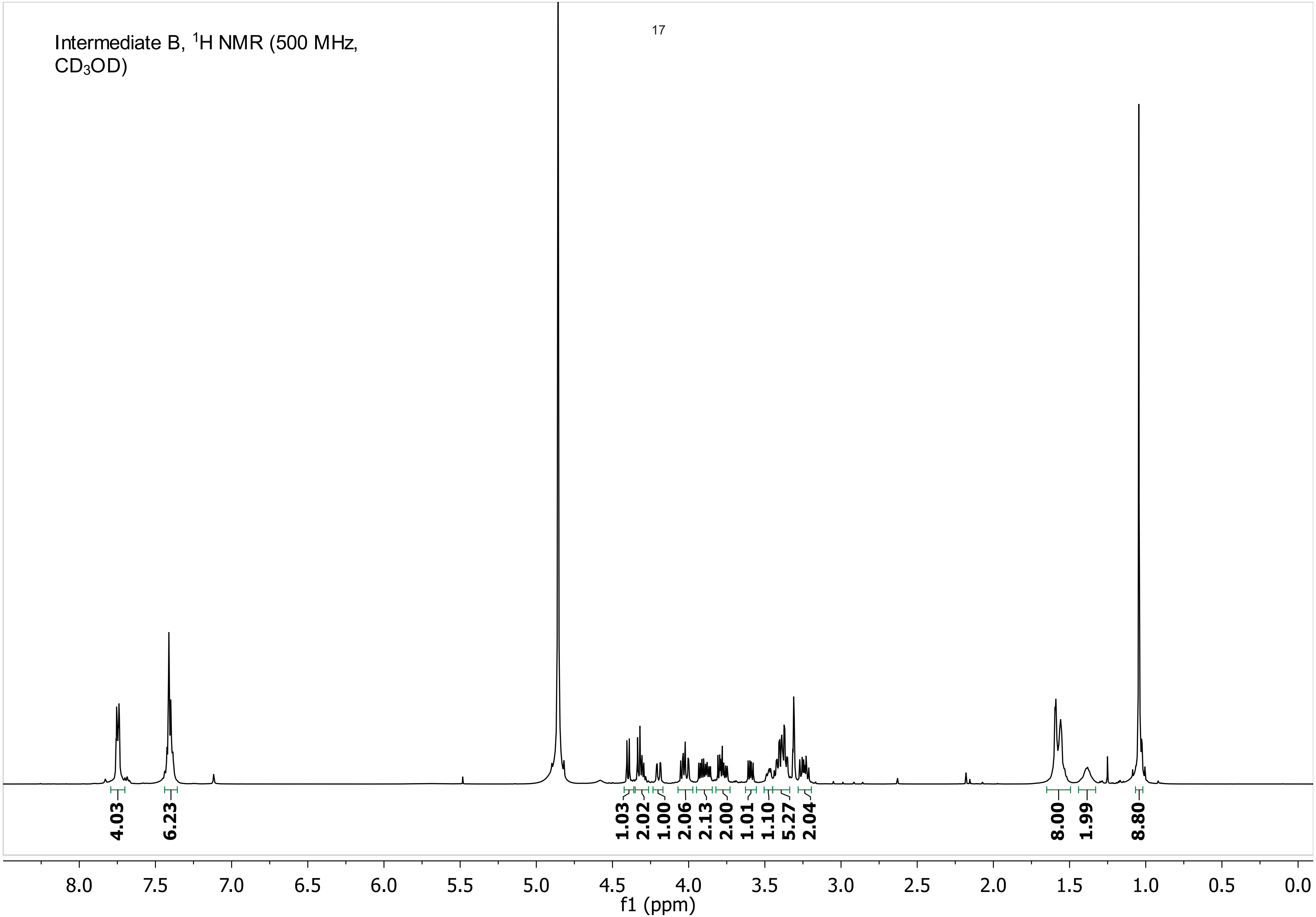

Intermediate B, 1H NMR (500 MHz, CD3OD)
4.03
6.23
1.03
2.02
1.00
2.06
2.13
2.00
1.01
1.10
5.27
2.04
8.00
1.99
8.80
8.0
7.5
7.0
6.5
6.0
5.5
5.0
4.5
4.0
3.5
3.0
2.5
2.0
1.5
1.0
0.5
0.0
f1 (ppm)

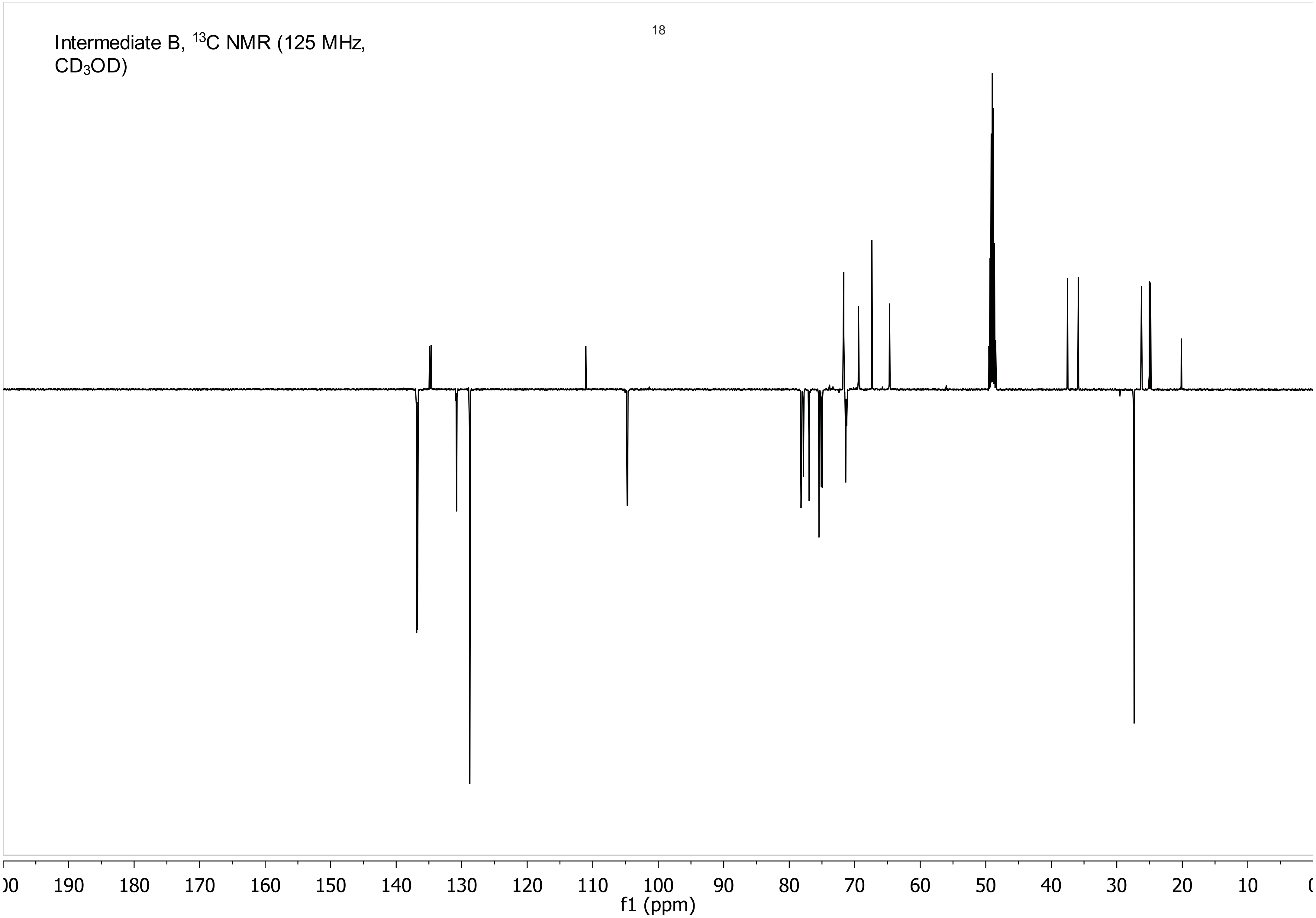
Intermediate B, ^{13}C NMR (125 MHz, CD_3OD)
190 180 170 160 150 140 130 120 110 100 90 80 70 60 50 40 30 20 10
f1 (ppm)

Compound **5**, ^{1}H NMR (500 MHz, $CDCl_3$)

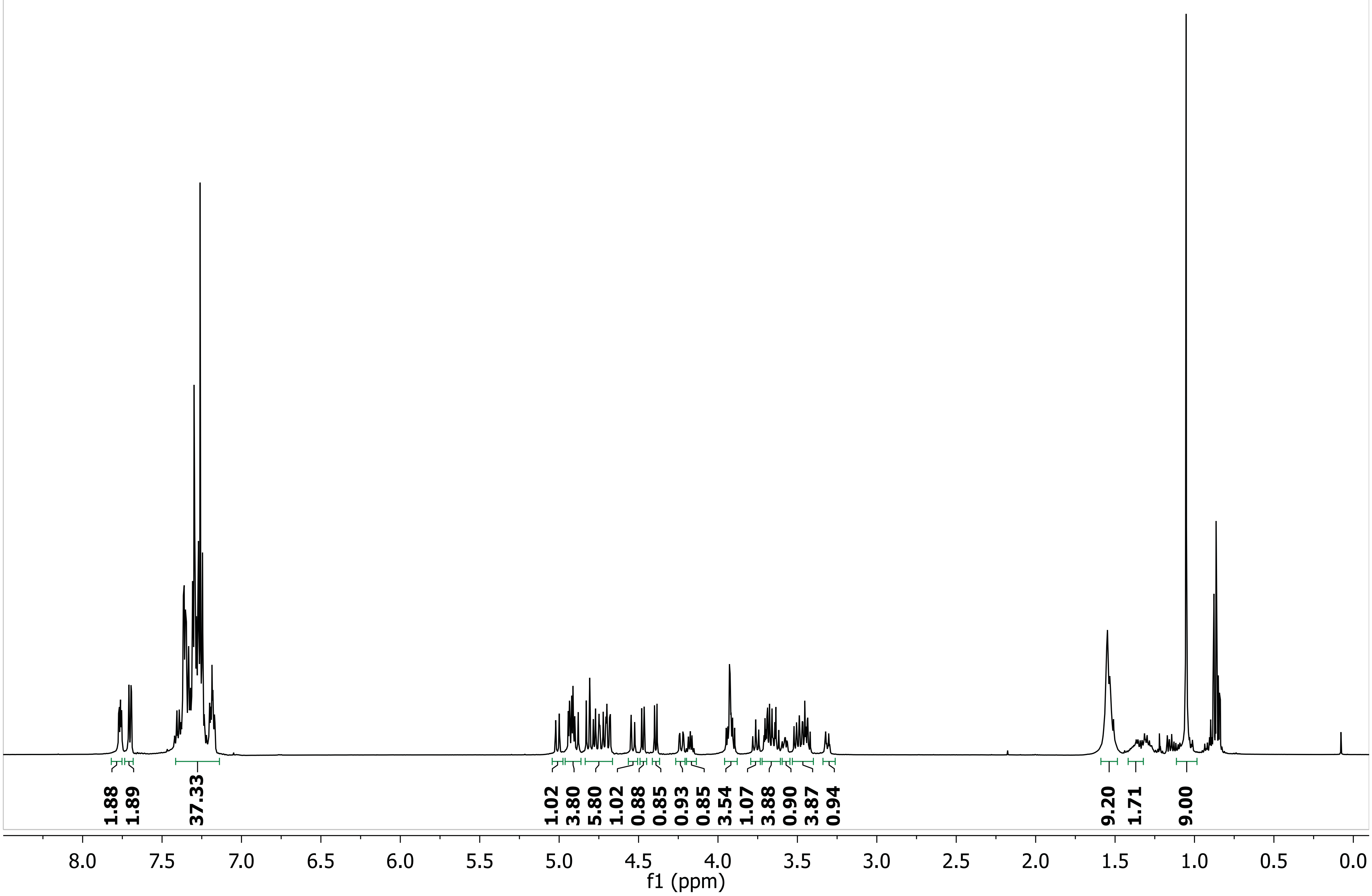

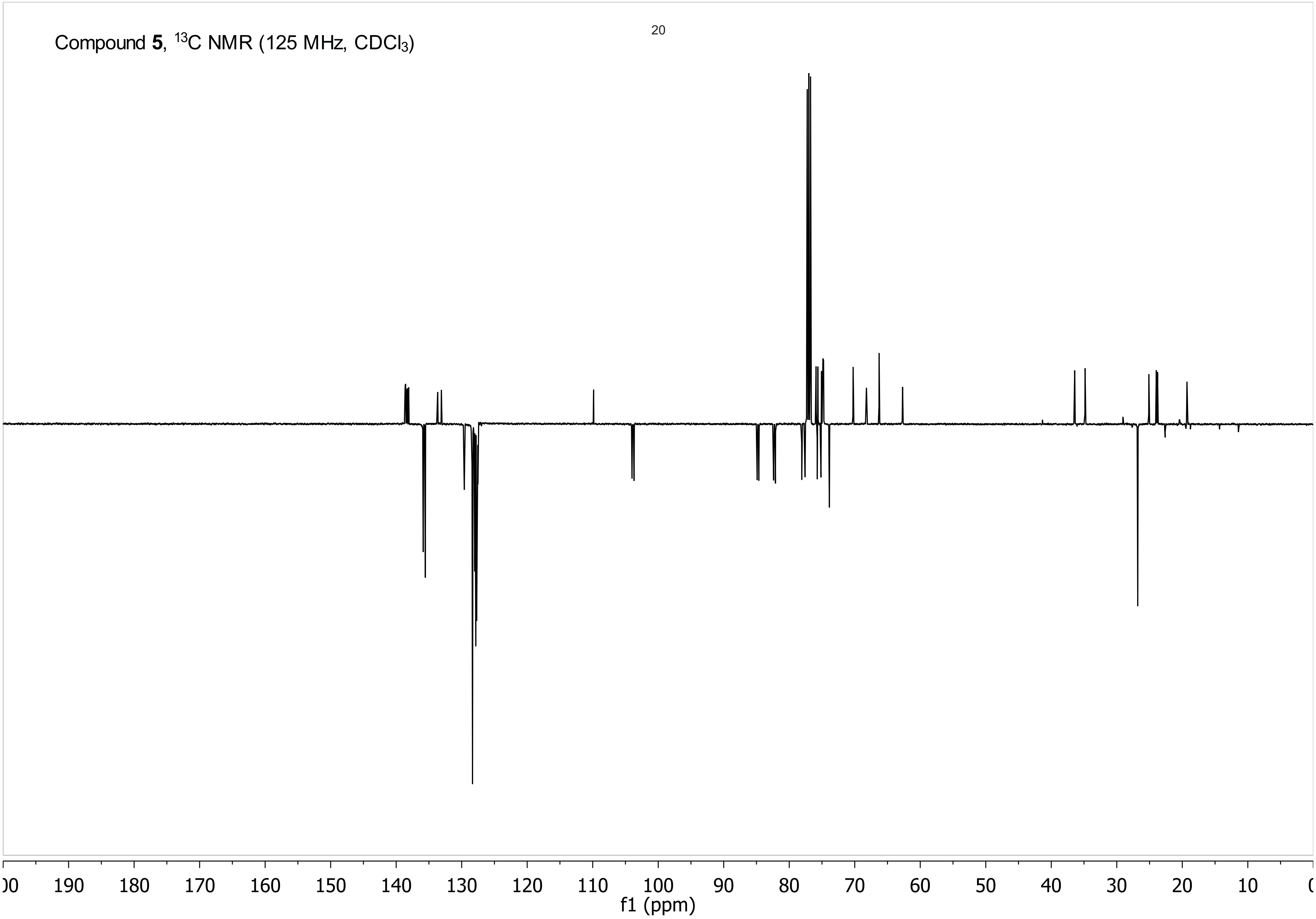
Compound **5**, ^{13}C NMR (125 MHz, $CDCl_3$)
190 180 170 160 150 140 130 120 110 100 90 80 70 60 50 40 30 20 10
f1 (ppm)

Compound **6**, ^{1}H NMR (500 MHz, $CDCl_3$)

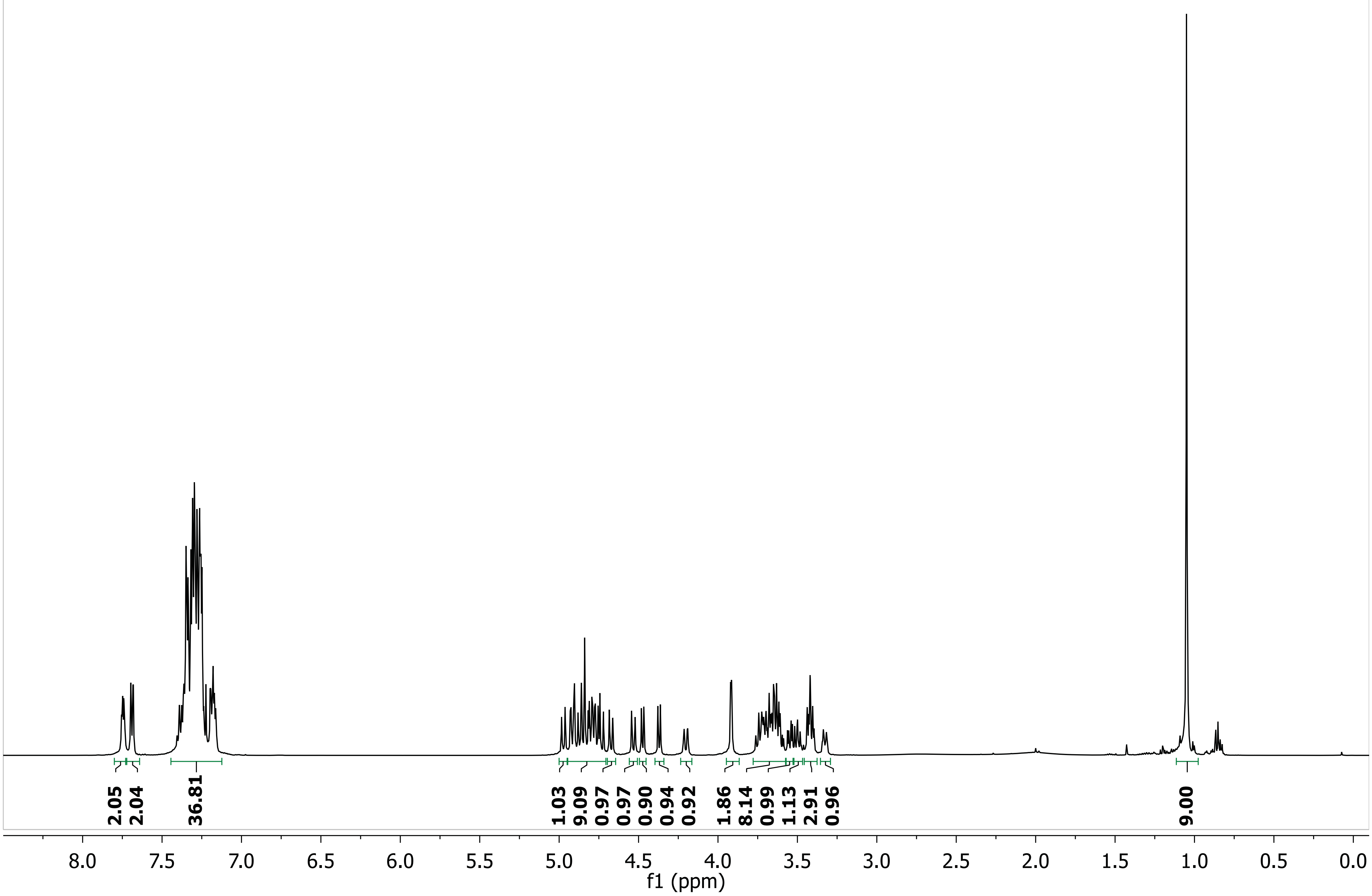

Compound **6**, ^{13}C NMR (125 MHz, $CDCl_3$)

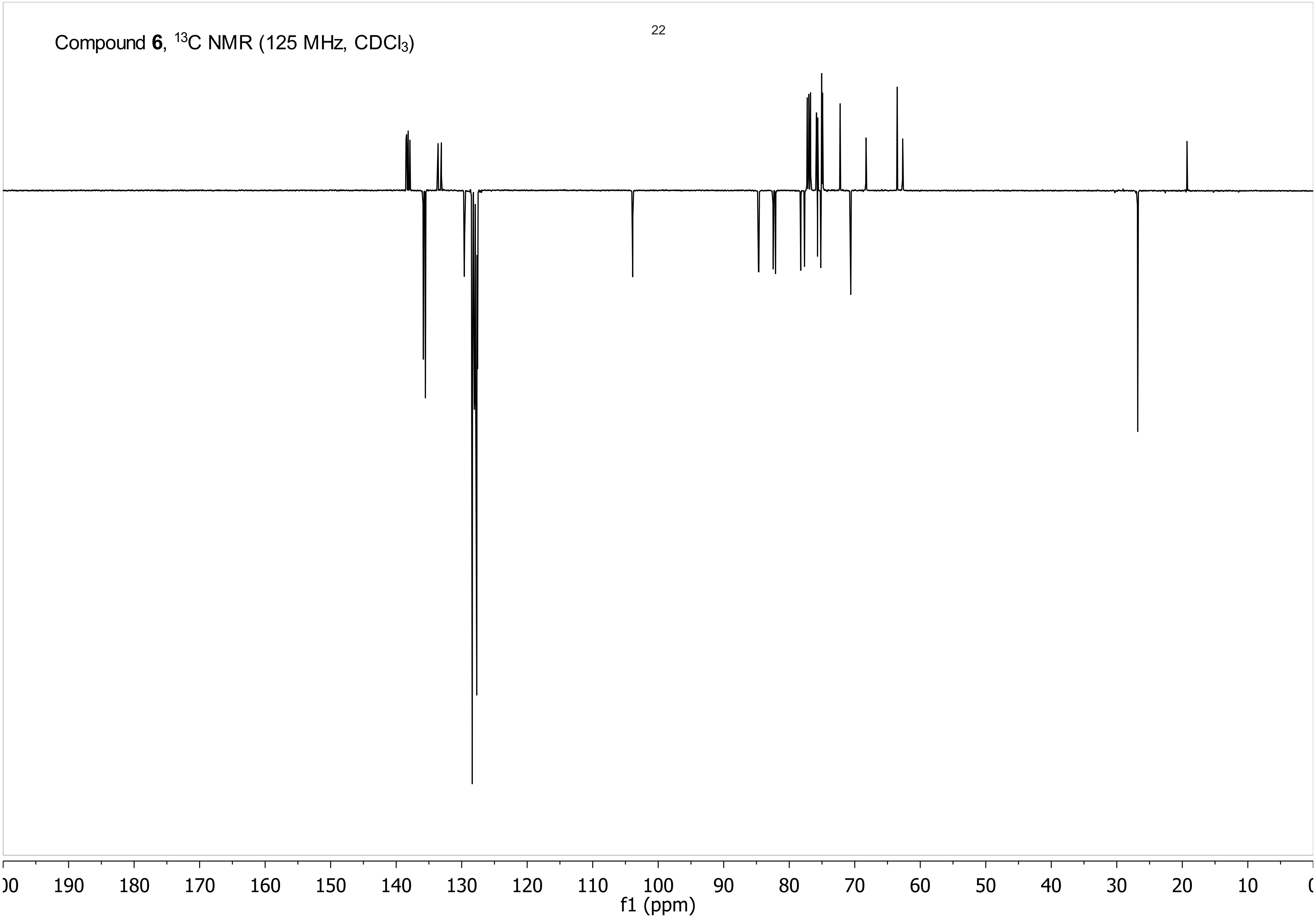

Intermediate C, ^1H NMR (500 MHz, $CDCl_3$)

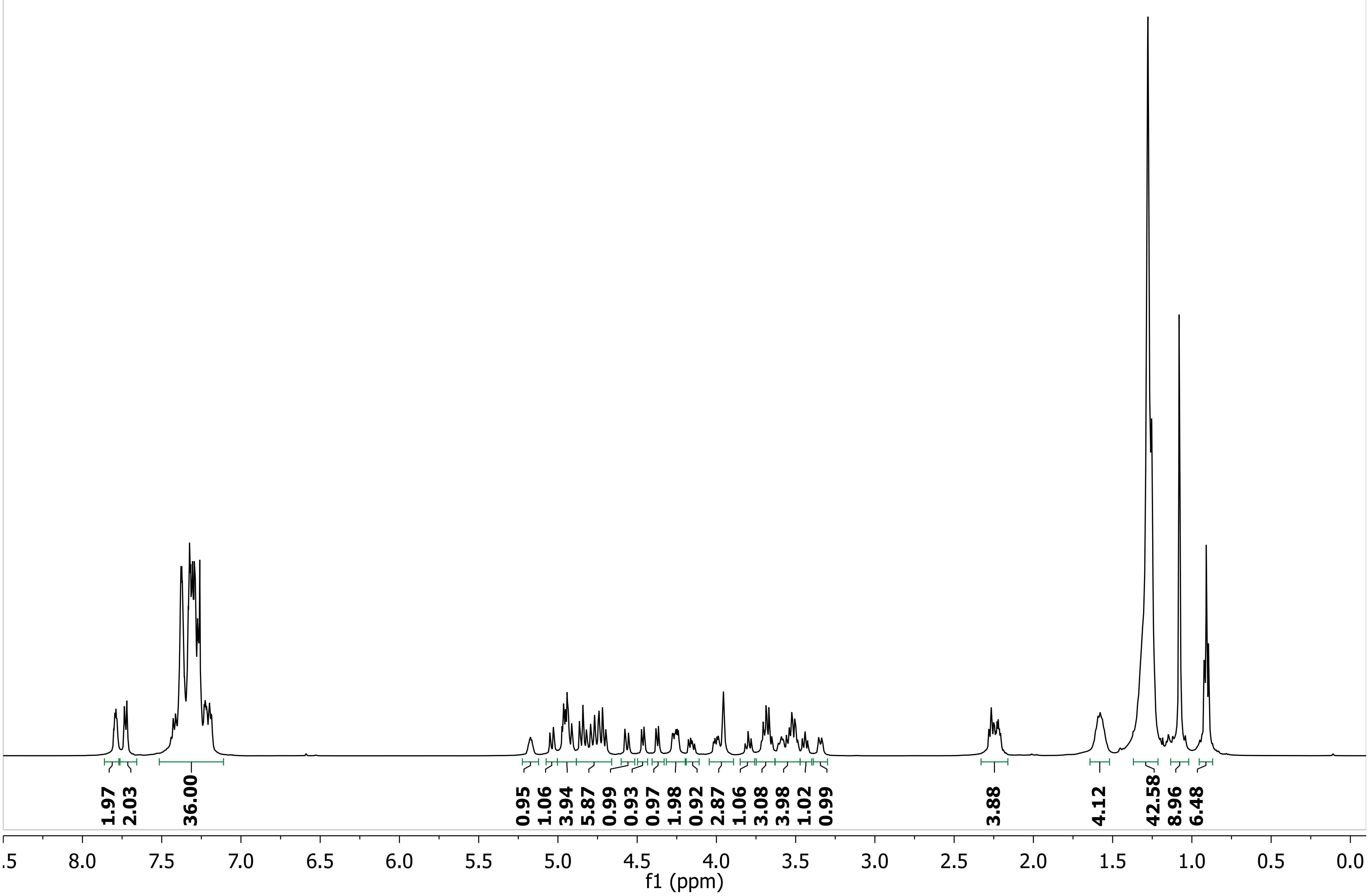

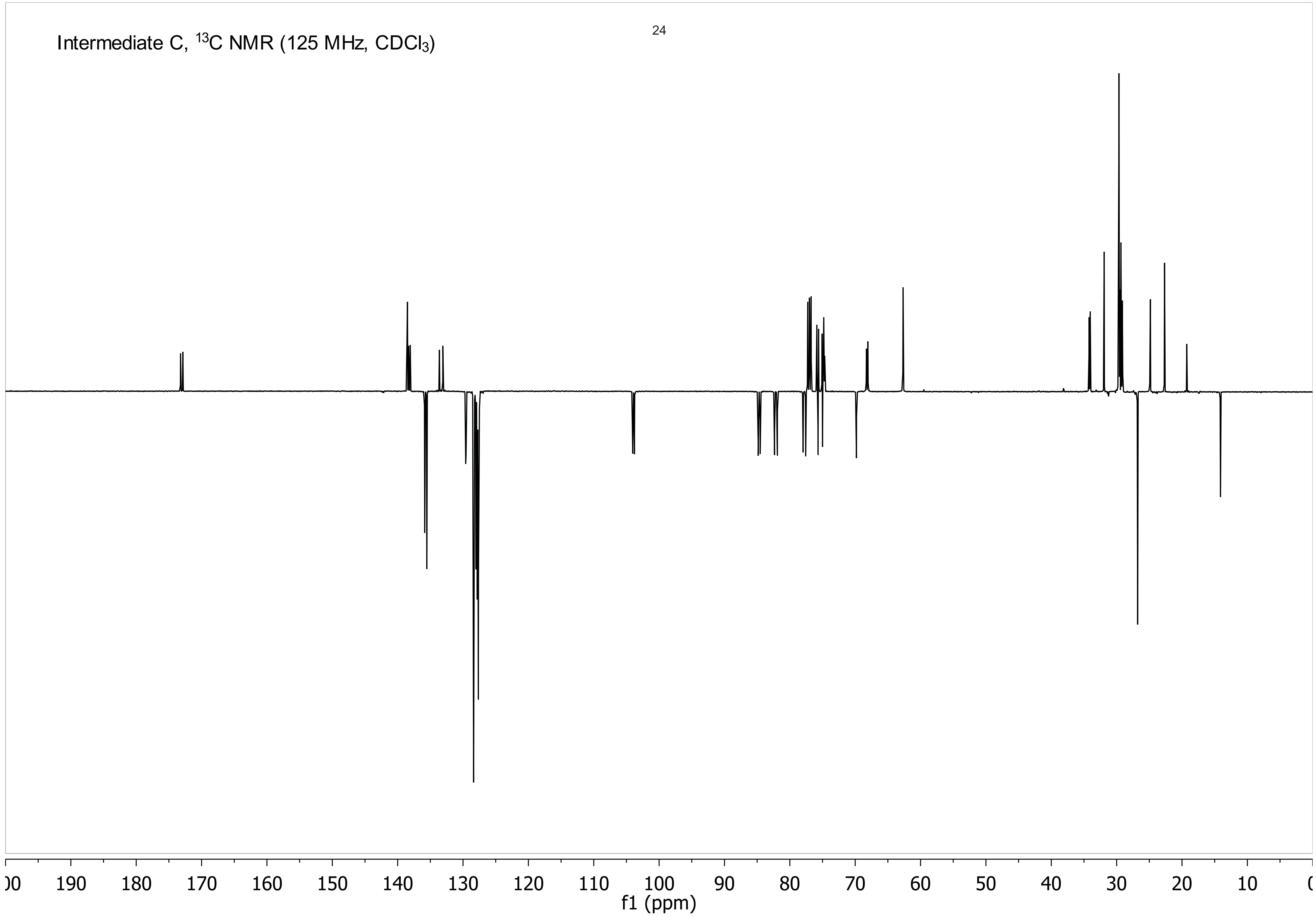
Intermediate C, ^{13}C NMR (125 MHz, $CDCl_3$)
190
180
170
160
150
140
130
120
110
100
90
80
70
60
50
40
30
20
10
f1 (ppm)

Compound **7**, ^{1}H NMR (500 MHz, $CDCl_3$)

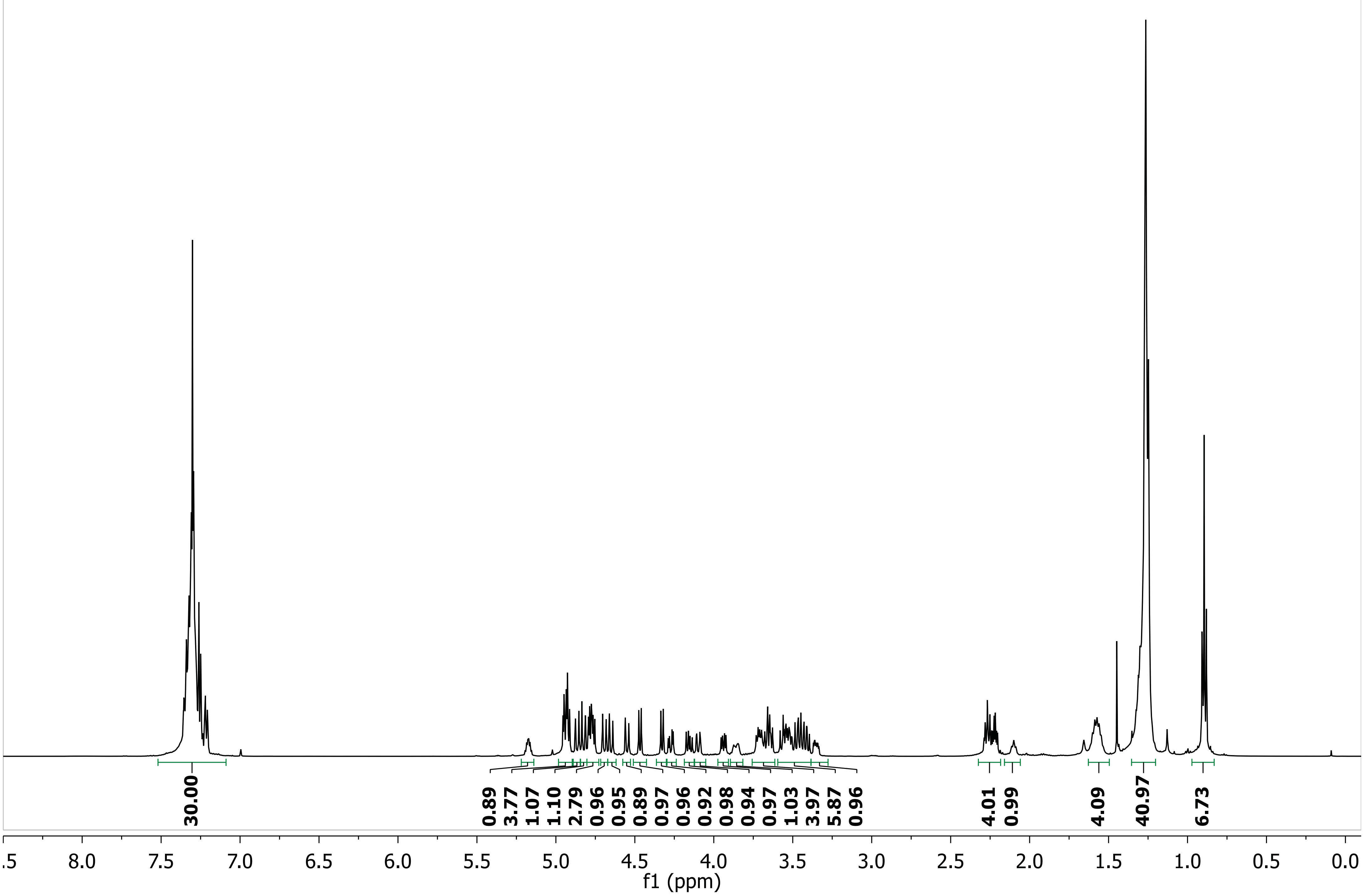

Compound **7**, ^{13}C NMR (125 MHz, $CDCl_3$)

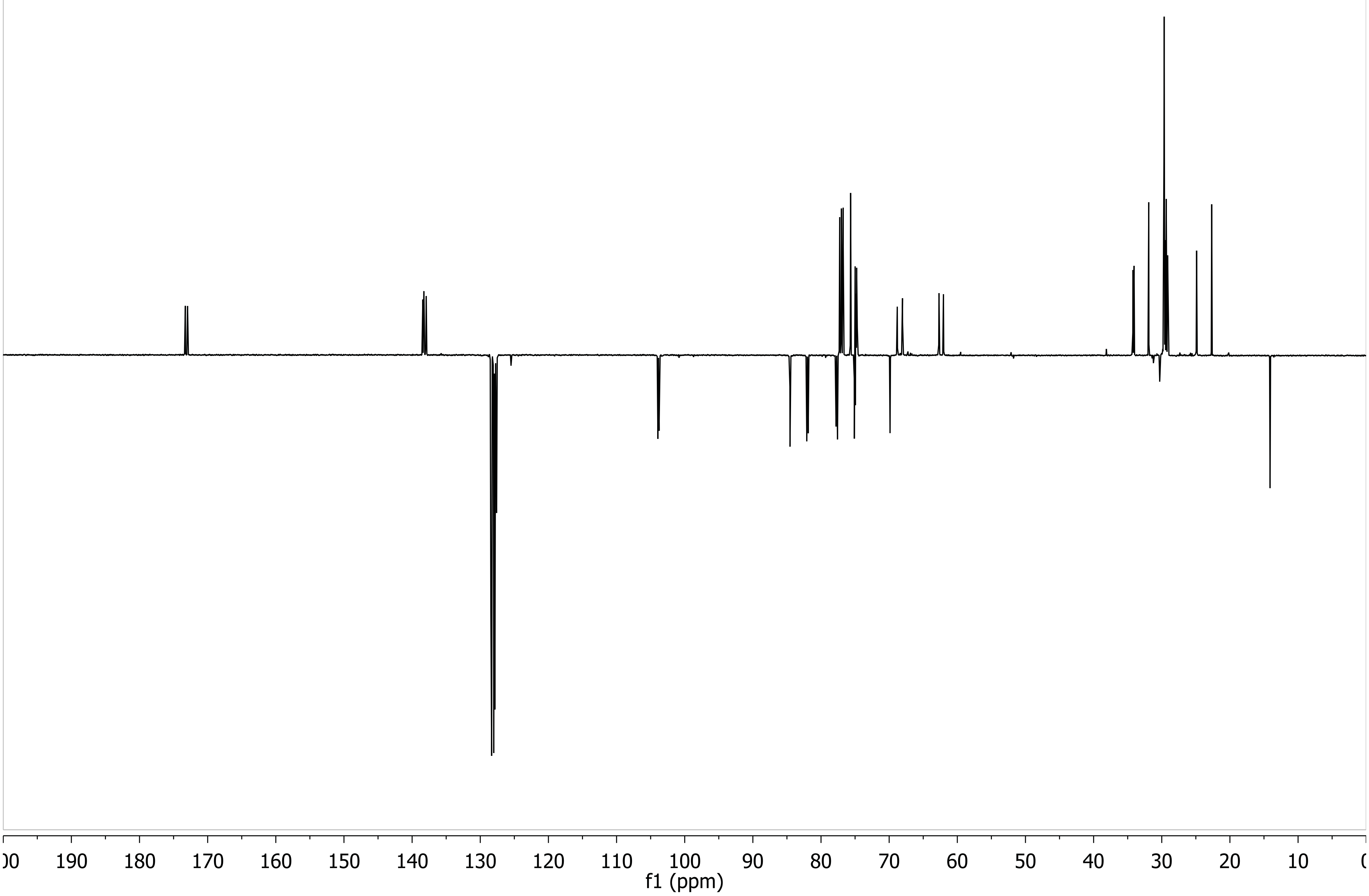

Compound **8**, ^{1}H NMR (500 MHz, $CDCl_3$)

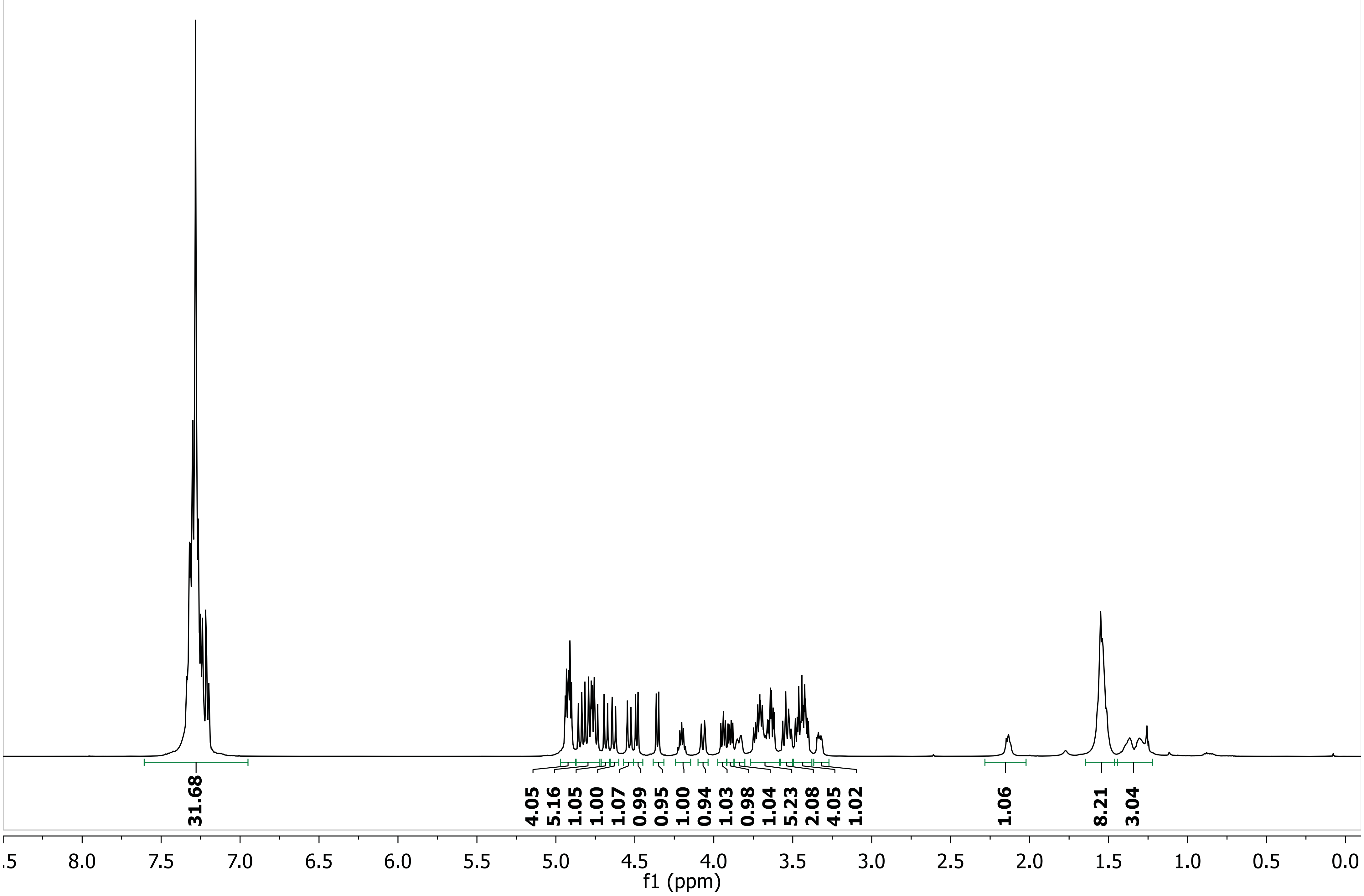

Compound **8**, ^{13}C NMR (125 MHz, $CDCl_3$)

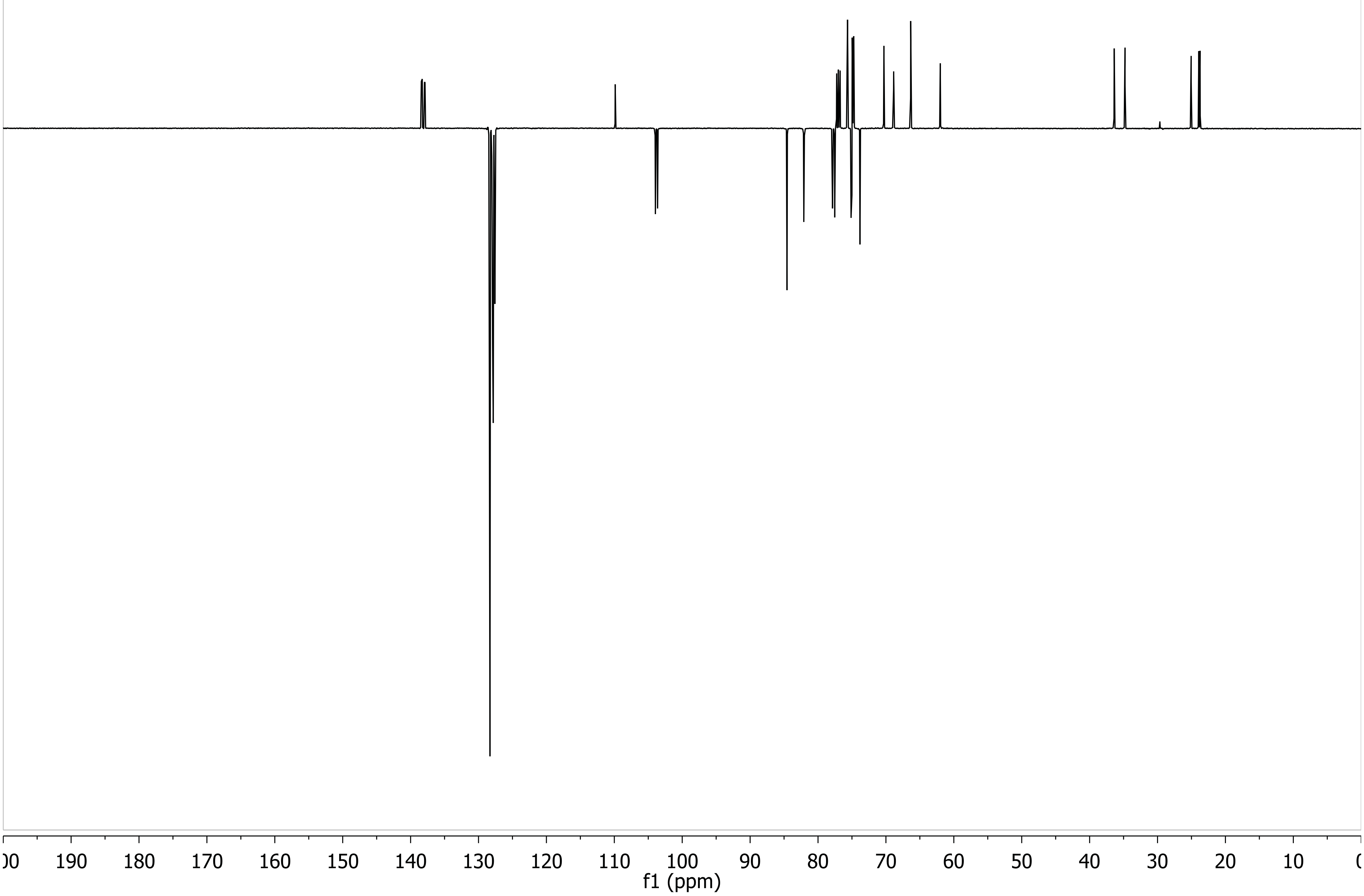

Compound **13**, ^{1}H NMR (500 MHz, $CDCl_3$)

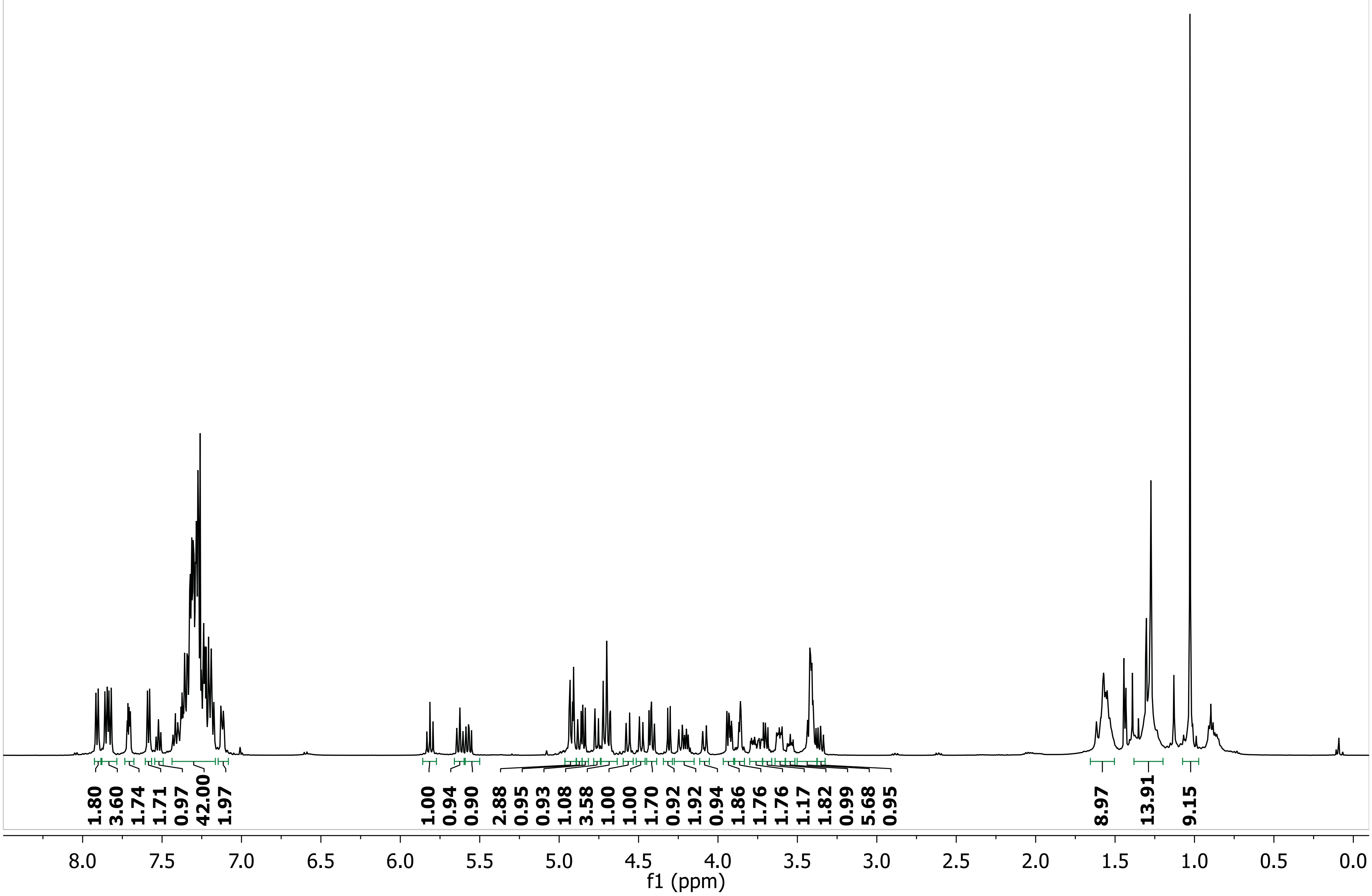

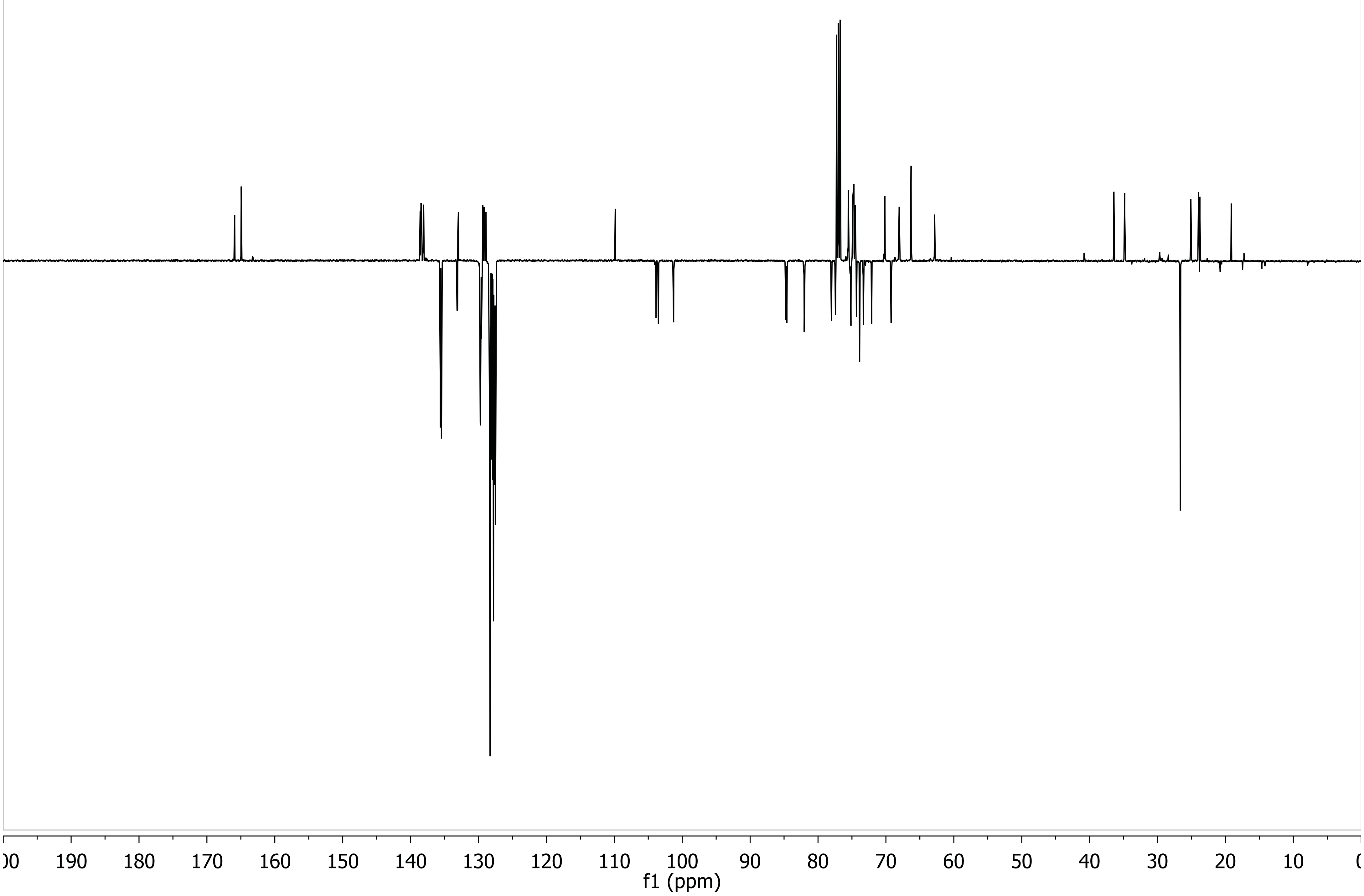

Compound **13**, ^{13}C NMR (125 MHz, $CDCl_3$)

Intermediate D, ^{1}H NMR (500 MHz, $CDCl_3$)

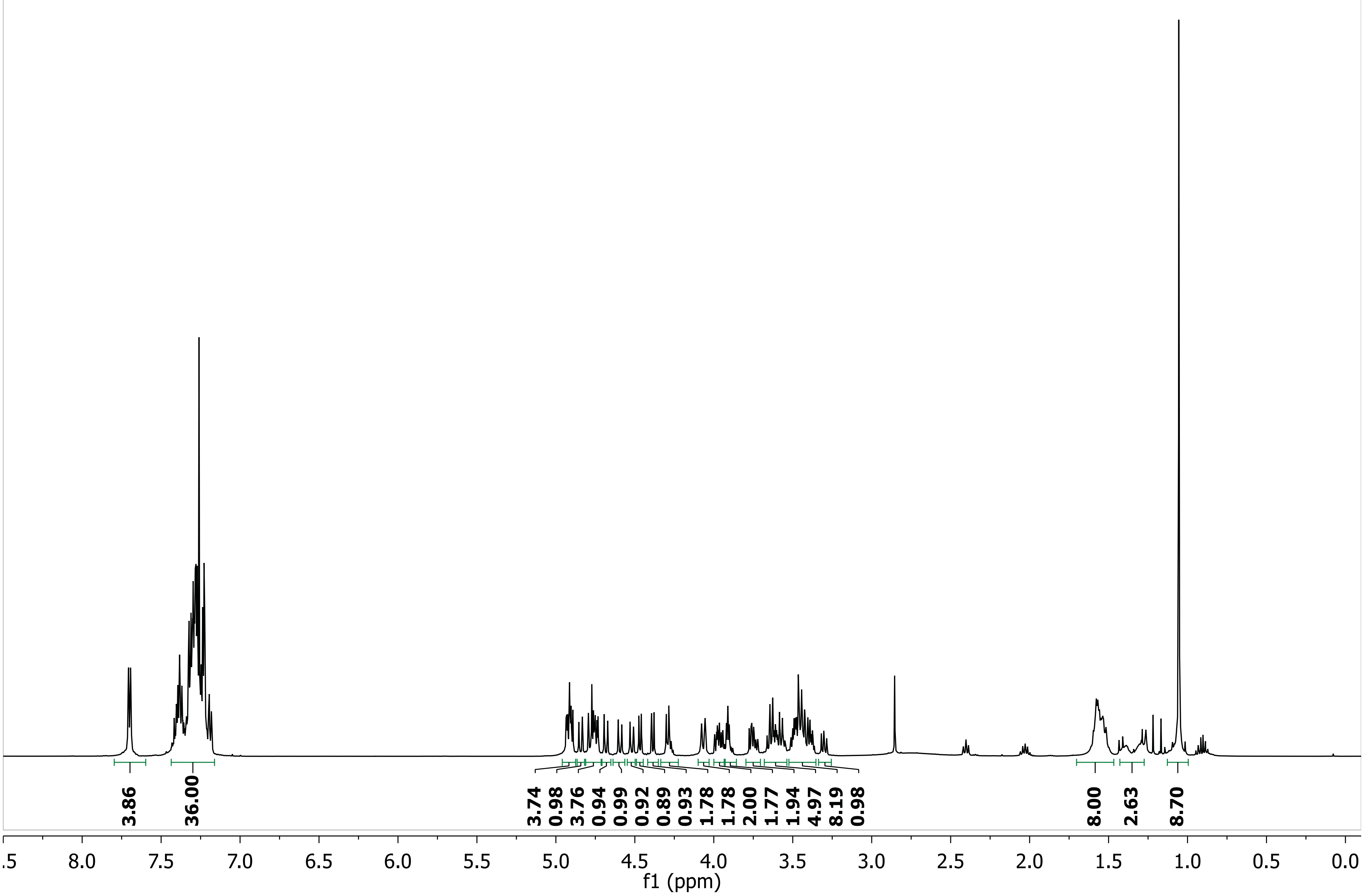

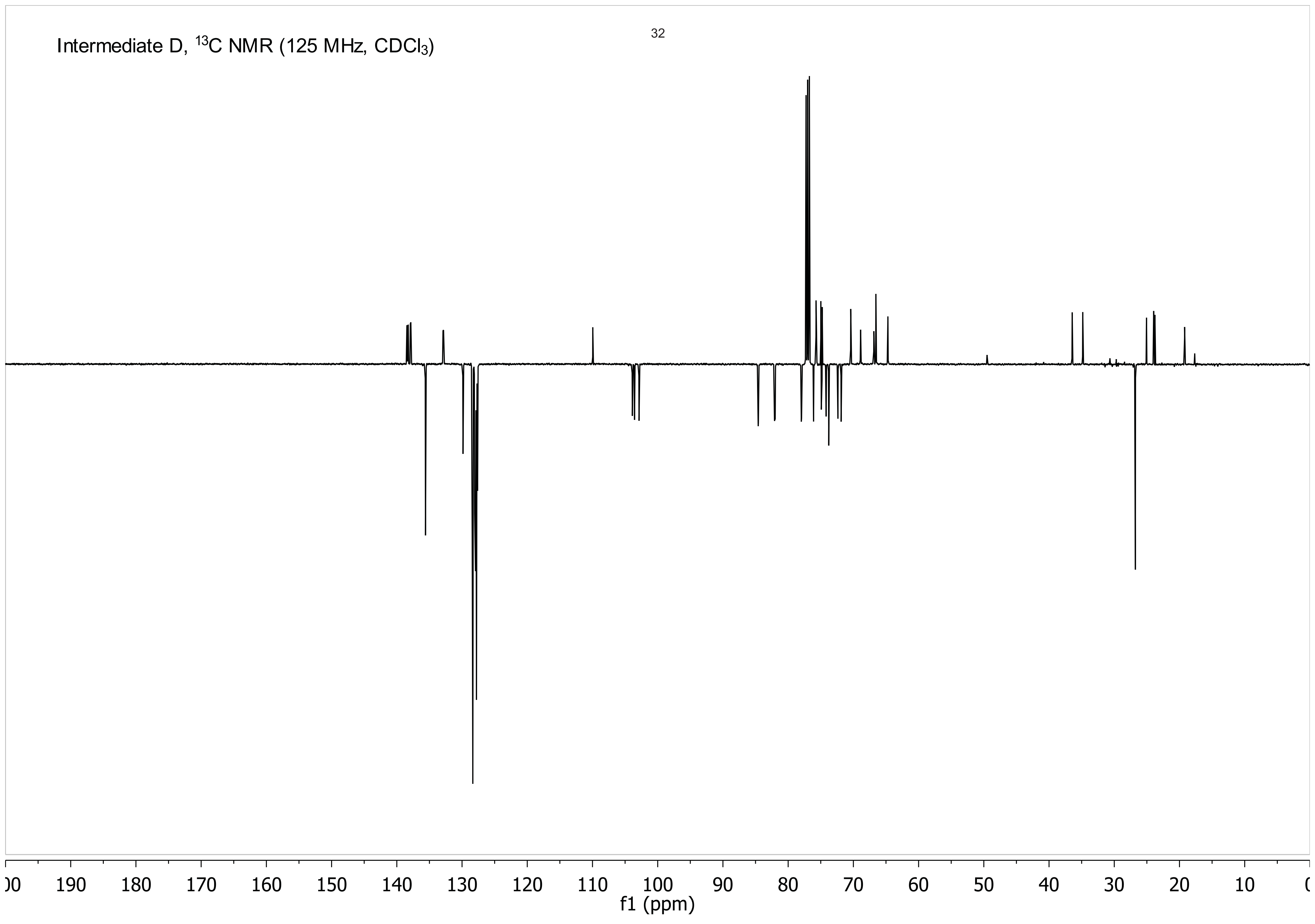
Intermediate D, ^{13}C NMR (125 MHz, $CDCl_3$)
190
180
170
160
150
140
130
120
110
100
90
80
70
60
50
40
30
20
10
f1 (ppm)

Intermediate E, ^{1}H NMR (500 MHz, $CDCl_3$)

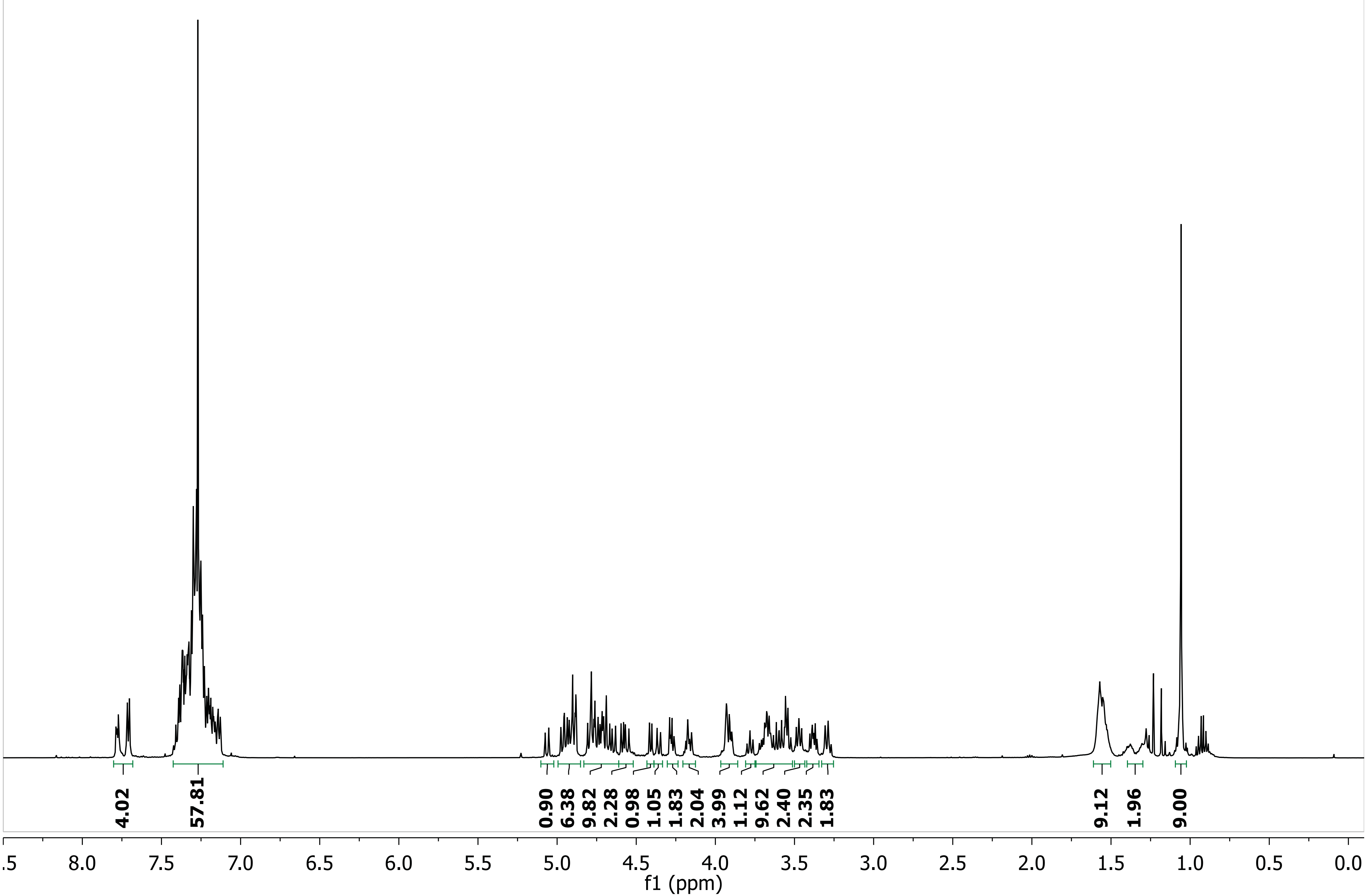

Intermediate E, ^{13}C NMR (125 MHz, $CDCl_3$)

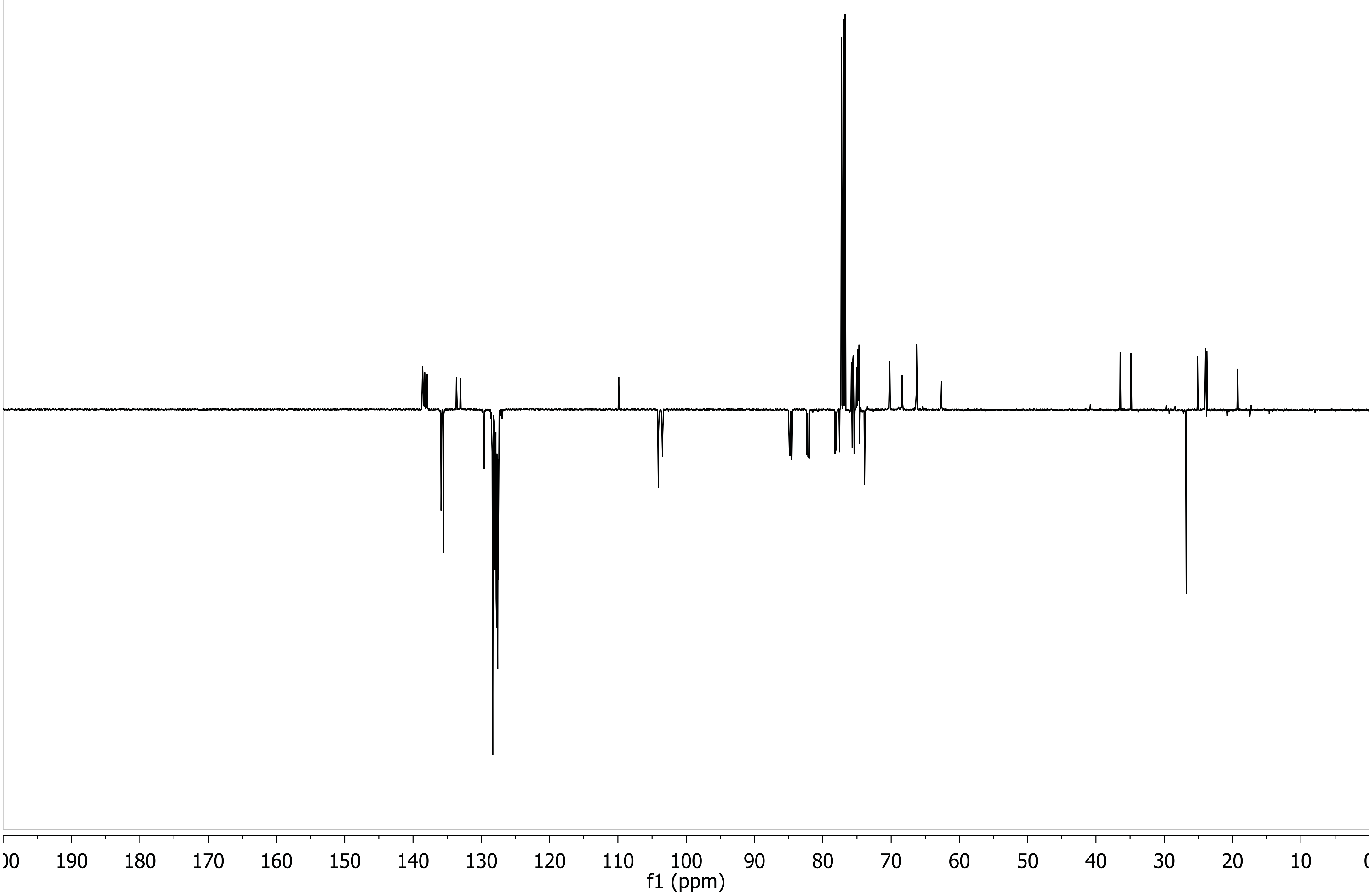

Intermediate F, ^{1}H NMR (500 MHz, $CDCl_3$)

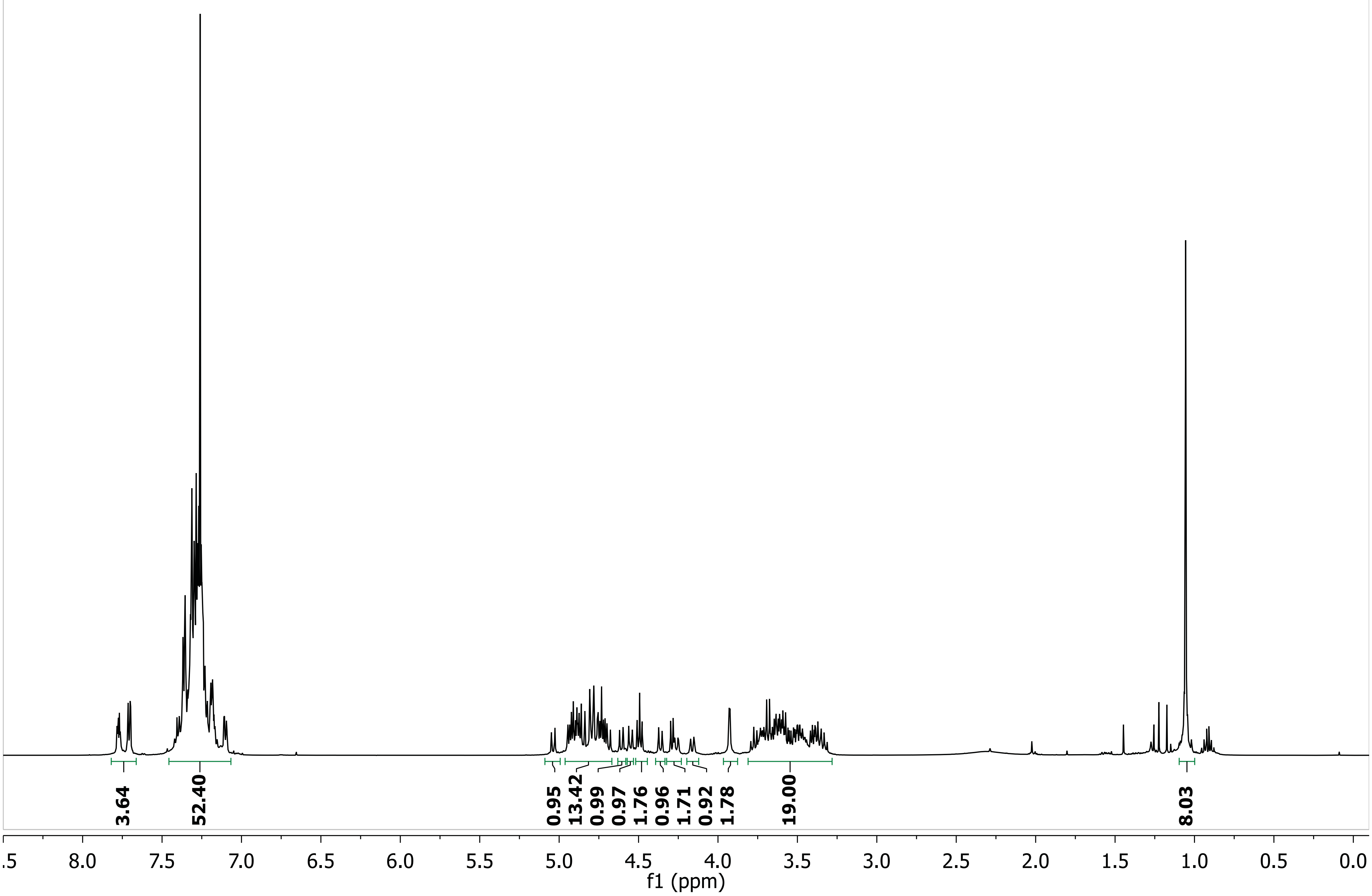

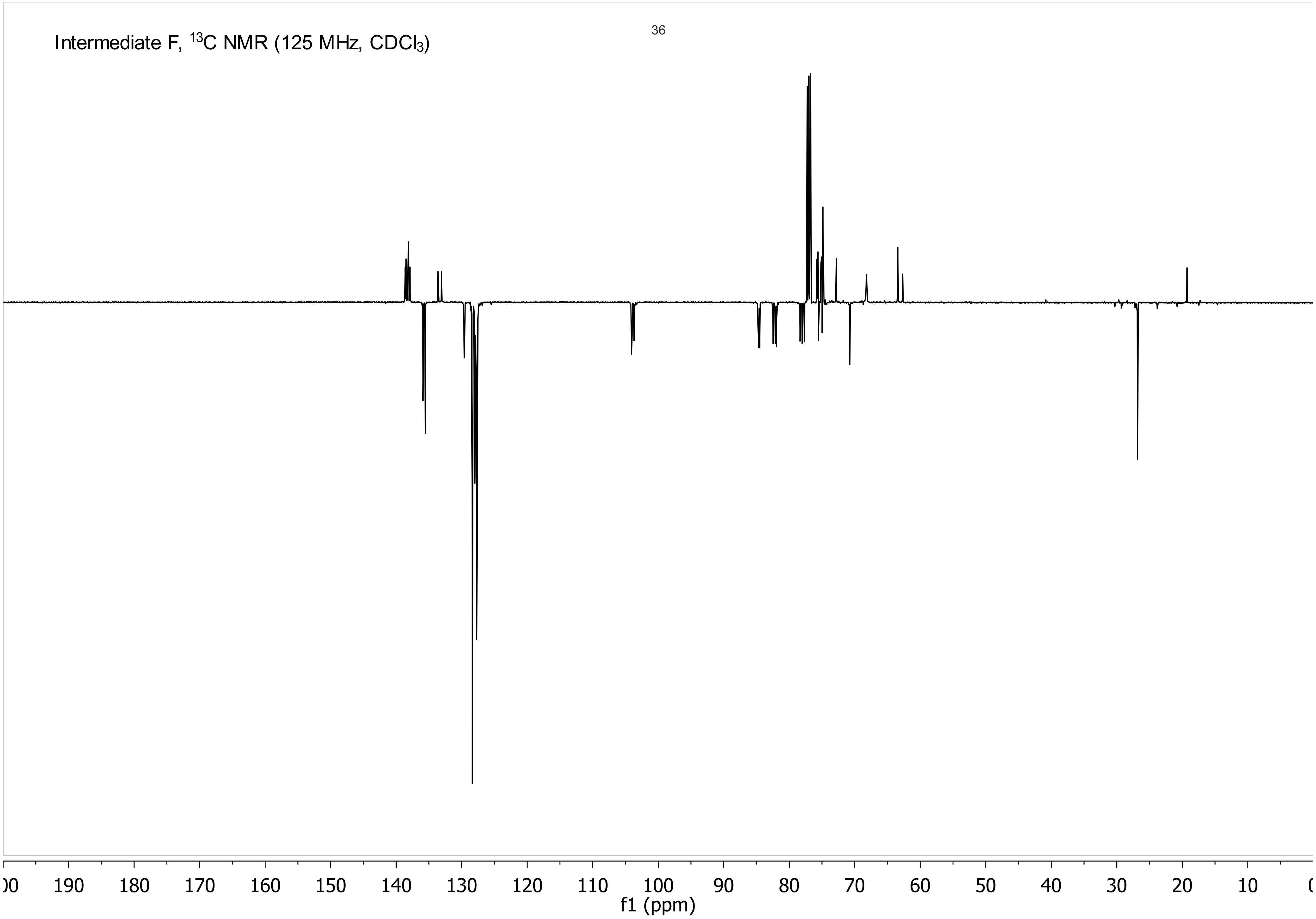
Intermediate F, ^{13}C NMR (125 MHz, $CDCl_3$)
200 190 180 170 160 150 140 130 120 110 100 90 80 70 60 50 40 30 20 10 0
f1 (ppm)

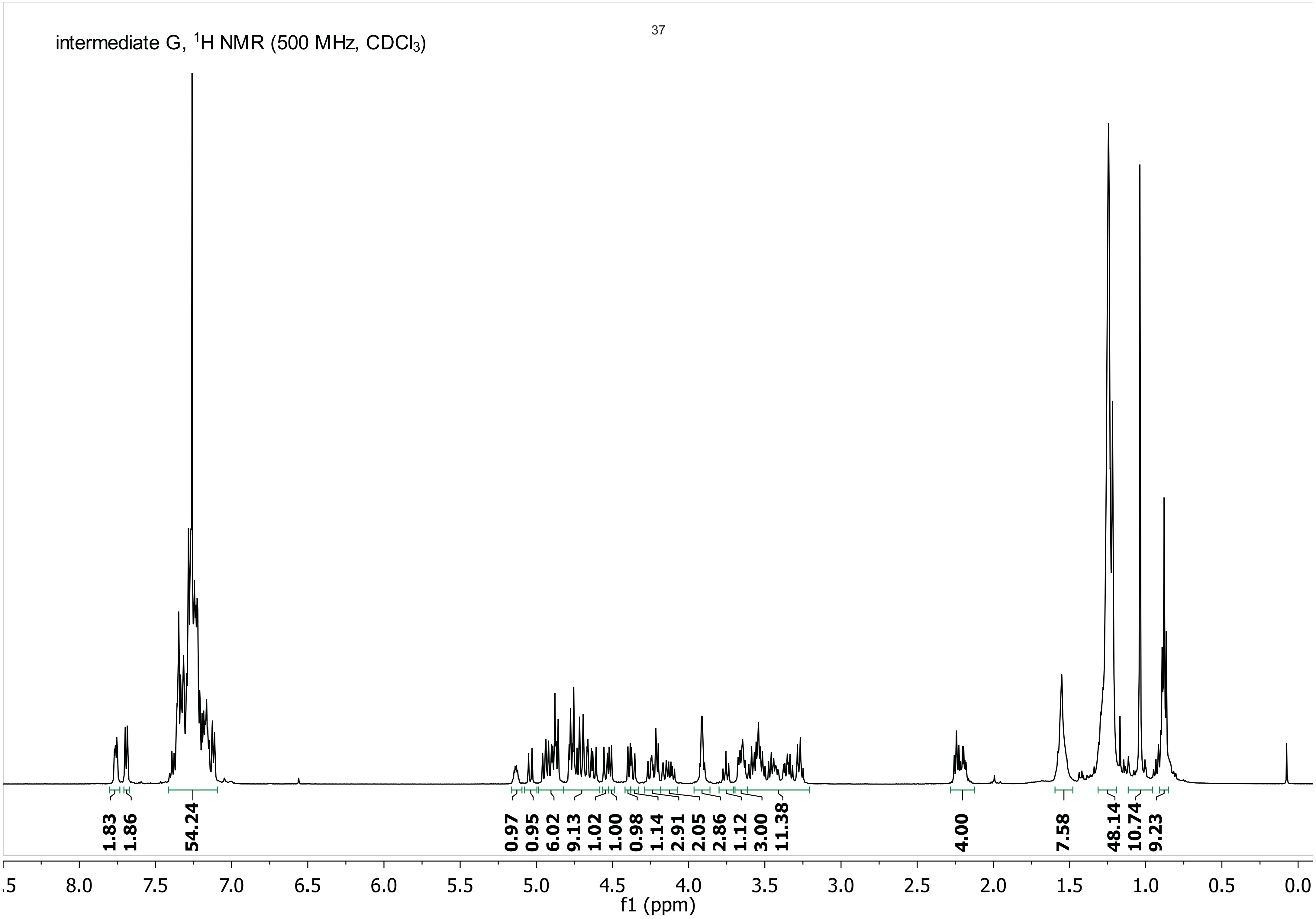
intermediate G, ^1H NMR (500 MHz, $CDCl_3$)
1.83
1.86
54.24
0.97
0.95
6.02
9.13
1.02
1.00
0.98
1.14
2.91
2.05
2.86
1.12
3.00
11.38
4.00
7.58
48.14
10.74
9.23
.5
8.0
7.5
7.0
6.5
6.0
5.5
5.0
4.5
4.0
3.5
3.0
2.5
2.0
1.5
1.0
0.5
0.0
f1 (ppm)

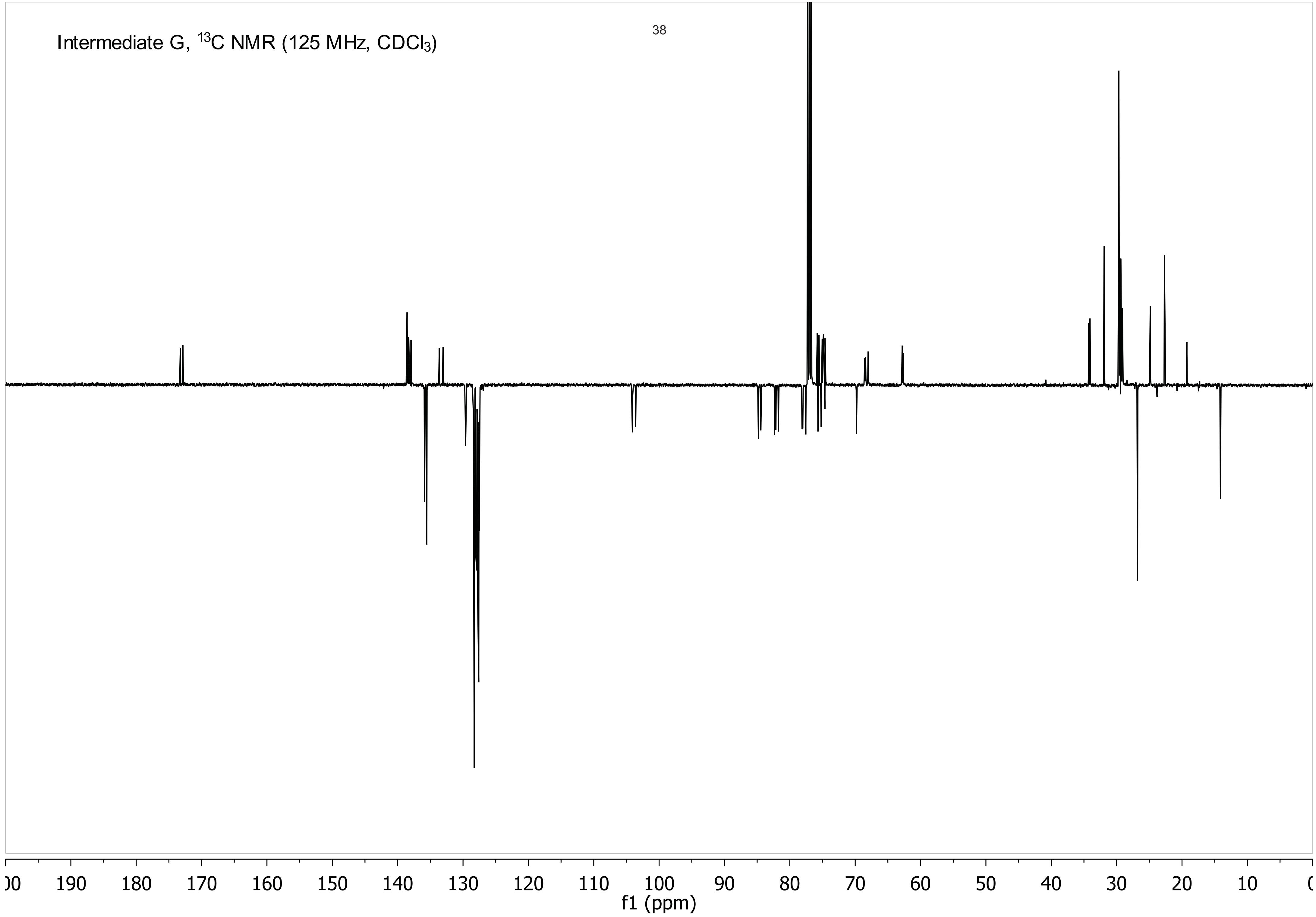
Intermediate G, ^{13}C NMR (125 MHz, $CDCl_3$)
190 180 170 160 150 140 130 120 110 100 90 80 70 60 50 40 30 20 10
f1 (ppm)

Compound **14**, ^{1}H NMR (500 MHz, $CDCl_3$)

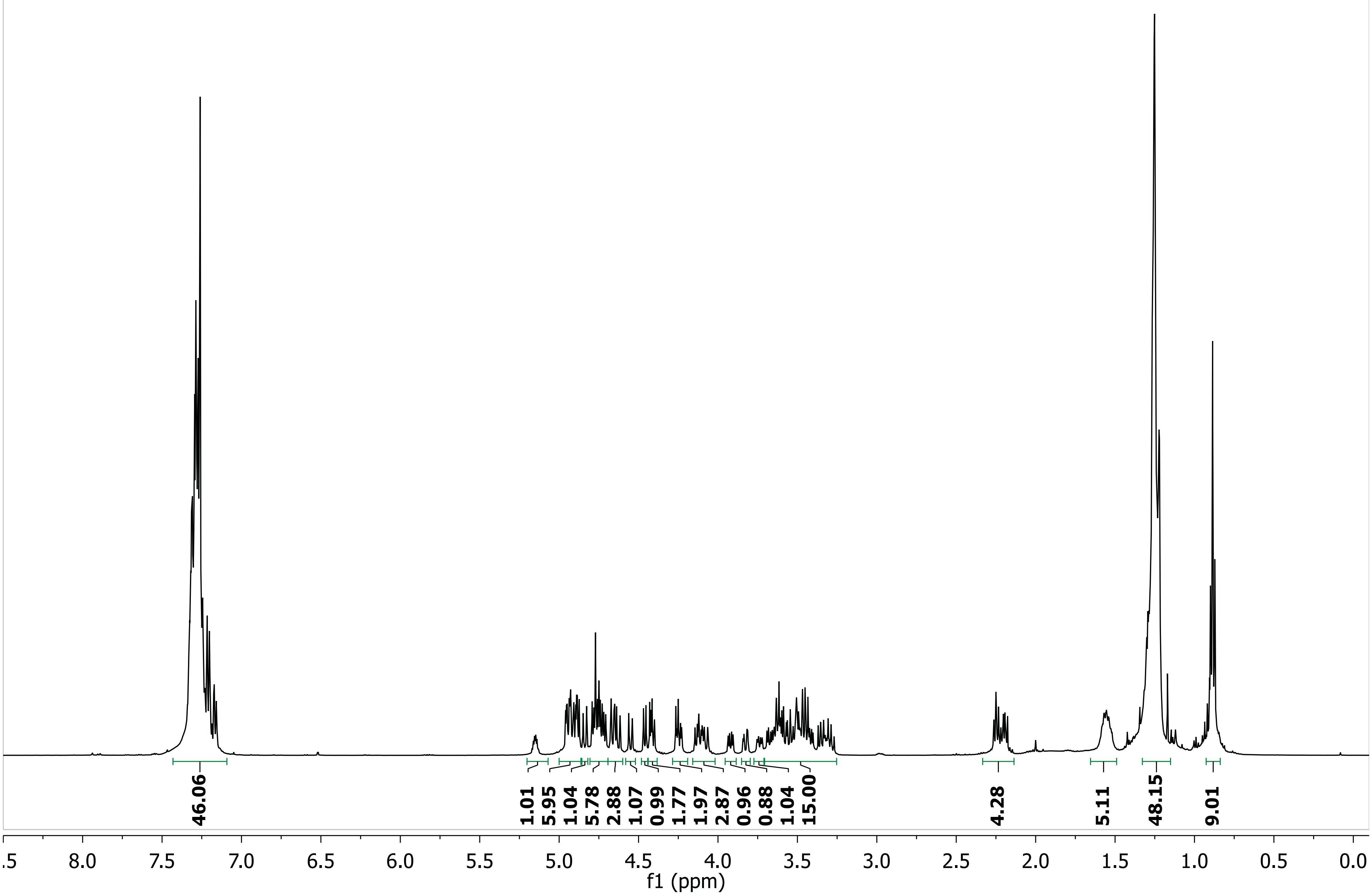

Compound **14**, ^{13}C NMR (125 MHz, $CDCl_3$)

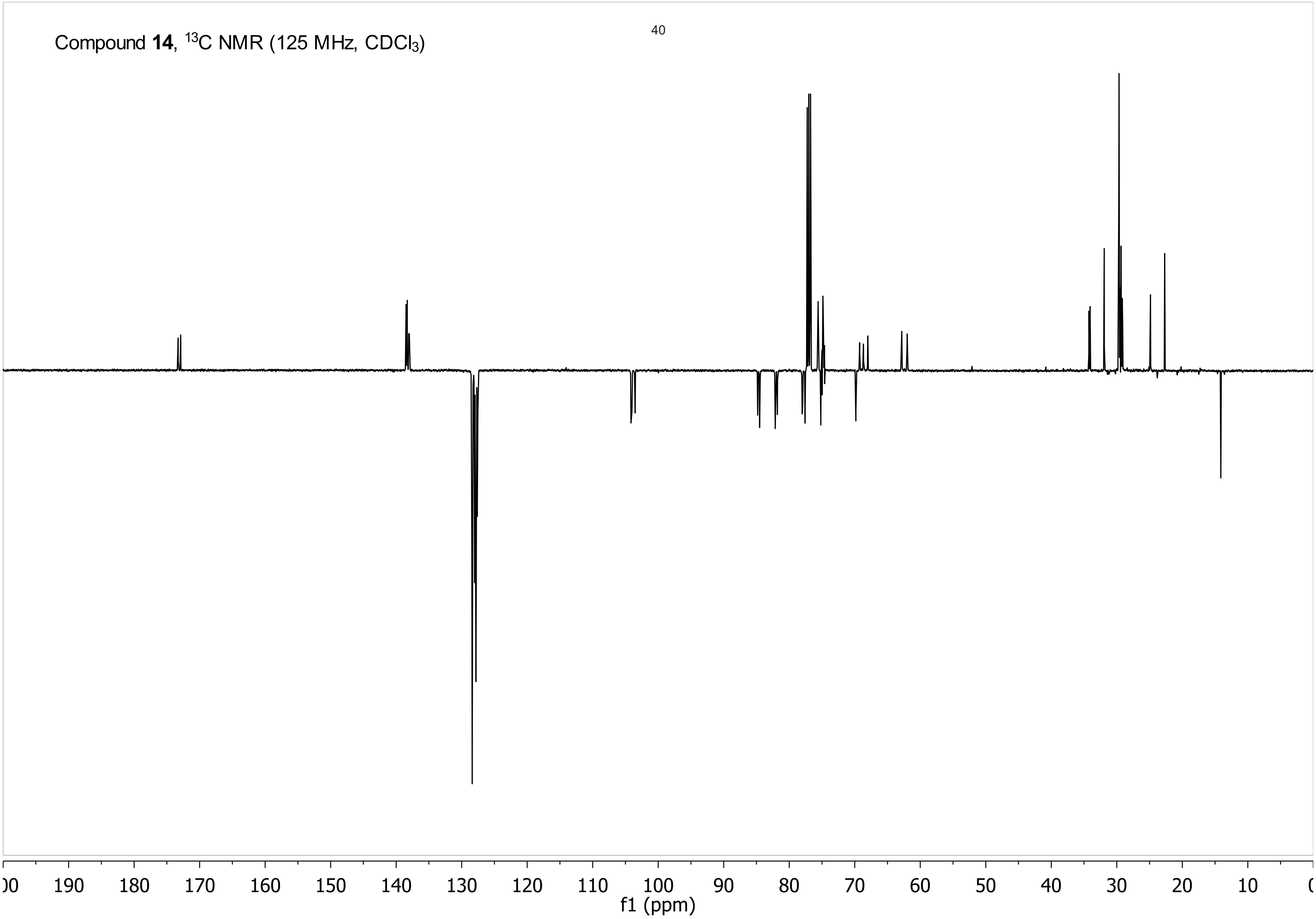

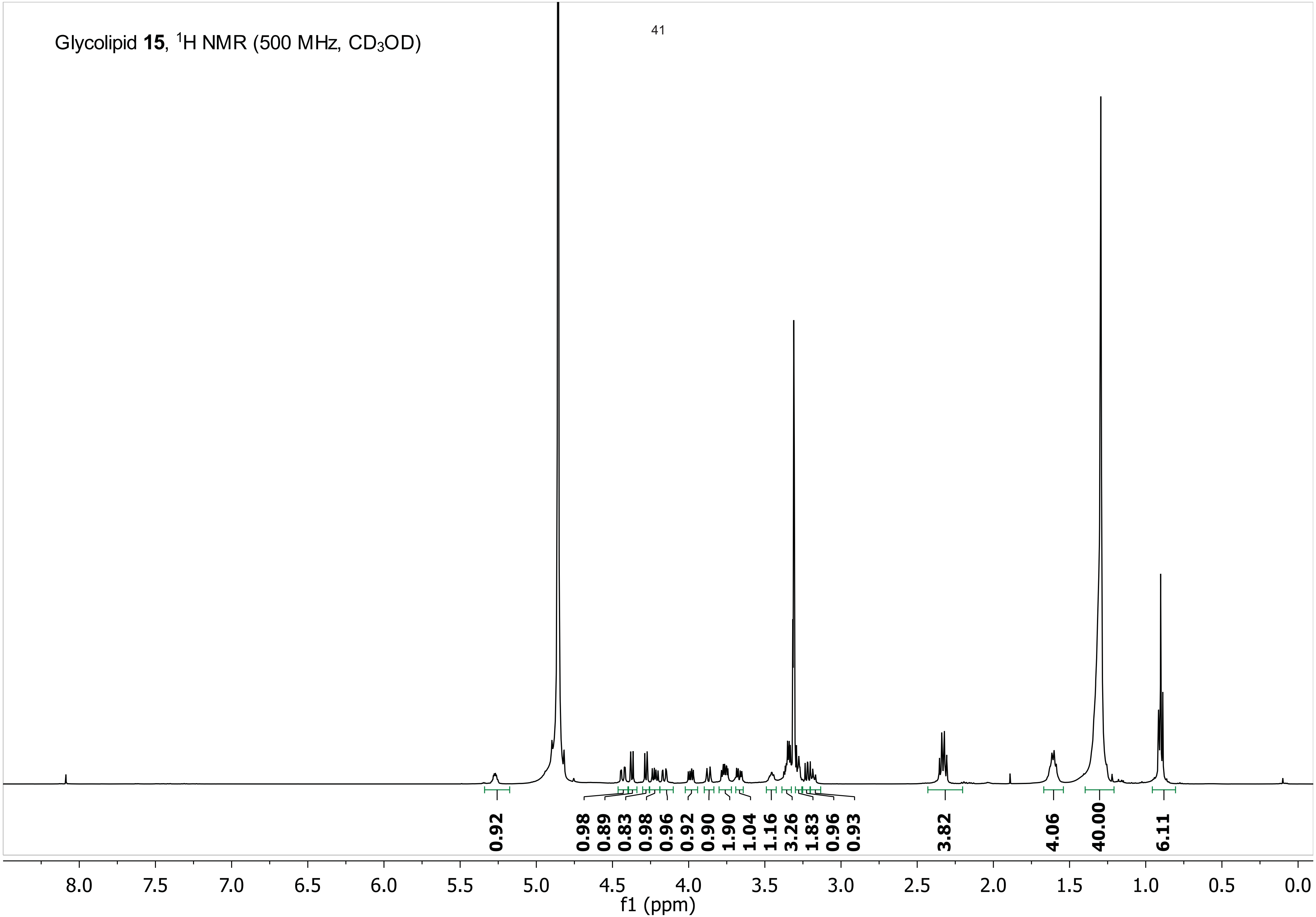

Glycolipid 15, ¹H NMR (500 MHz, CD₃OD)
0.92
0.98
0.89
0.83
0.98
0.96
0.92
0.90
1.90
1.04
1.16
3.26
1.83
0.96
0.93
3.82
4.06
40.00
6.11
8.0
7.5
7.0
6.5
6.0
5.5
5.0
4.5
4.0
3.5
3.0
2.5
2.0
1.5
1.0
0.5
0.0
f1 (ppm)

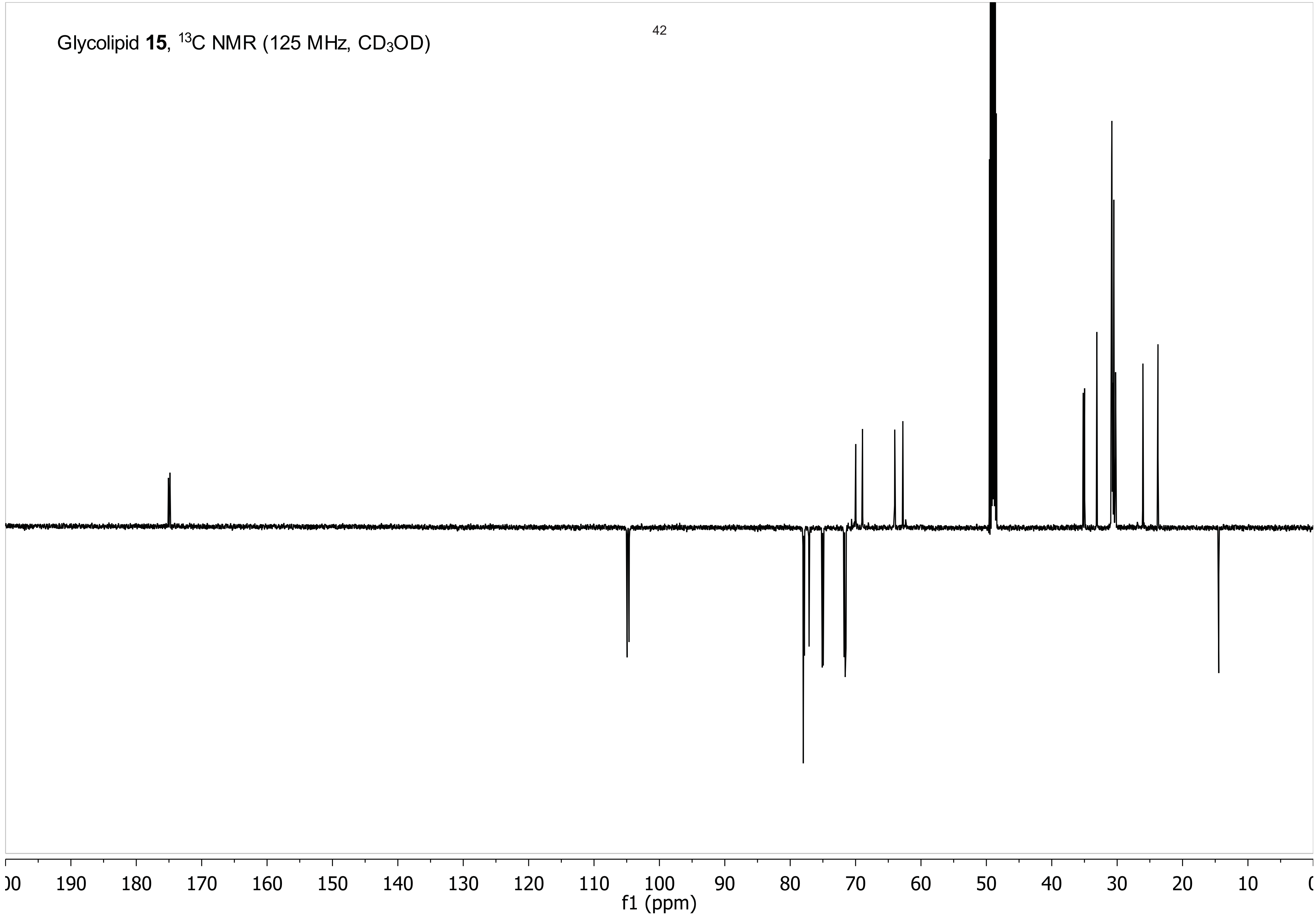
Glycolipid **15**, ^{13}C NMR (125 MHz, CD_3OD)
00 190 180 170 160 150 140 130 120 110 100 90 80 70 60 50 40 30 20 10 0
f1 (ppm)

Glycolipid **16**, ^1H NMR (500 MHz, CD_3OD)

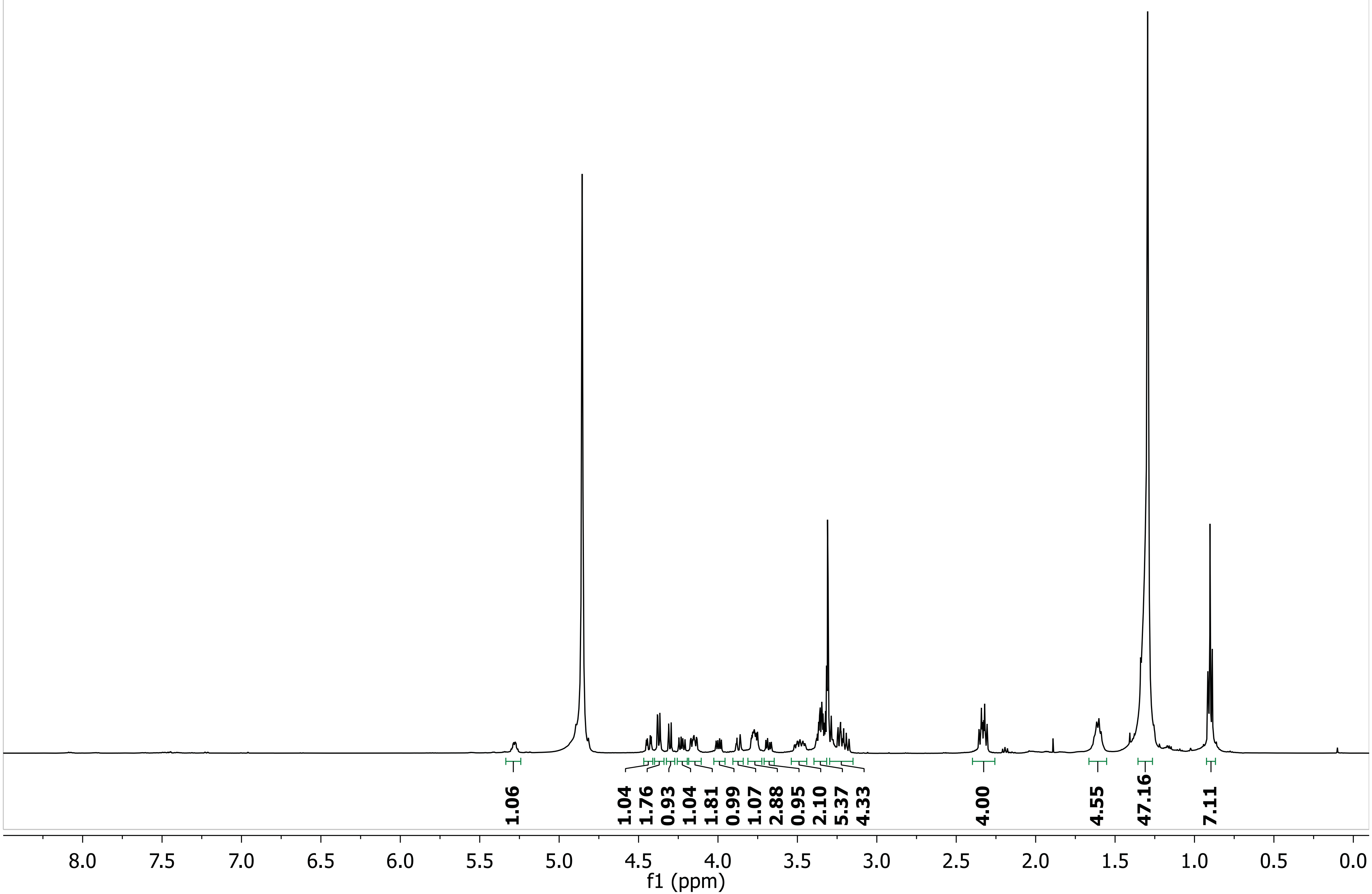

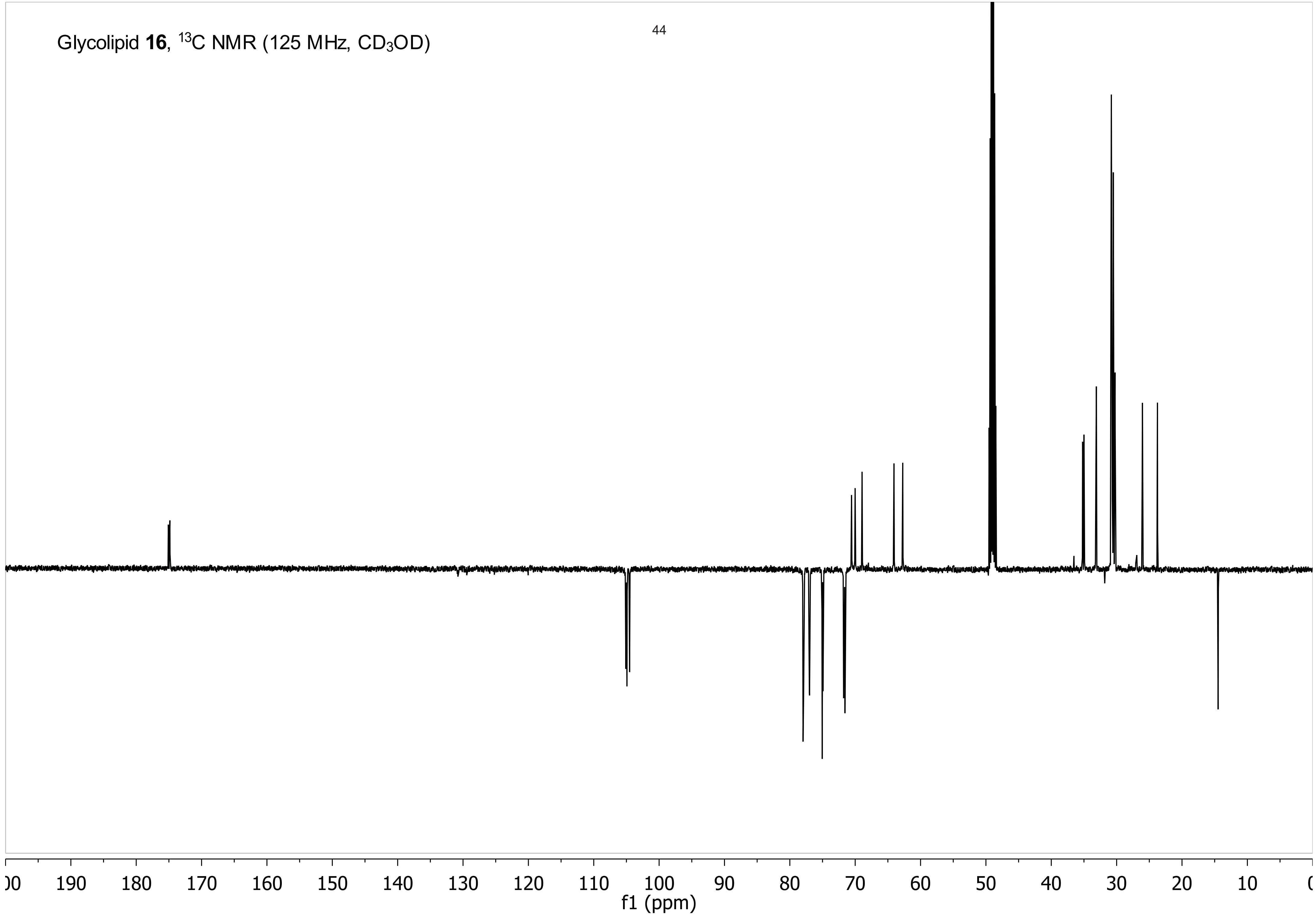
Glycolipid **16**, ^{13}C NMR (125 MHz, CD_3OD)
190 180 170 160 150 140 130 120 110 100 90 80 70 60 50 40 30 20 10
f1 (ppm)
